# M1-linked ubiquitination by LUBAC regulates AMPK signalling and the response to energetic stress

**DOI:** 10.1038/s41418-026-01675-z

**Published:** 2026-02-13

**Authors:** Camilla Reiter Elbæk, Sophie Gradinaru, Anna M. Dahlström, Alexander Frueh, Akhee S. Jahan, Joyceline Cuenco, Anna L. Aalto, John Rizk, Simon A. Hawley, Sarah N. J. Franks, Michael Stumpe, Srinivasa Prasad Kolapalli, Chris Kedong Wang, Cara J. Ellison, Ximena Hildebrandt, Klara Nielsen, Dominik Priesmann, Julian Koch, Mathilde Deichmann, Josef Gullmets, Lien Verboom, Geert van Loo, Nieves Peltzer, Lisa B. Frankel, Paul R. Elliott, Mads Gyrd-Hansen, Jörn Dengjel, Brent J. Ryan, D. Grahame Hardie, Annika Meinander, Kei Sakamoto, Rune Busk Damgaard

**Affiliations:** 1 Department of Biotechnology and Biomedicine, Technical University of Denmark, Kongens Lyngby, Denmark; 2 Biochemistry and Cell Biology, Faculty of Science and Engineering, Åbo Akademi University, Turku, Finland; 3 Novo Nordisk Foundation Center for Basic Metabolic Research, University of Copenhagen, Copenhagen, Denmark; 4 LEO Foundation Skin Immunology Research Center, Department of Immunology and Microbiology, University of Copenhagen, Copenhagen, Denmark; 5 Division of Cell Signalling and Immunology, School of Life Sciences, University of Dundee, Dundee, Scotland UK; 6 Oxford Parkinson’s Disease Centre and Department of Physiology, Anatomy and Genetics, University of Oxford, Oxford, UK; 7 Kavli Institute for Nanoscience Discovery, University of Oxford, Oxford, UK; 8 Department of Biology, University of Fribourg, Fribourg, Switzerland; 9 Danish Cancer Institute, Copenhagen, Denmark; 10 Department of Biochemistry, University of Oxford, Oxford, UK; 11 Department of Genome Editing, Institute of Biomedical Genetics (IBMG), University of Stuttgart, Stuttgart, Germany; 12 Centre for Molecular Medicine Cologne (CMMC), University of Cologne, Cologne, Germany; 13 Cologne Excellence Cluster on Cellular Stress Responses in Aging-Associated Diseases (CECAD), Cologne, Germany; 14 VIB-Center for Inflammation Research, Ghent, Belgium; 15 Department of Biomedical Molecular Biology, Ghent University, Ghent, Belgium; 16 Biotech Research and Innovation Centre, Faculty of Health and Medical Sciences, University of Copenhagen, Copenhagen, Denmark; 17 Present Address: Centre for Genomic Regulation, Barcelona Institute of Science and Technology, Barcelona, Spain

**Keywords:** Cell biology, Biochemistry, Inflammation, Molecular biology

## Abstract

Methionine-1 (M1)–linked ubiquitin chains, assembled by the linear ubiquitin chain assembly complex (LUBAC) and disassembled by the deubiquitinase OTULIN, are critical regulators of inflammation and immune homoeostasis. Genetic loss or mutation of the LUBAC subunits HOIP and HOIL-1 or of OTULIN causes autoinflammatory syndromes accompanied by metabolic defects, including amylopectinosis, lipodystrophy, and fatty liver disease. Yet, it remains unclear how LUBAC and OTULIN control metabolic signalling. Here, we demonstrate that LUBAC and OTULIN dynamically regulate the energy-sensing kinase AMPK, a central sensor and switch for cellular and organismal energy balance. LUBAC’s activity through the catalytic subunit HOIP is required for full AMPK activation in response to energetic stress, whereas OTULIN antagonises this response. LUBAC and OTULIN form a complex with AMPK, and LUBAC can directly ubiquitinate AMPKα and β subunits in cells and in vitro, establishing AMPK as a bona fide M1-linked ubiquitin substrate. Loss of LUBAC blunts AMPK activation, reduces bioenergetic adaptability, impairs autophagy, and sensitises cells to starvation-induced death, while *Drosophila* lacking Lubel – the fly orthologue of LUBAC – exhibit defective AMPK activation and reduced survival during starvation. Our findings identify M1-linked ubiquitination as a previously unrecognised regulatory layer controlling AMPK activation, metabolic adaptability, and the cellular response to energetic stress.

## Introduction

Methionine-1 (M1)-linked ubiquitin (Ub) chains (also called ‘linear’ ubiquitin chains) are important for the regulation of immune signalling and inflammation [1]. M1-linked Ub is specifically assembled by the linear ubiquitin chain assembly complex (LUBAC), consisting of the catalytic subunit HOIP, HOIL-1 and SHARPIN [1, 2], and is specifically disassembled by the deubiquitinase OTULIN [3, 4]. While M1-linked Ub chains constitute only ~0.5% of cellular Ub chains [5], they are essential for maintaining tissue and immune homoeostasis. LUBAC regulates innate immune pathways, particularly TNF signalling, NF-κB activation, and cell death, by conjugating non-degradative M1-linked Ub chains to substrates, prominently within the TNF receptor-1 (TNFR1) [1] complex, forming recruitment and activation scaffolds for signalling proteins, including the IκB kinase (IKK) complex [1].

Consistent with their important role in immune signalling, loss of LUBAC subunits or OTULIN in mice causes embryonic lethality [4, 6, 7] or inflammatory disease [8–11], while in humans, loss-of-function mutations in HOIP, HOIL-1, or SHARPIN, or in OTULIN cause the life-threatening autoinflammatory syndromes LUBAC deficiency or OTULIN-related autoinflammatory syndrome (ORAS), respectively [5, 8, 12–15]. Beyond inflammation, both syndromes are associated with metabolic abnormalities. HOIP and HOIL-1 mutations lead to amylopectinosis with polyglucosan body accumulation in muscle and heart [12, 13, 16], whereas OTULIN deficiency causes lipodystrophy, subcutaneous adipose tissue inflammation (panniculitis), and liver steatosis [5, 8, 15, 17]. In mice, hepatocyte-specific loss of OTULIN induces severe dysmetabolism, mTORC1 dysregulation, and hepatocellular carcinoma independently of TNFR1 [17, 18]. However, the mechanisms through which LUBAC and OTULIN regulate metabolism are largely unexplored.

Maintaining energy and metabolic homeostasis is vital for cellular function and survival and is achieved through coordinated molecular mechanisms that sense and respond to fluctuations in energy and nutrient availability. Central to this regulatory network is AMP-activated protein kinase (AMPK), a highly conserved Ser/Thr kinase activated by cellular energy depletion [19]. AMPK is a heterotrimer consisting of a catalytic α subunit and two regulatory subunits, β and γ. Canonical AMPK activation occurs when rising AMP:ATP or ADP:ATP ratios during energy stress promote AMP/ADP binding to the γ subunit, displacing ATP and inducing a conformational rearrangement that exposes Thr172 (T172) on the α subunit for phosphorylation [20–22]. T172 is phosphorylated by liver kinase B1 (LKB1), or in a nucleotide-independent manner by Ca^2+^/Calmodulin-dependent protein kinase 2 (CaMKK2), thereby activating AMPK [23]. Active AMPK phosphorylates numerous targets to orchestrate metabolic adaptation by inhibiting anabolic processes and promoting catabolic pathways [19]. Key downstream actions of AMPK include inhibition of fatty acid synthesis and activation of fatty acid oxidation via acetyl-CoA carboxylase 1 (ACC1) and ACC2, suppression of mammalian target of rapamycin complex 1 (mTORC1) through regulatory-associated protein of mTOR (RAPTOR) and tuberous sclerosis complex 2 (TSC2), and induction of autophagy through unc-51-like autophagy activating kinase 1 (ULK1) or mTORC1 inhibition [24, 25]. AMPK also modulates inflammatory signalling and shapes immune-metabolic responses in T cells and macrophages [26].

Due to its vital roles in energy homeostasis, fatty acid and cholesterol synthesis, mitochondrial biogenesis, autophagy, and insulin sensitivity, AMPK is an attractive therapeutic target in metabolic diseases and cancer [27]. While AMPK activation is well-known to be regulated by phosphorylation [28] and allosteric nucleotide binding, the contribution of ubiquitination, particularly chain type-specific ubiquitination, to AMPK regulation is poorly understood.

Here, we reveal unexpected and important roles of LUBAC and OTULIN in AMPK signalling and the response to energetic stress. We show that LUBAC and OTULIN control AMPK signalling across multiple cell types and tissues, at steady-state and in response to glucose starvation. Mechanistically, LUBAC can directly ubiquitinate AMPKα and β subunits and controls both the amplitude and threshold of AMPK activation. Strikingly, loss of HOIP renders both mammalian cells and *Drosophila* incapable of mounting a functional response to energetic stress, sensitising them to starvation-induced death. These findings reveal the first mechanism by which LUBAC and M1-linked Ub regulate metabolic signalling and establish a new level of regulation of AMPK by M1-linked ubiquitination.

## Results

### LUBAC and OTULIN form a complex with AMPK in cells

**Fig. 1:**
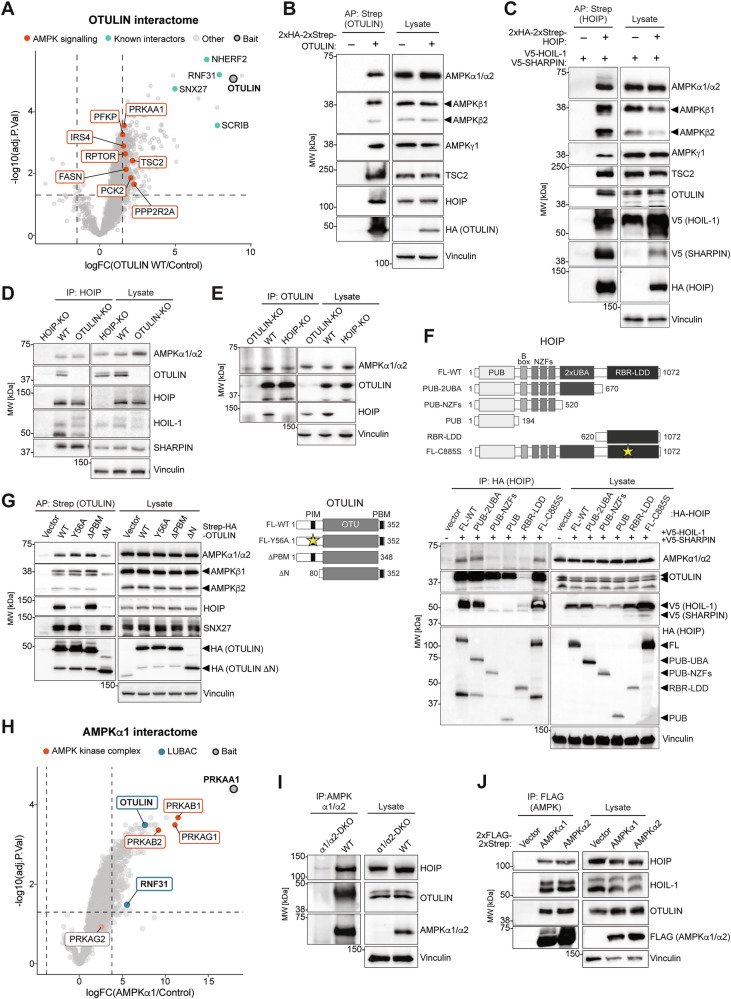
Proteomics identify LUBAC and OTULIN as interactors of AMPK. **A** Volcano plot of proteins from MS analysis of Strep-OTULIN affinity-purification (AP) from HEK293T cells versus vector control. Known OTULIN interactors (teal) and AMPK pathway proteins (orange) highlighted. False discovery rate (FDR) < 0.05; Fold-change (FC) cutoff 2.5. **B** Immunoblots of Strep-OTULIN AP from HEK293T cells transfected with 2xHA-2xStrep-OTULIN or vector. **C** Immunoblots of Strep-HOIP AP from HEK293T cells co-transfected with V5-HOIL-1, V5-SHARPIN, and either 2xHA-2xStrep-HOIP or vector. Immunoblots of endogenous HOIP (**D**) or OTULIN (**E**) immunoprecipitation (IP) from WT, HOIP-KO, and OTULIN-KO U2OS cells. **F** Top: Schematic representation of HOIP variants, including full-length wild-type (FL-WT), truncated mutants (PUB-UBA, PUB-NZFs, PUB, RBR-LDD), and the catalytically inactive mutant (FL-C885S). Bottom: Immunoblots of HA-tagged HOIP variants AP from HEK293T cells transfected with V5-HOIL-1, V5-SHARPIN, and 2xHA-2xStrep-HOIP or vector. **G** Left: Immunoblots of Strep-OTULIN AP from HEK293T cells transfected with 2xHA-2xStrep-OTULIN variants or vector. Right: Schematic of OTULIN WT, ΔPBM, Y56A, and ΔN mutants. **H** Volcano plot of proteins from MS analysis of Strep-AMPKα1 (PRKAA1) AP from HEK293T cells versus vector control. AMPK subunits (PRKAB1/2 and PRKAG1) (orange) and OTULIN and HOIP (RNF31) (blue) are highlighted. FDR < 0.05; FC cutoff 3.7. **I** Immunoblots of endogenous AMPKα1/2 IP from U2OS WT or AMPKα1/2 double-KO (DKO) cells. **J** Immunoblots of FLAG-AMPKα1 or α2 IP from HEK293T cells transfected with vector, 2xFLAG-2xStrep-AMPKα1, or α2. All blots are representative of three independent experiments.

We previously reported metabolic dysregulation in mice with hepatocyte-specific deletion of OTULIN [17], but how M1-linked Ub chains regulate metabolism remains unclear. To identify metabolic pathways regulated by M1-linked ubiquitination, we enriched Strep-tagged OTULIN from HEK293T cells and analysed the OTULIN interactome using mass spectrometry (MS)-based proteomics (Table S1). Interestingly, we identified multiple OTULIN interactors in the AMPK signalling pathway, including the catalytic AMPKα1 subunit (PRKAA1) and the AMPK substrates RAPTOR [29] and TSC2 [30] (Fig. 1A). These interactions were largely unaffected by deletion of OTULIN’s C-terminal PDZ domain-binding motif (PBM), which mediates interactions with PDZ domain-containing proteins such as SNX27 [31] (Fig. S1A–D). This shows that OTULIN binds AMPK pathway proteins independently of PDZ-mediated interactions, and suggests OTULIN may directly impact AMPK signalling.

We validated the interaction between OTULIN and AMPK by affinity purification of Strep-tagged OTULIN followed by immunoblotting. AMPKα1/2, β1, β2, and γ1, and the AMPK substrate TSC2, co-precipitated with OTULIN (Fig. 1B). Since OTULIN antagonises LUBAC signalling [3, 32], we hypothesised that LUBAC may also interact with AMPK. We expressed HOIP, HOIL-1, and SHARPIN in cells and affinity-purified LUBAC via Strep-tagged HOIP. Indeed, AMPKα1/2, β1, β2, and γ1 also co-precipitated with LUBAC (Fig. 1C), confirming an interaction between LUBAC and AMPK, suggesting a role for M1-linked ubiquitination in AMPK regulation.

We further examined the LUBAC-OTULIN-AMPK interaction by immunoprecipitating endogenous HOIP and OTULIN, which both co-precipitated endogenous AMPKα1/2 (Fig. 1D, E). Because OTULIN interacts directly with LUBAC by binding to HOIP’s PUB domain via its PUB-interacting motif (PIM) [33, 34], we tested whether HOIP and OTULIN can interact with AMPK independently. HOIP retained AMPKα1/2 binding in OTULIN knockout (KO) cells (Fig. 1D), whereas OTULIN co-precipitated less AMPKα1/2 from HOIP-KO cells (Fig. 1E), indicating that OTULIN interacts with AMPK partly through LUBAC.

To map the HOIP domains required for AMPK interaction, we analysed HOIP mutants. Full-length wild-type (WT) HOIP and HOIP lacking the RBR-LDD domain bound AMPK, whereas deleting both the RBR-LDD and double-UBA domains, or expressing the RBR-LDD domain alone, prevented AMPK binding (Fig. 1F), indicating that HOIP’s double-UBA domain is required for AMPK interaction. As HOIL-1 and SHARPIN bind HOIP via its double-UBA to form trimeric LUBAC [2, 35], this suggests that HOIL-1, SHARPIN, or full assembly of LUBAC mediates the AMPK interaction. For OTULIN, the N-terminal PIM-containing region was important for AMPK binding as the ΔN mutant bound less AMPKα1/2 and β1/2 than WT OTULIN (Fig. 1G), consistent with OTULIN binding AMPK through LUBAC (Fig. 1E). In contrast, the OTULIN^Y56A^ mutant, which reduces binding to HOIP [33, 34], bound AMPK to a similar extent as WT OTULIN (Fig. 1G), suggesting that the OTULIN N-terminus also makes direct contacts to AMPK.

We further investigated the LUBAC-OTULIN-AMPK complex by immunoprecipitating FLAG-tagged AMPKα1 and analysing its interactors by proteomics (Fig. S1E and Table S2). Both OTULIN and HOIP were significantly enriched in the AMPKα1 interactome along with AMPK β1, β2, and γ1 (Fig. 1H), with >100 interactors overlapping between the OTULIN^WT^ and AMPK interactomes (Fig. S1F). Immunoprecipitation of endogenous AMPKα1/2 confirmed interactions with HOIP and OTULIN (Fig. 1I). To test whether both catalytic AMPK isoforms interact with LUBAC and OTULIN, we precipitated FLAG-tagged AMPKα1 or α2. Both isoforms co-precipitated HOIP, HOIL-1, and OTULIN (Fig. 1J), showing that the M1-linked ubiquitination machinery interacts with AMPK complexes containing either catalytic subunit.

Together, our findings show that LUBAC, OTULIN, and AMPK form an endogenous complex in cells and that OTULIN interacts with AMPK mainly through LUBAC.

### LUBAC and OTULIN regulate AMPK signalling in response to energetic stress

Our identification of a LUBAC-OTULIN-AMPK complex prompted us to test whether LUBAC and OTULIN are important for AMPK signalling. We subjected cells to energetic stress by glucose starvation and analysed AMPK signalling by immunoblotting. In U2OS cells, siRNA-mediated knockdown of LUBAC (HOIP, HOIL-1, and SHARPIN) or of HOIP alone reduced the phosphorylation of AMPKα1/2 (T172) and its substrates ACC (S79) and RAPTOR (S792) in response to glucose starvation compared with control siRNA (Fig. S2A–C). We also observed a reduction in starvation-induced AMPK, ACC, and RAPTOR phosphorylation in U2OS HOIP-KO cells (Fig. S2D).

**Fig. 2:**
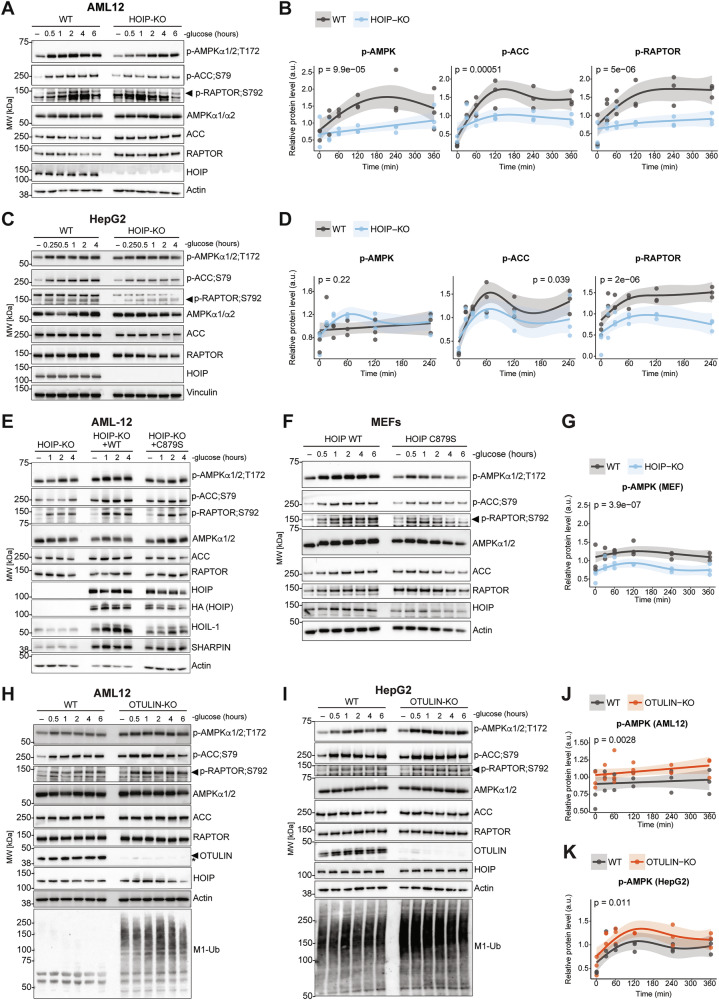
LUBAC and OTULIN regulate AMPK signalling after glucose starvation. **A** Immunoblots of AML12 WT and HOIP-KO cells starved in glucose-free medium for the indicated times. **B** Densitometric analysis of (**A**) from three independent experiments. Smoothed trendlines fitted by generalised additive model (GAM); *p*-value compares HOIP-KO to WT across all time points. **C** Immunoblots of HepG2 WT and HOIP-KO cells starved as in (**A**). **D** Densitometric analysis of (**C**), performed as in (**B**). **E** Immunoblots of AML12 HOIP-KO cells stably reconstituted with HA-HOIP-WT or catalytically inactive HA-HOIP-C879S, starved as in (**A**). **F** Immunoblots of WT or HOIP-C879S knock-in MEFs starved as in (**A**). **G** Densitometric analysis of (**F**), performed as in (**B**). **H** Immunoblots of AML12 WT and OTULIN-KO cells starved as in (**A**). **I** Immunoblots of HepG2 WT and OTULIN-KO cells starved as in (**A**). **J**, **K** Densitometric analysis of (**H**, **I**), respectively, performed as in (**B**). All blots are representative of three independent experiments.

Given AMPK’s vital role in hepatic metabolism [36], we tested whether LUBAC functions in AMPK signalling in the hepatocyte-derived cell lines AML12 and HepG2. Consistent with our U2OS data, HOIP deletion in AML12 and HepG2 cells significantly reduced the starvation-induced phosphorylation of AMPK, ACC, and RAPTOR (Fig. 2A–D). The milder effect on phospho-AMPK observed in the HepG2 cells may be due to adaption to permanent HOIP loss as siRNA-mediated LUBAC knockdown caused a more prominent reduction in AMPK phosphorylation (Fig. S2E). Across all timepoints (30 min to 6 h), HOIP loss consistently reduced—but did not complete block—AMPK pathway activation without overtly changing signalling kinetics (Fig. 2A–D).

To determine whether LUBAC’s ubiquitin ligase activity is important for AMPK signalling, we stably reintroduced either HOIP^WT^ or the catalytically inactive HOIP^C879S^ mutant into HOIP-deficient AML12 cells. Intriguingly, only HOIP^WT^ restored starvation-induced phosphorylation of AMPK, ACC, and RAPTOR (Fig. 2E). Consistently, HOIP^C879S^ knock-in MEFs [37] displayed significantly reduced AMPK phosphorylation and downstream signalling after glucose starvation compared with WT MEFs (Fig. 2F, G). Thus, LUBAC’s catalytic activity is required for robust AMPK pathway activation across multiple cell types following glucose starvation.

OTULIN antagonises LUBAC and restricts M1-linked Ub signalling in the NF-κB pathway [3, 8, 32], so we tested whether OTULIN also restricts AMPK signalling. OTULIN-KO AML12 and HepG2 cells displayed enhanced AMPK signalling with hyperphosphorylation of AMPK, ACC, and RAPTOR (Fig. 2H–K), while maintaining normal HOIP levels and accumulating M1-linked Ub, consistent with increased LUBAC activity (Fig. 2H, I). OTULIN knockdown in U2OS cells produced a similar, albeit milder, increase primarily in ACC and RAPTOR phosphorylation (Fig. S2F). These data mirror our observations in HOIP- and LUBAC-deficient cells and support a role for OTULIN as a negative regulator of AMPK signalling.

Together, our data demonstrate that LUBAC and OTULIN are bona fide regulators of AMPK signalling in response to energetic stress: LUBAC’s E3 ligase activity promotes AMPK activation, whereas OTULIN restricts it, highlighting a key role for M1-linked ubiquitination in controlling AMPK signalling.

### LUBAC and OTULIN control AMPK activation in liver and adipose tissue

**Fig. 3:**
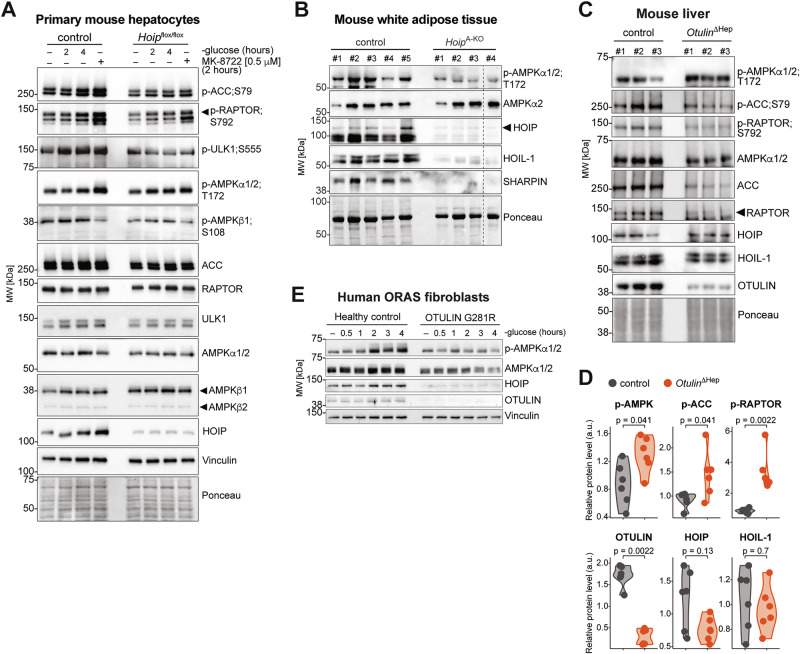
LUBAC and OTULIN regulate AMPK activation in liver and adipose tissue. **A** Immunoblots of primary hepatocytes from tamoxifen-treated CreERT2-*Hoip*^flox/flox^ mice or controls starved of glucose or treated with MK-8722. **B** Immunoblots of WAT lysates from four *Adipoq*Cre-*Hoip*^flox/flox^ (*Hoip*^A-KO^) mice and five controls. Dashed line indicates spliced blot. **C** Immunoblots of liver lysates from three 9-day-old control *Alb*Cre*-Otulin*^flox/flox^ (*Otulin*^ΔHep^) mice and three controls. **D** Densitometric analysis of (**C**) and Fig. S3F. Plots represent mean phosphoprotein intensity normalised to total protein (*n* = 6). Analysed by two-sided Mann-Whitney U test. **E** Immunoblots of fibroblasts derived from a homozygous OTULIN-G281R ORAS patient and a healthy control starved of glucose. **A**, **E** All blots are representative of three independent experiments.

We extended our investigation to CreERT2-*Hoip*^flox/flox^ mice, enabling tamoxifen-inducible deletion of *Hoip* (germline ablation of *Hoip* causes embryonic lethality [6]). Tamoxifen administration caused significant weight loss in *Hoip*^flox/flox^ mice (Fig. S3A–D), preventing longer-term studies in vivo. Instead, we isolated hepatocytes from livers of tamoxifen-treated *Hoip*^flox/flox^ mice (Figure S3A) and starved them of glucose ex vivo. Consistent with our findings in cell lines, loss of HOIP blunted AMPK signalling in primary hepatocytes, with reduced phosphorylation of ACC, RAPTOR, AMPKα1/2, AMPKβ1, and ULK1, compared with controls (Fig. 3A). Surprisingly, the HOIP-deficient hepatocytes were also less responsive to the small-molecule AMPK activator MK-8722 [38] (Fig. 3A), showing that LUBAC promotes AMPK signalling during glucose starvation and direct allosteric activation.

To investigate the impact of LUBAC deficiency on in situ AMPK signalling in metabolically active tissues, we crossed *Hoip*^flox/flox^ mice with *Adipoq*Cre-expressing mice, resulting in specific deletion of *Hoip* in adipocytes (*Hoip*^A-KO^ mice; Figure S3E). In subcutaneous white adipose tissue (WAT) from 22 to 23-week-old male *Hoip*^A-KO^ mice fed a normal diet, all LUBAC components were markedly reduced (Fig. 3B). Strikingly, even under fed, non-starved conditions, AMPK T172 phosphorylation was reduced in *Hoip*^A-KO^ WAT compared with controls (Fig. 3B), indicating that LUBAC also supports baseline AMPK activation in WAT under physiological conditions.

We next assessed whether OTULIN regulates AMPK in situ. Mice with hepatocyte-specific deletion of OTULIN (*Otulin*^ΔHep^) display neonatal steatosis, reduced hepatic glycogen, and mTORC1 dysregulation [17]. In livers from 9-day-old *Otulin*^ΔHep^ mice, AMPK T172 phosphorylation was markedly elevated despite slightly reduced AMPKα1/2 levels (Figs. 3C, D and S3F), even without starvation, mirroring the baseline defects HOIP-deficient WAT. ACC and RAPTOR protein levels were markedly reduced (Fig. 3C), resembling mTORC1 alterations in *Otulin*^ΔHep^ mice [17]. Importantly, after normalising protein levels, we observed significant increases in ACC and RAPTOR phosphorylation (Fig. 3D), showing that OTULIN limits baseline AMPK signalling in situ. Thus, LUBAC and OTULIN control AMPK pathway activation in metabolically active tissues, even in the absence of energetic stress, highlighting a role for M1-linked ubiquitination in steady-state AMPK regulation.

Given these effects in mice, we asked whether AMPK signalling is dysregulated in human disease with aberrant M1-linked ubiquitination. We analysed human skin fibroblasts derived from an ORAS patient carrying a homozygous OTULIN^G281R^ mutation [5]. These fibroblasts phenocopy LUBAC-deficient cells, as the loss of OTULIN is accompanied by substantial reduction in HOIP levels [5] (Fig. 3E), an effect only observed in certain cells, such as fibroblasts, B and T cells [5, 8]. Strikingly, AMPK T172 phosphorylation was strongly reduced upon glucose starvation in OTULIN^G281R^ fibroblasts compared with healthy controls (Fig. 3E). This demonstrates that M1-linked Ub signalling is important for proper AMPK regulation in primary human cells and suggests that dysregulated AMPK signalling may contribute to cellular malfunctions in ORAS and LUBAC deficiency.

### AMPKα and β subunits are substrates of LUBAC ubiquitination

**Fig. 4:**
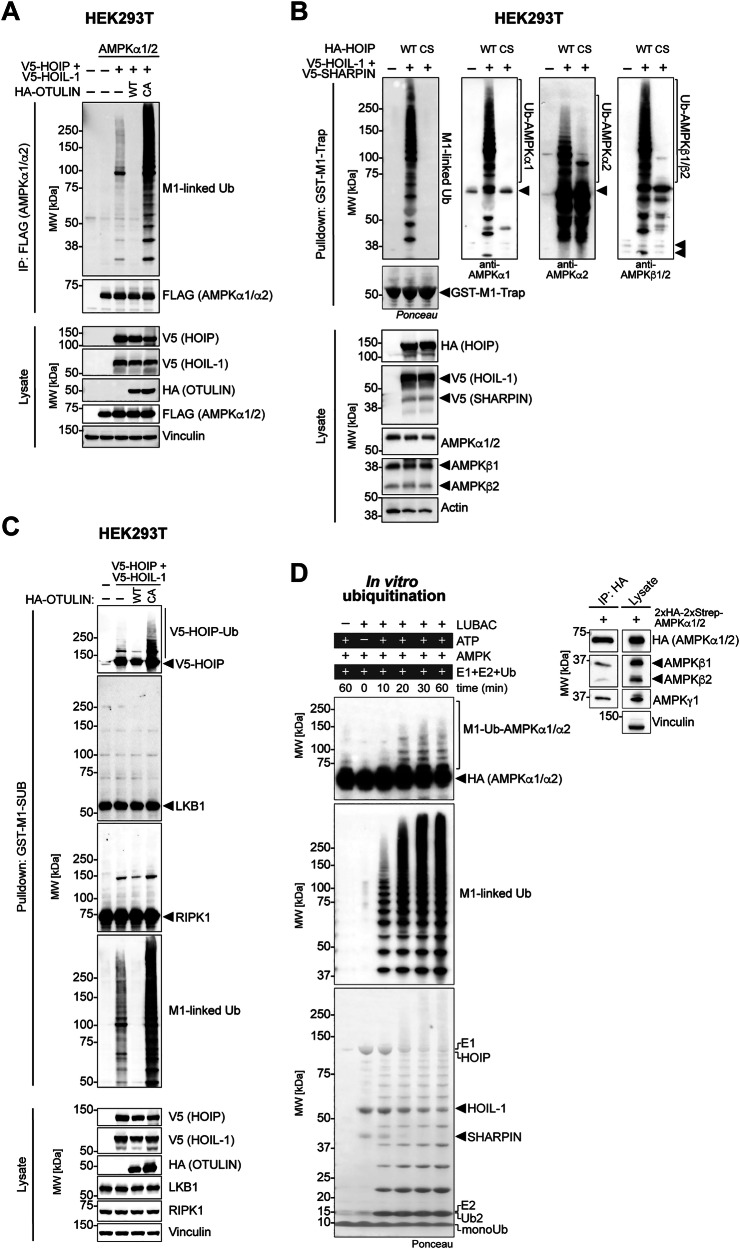
AMPKα and β subunits are substrates of LUBAC ubiquitination. **A** Immunoblots of FLAG-AMPKα1/2 IP from HEK293T cells co-transfected with LUBAC (HOIL-1 and HOIP), OTULIN-WT, catalytically inactive OTULIN-C129A (CA), or vector. **B**, **C** Immunoblots of M1-linked Ub conjugates purified from HEK293T cells transfected with LUBAC (HOIL-1, SHARPIN, and HOIP-WT or HOIP-C885S (CS)) (**B**) or LUBAC (HOIL-1 and HOIP) and OTULIN-WT or OTULIN-C129A (CA) (**C**) using recombinant GST-M1-Trap or GST-M1-SUB. **D** Immunoblots of in vitro ubiquitination assay (left) of HA-AMPKα1/2 IP from HEK293T cells (right) using recombinant E1, E2, LUBAC (HOIP, HOIL-1, and SHARPIN), and ubiquitin. All blots are representative of three (**A**, **B**, **D**) or two (**C**) independent experiments.

We next tested whether AMPK associates with M1-linked Ub and whether LUBAC can ubiquitinate AMPK. We expressed FLAG-tagged AMPKα1/2 together with HOIP, HOIL-1 and OTULIN in HEK293T cells and immunoprecipitated the AMPKα1/2 complexes. When co-expressed with HOIP and HOIL-1, AMPKα1/2 co-precipitated a clear smear of M1-linked Ub chains (Fig. 4A), indicating that LUBAC forms M1-linked Ub chains on proteins within or associated with the AMPK complex. These chains were completely removed by co-expression of OTULIN^WT^, whereas they accumulated when co-expressed with catalytically inactive OTULIN^C129A^, which protects and stabilises M1-linked chains [3, 32] (Fig. 4A, compare lane 3 with lanes 4 and 5). These data show that M1-linked Ub chains can be found in AMPK complexes.

To enrich M1-linked Ub, we used engineered binders [39], either our M1-Trap (OTULIN^80-348;C129A^) or the M1-SUB derived from NEMO’s UBAN domain [32], both of which bind M1-linked Ub with high affinity and specificity [3, 40]. Notably, the M1-Trap lacks OTULIN’s N-terminus and therefore does not interact with AMPK directly (Figs. 1G and S4A). To test whether LUBAC can ubiquitinate AMPK, we expressed LUBAC containing either HOIP^WT^ or the catalytically inactive HOIP^C885S^ mutant and enriched M1-linked Ub chains using GST-M1-Trap. LUBAC containing HOIP^WT^ induced robust ubiquitination of endogenous AMPKα1, α2, and β1/2, seen as high-molecular-weight smears, whereas HOIP^C885S^ failed to do so (Fig. 4B). LUBAC also autoubiquitinated HOIP but did not modify LKB1 or RIPK1 (LUBAC substrate in TNF signalling [1, 9]), even in the presence of OTULIN^C129A^ to stabilise the M1-linked chains (Figs. 4C and S4B). Glucose starvation did not further increase AMPK ubiquitination (Fig. S4B), likely due to saturation from ectopic LUBAC expression.

Finally, an in vitro ubiquitination assay using purified AMPK and recombinant LUBAC showed clear, LUBAC-dependent ubiquitination of AMPKα1/2 (Fig. 4D), demonstrating that LUBAC can ubiquitinate AMPK directly. These findings identify AMPK as a direct LUBAC substrate and provide a potential mechanism for M1-linked Ub-dependent regulation of AMPK signalling.

### LUBAC regulates the kinase activity and activation threshold of AMPK

**Fig. 5:**
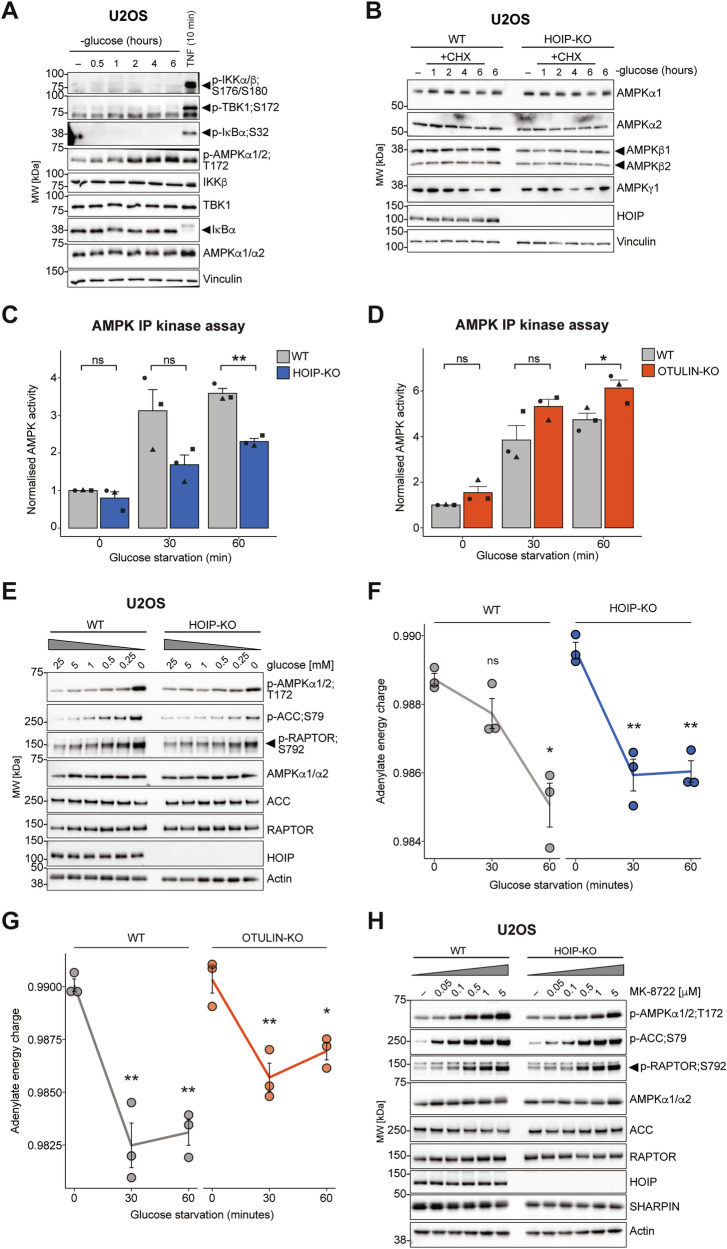
LUBAC controls the kinase activity and activation threshold of AMPK. **A** Immunoblots of U2OS WT cells starved of glucose or treated with TNF (10 ng/mL, 10 min). **B** Immunoblots of U2OS WT and HOIP-KO cells starved of glucose and co-treated with cycloheximide (CHX). AMPK IP kinase assays with ^33^P-labelled ATP from U2OS WT and HOIP-KO (**C**) or HepG2 WT and OTULIN-KO (**D**) cells starved of glucose as indicated. Bars represent mean ± SEM (*n* = 3). Analysed by two-sided Student’s *t*-test. **E** Immunoblots of U2OS WT and HOIP-KO cells in decreasing glucose concentrations for 60 min. Adenylate energy charge (AEC) determined by LC-MS/MS in glucose-starved U2OS WT and HOIP-KO (**F**) or HepG2 WT and OTULIN-KO (**G**) cells. Plot shows mean ± SEM (*n* = 3). Analysed by two-sided Student’s *t*-test. **H** Immunoblots of U2OS WT and HOIP-KO cells treated with increasing MK-8722 concentrations for 60 min. All blots are representative of three independent experiments.

Given that LUBAC can ubiquitinate AMPK, we investigated how LUBAC controls AMPK signalling. LUBAC regulates TNF-induced IKK and NF-κB activation [1], which can influence AMPK signalling [41]. However, during glucose starvation, we observed robust AMPK phosphorylation without detectable phosphorylation of IKKα/β, TBK1, or IκBα, and no IκBα degradation (Fig. 5A). Neutralising TNF did not affect AMPK signalling but blocked TNF-induced IκBα degradation and NF-κB p65 phosphorylation (Fig. S5A, B). Moreover, no caspase-3 cleavage was detected in HOIP-KO cells for up to 6 h of glucose starvation (Fig. S5C, D), indicating that impaired AMPK signalling is not a consequence of cell death. Together, this shows that AMPK activation during glucose starvation occurs independently of autocrine TNF and IKK signalling, indicating that LUBAC regulates AMPK through mechanisms distinct from its established roles in TNF signalling, IKK activation, and cell death.

AMPK ubiquitination has mainly been linked to regulation of its stability [42–44], but steady-state levels of AMPKα1, α2, β1, β2, or γ1, as well as mTOR-related proteins that could influence AMPK, were unchanged in WT and HOIP-KO cells (Figs. 5B and S5E). Cycloheximide chase experiments also showed no stability differences during glucose starvation (Fig. 5B). Thus, LUBAC regulates AMPK signalling through non-degradative mechanisms, in line with its canonical function [1].

To determine whether LUBAC affects AMPK kinase activity, we performed quantitative IP-coupled kinase assays. Endogenous AMPK was immunoprecipitated and its activity measured using a synthetic substrate peptide and radiolabelled ATP [45]. Interestingly, AMPK activity after glucose starvation was reduced to ~50–60% in HOIP-KO cells or following LUBAC knockdown (Figs. 5C and S5F). Conversely, AMPK activity from OTULIN-KO cells increased by ~20–25% relative to WT (Fig. 5D). These results show that LUBAC and OTULIN regulate AMPK’s kinase activity, consistent with the altered signalling in HOIP- and OTULIN-deficient cells (Fig. 2).

We noted that LUBAC deficiency impacted AMPK activity as early as 30 minutes after glucose withdrawal (Figs. 5C and S5F). We therefore investigated whether LUBAC influences AMPK’s sensitivity to decreasing glucose or energy levels by exposing WT and HOIP-KO cells to graded glucose concentrations (25–0 mM). Loss of HOIP reduced AMPK’s responsiveness to glucose depletion. In WT cells, AMPK, ACC, and RAPTOR phosphorylation appeared at ~1 mM glucose, whereas HOIP-KO cells required ~0.25 mM glucose or less (Fig. 5E). A similar shift in glucose sensitivity was observed after LUBAC knockdown (Fig. S5G). Thus, HOIP deficiency increases the activation threshold for AMPK during energetic stress.

AMPK is canonically activated by increased AMP:ATP or ADP:ATP ratios, but it can also directly sense glucose [46]. To determine whether loss of HOIP or OTULIN affects cellular energy balance, we quantified adenine nucleotide levels during glucose starvation using mass spectrometry and calculated the adenylate energy charge (AEC), an index of cellular energy status. In WT cells, AEC dropped significantly within 30–60 min of glucose starvation (Fig. 5F, G), reflecting reduced energy levels and rising AMP/ADP:ATP ratios. HOIP- or OTULIN-KO cells showed similar decreases in AEC (Fig. 5F, G), demonstrating energy depletion comparable to WT cells. Thus, LUBAC and OTULIN act downstream of changes in adenine nucleotide ratios, and the impaired AMPK activation in HOIP-deficient cells is not due to lower AMP or ADP levels in these cells during starvation.

We further tested whether LUBAC influences nucleotide-independent AMPK activation. HOIP-deficient primary hepatocytes were less sensitive to the allosteric AMPK activator MK-8722 (Fig. 3A), which stabilises phosphorylated T172 at the ADaM site independently of nucleotides [47, 48]. When we treated cells with MK-8722, WT cells showed robust AMPK, ACC, and RAPTOR phosphorylation at ≤0.05 μM MK-8722, whereas HOIP-KO cells required ≥0.5 μM for a comparable response (Fig. 5H). This demonstrates that LUBAC contributes to full AMPK activation in response to both increased AMP/ADP levels and direct allosteric activation.

Collectively, our data show that LUBAC and OTULIN regulate AMPK kinase activity without affecting adenine nucleotide ratios during glucose starvation, indicating that M1-linked ubiquitination regulates AMPK downstream of energy-sensing. Moreover, LUBAC controls both the amplitude and activation threshold of AMPK in response to glucose starvation and activation by MK-8722.

### Phosphoproteomics reveal LUBAC-dependent signalling during energetic stress

**Fig. 6:**
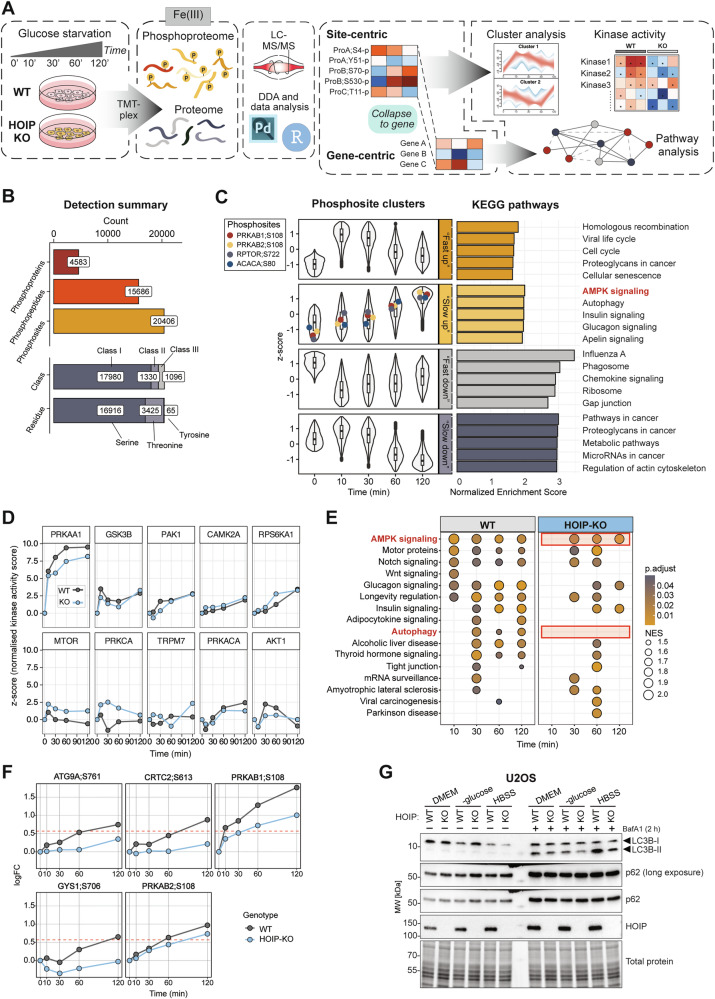
Phosphoproteomics reveal the LUBAC-dependent signalling network after glucose starvation. **A** Schematic of LC-MS/MS-based phosphoproteomics in U2OS WT and HOIP-KO cells glucose-starved for 0–120 min (*n* = 3 per timepoint). **B** Top: Bar plots showing total numbers of phosphoproteins, phosphopeptides, and phosphosites in the dataset. Bottom: Numbers of Class I, II, and III phosphosites with localisation probabilities >75%, 50–75%, and <50%, respectively, and total numbers of phosphosites on serine, threonine, and tyrosine residues. **C** Left: Violin plots showing four fuzzy c-means cluster profiles of phosphosite z-scores over time in WT cells. Clusters were named by phosphorylation dynamics. AMPKβ1/2;S108, RAPTOR;S722, and ACC1;S80 are included in the ‘Slow up’ cluster. Right: Bar plots showing the top five KEGG pathways significantly enriched in each cluster (GSEA, *p* < 0.005), with ‘AMPK signalling’ highlighted in the ‘Slow up’ cluster. **D** Line plots of KSEA kinase z-scores for the 10 most active kinases in the ‘Slow up’ cluster in WT (grey) and HOIP-KO (blue) cells. **E** Dot plot showing KEGG pathway enrichment in WT and HOIP-KO cells during starvation. Size indicates NES; colour indicates FDR-adjusted p-value. **F** Log2FC over untreated for select phosphosites in WT (grey) and HOIP-KO (blue) cells. Red line: level for statistically significant up-regulation. **G** Immunoblots of U2OS WT and HOIP-KO cells starved in glucose-free medium or HBSS and treated with BafA1 (2 h). Blots are representative of three independent experiments.

Energetic stress, such as starvation, triggers a range of adaptive processes in cells, including AMPK pathway activation. To investigate the broader role of M1-linked Ub signalling during energetic stress, we performed an MS-based phosphoproteomic analysis of glucose-starved U2OS WT and HOIP-KO cells (Fig. 6A). We identified >20,000 phosphorylation sites (phosphosites) across >4500 proteins (Fig. 6B). Glucose starvation induced significant changes in the phosphorylation of hundreds of sites (Fig. S6A, B and Table S3), but the overall phosphoproteomic landscape remained similar between WT and HOIP-KO cells (Fig. S6C), indicating that HOIP deficiency perturbs specific signalling events rather than causing global signalling disruption.

We grouped phosphosites based on their dynamics using fuzzy c-means clustering (Fig. 6A, C). The autophosphorylation sites of AMPKβ1/2 (PRKAB1/2; S108) and the canonical AMPK sites on ACC (S80) and RAPTOR (S722) were part of the ‘*Slow up*’ cluster (Fig. 6C; left). KEGG pathway enrichment confirmed that AMPK signalling was strongly represented in this cluster (Fig. 6C; right, Table S4). Kinase-Substrate Enrichment Analysis (KSEA) identified AMPKα1 (PRKAA1) as the most active kinase in the cluster in both WT and HOIP-KO cells, alongside 9 other kinases, including GSK3β, S6K1, and CamK2α (Figs. 6D and S6D). Notably, AMPKα1 was the kinase most affected by HOIP loss with consistently lower activity in HOIP-KO cells (Figs. 6D and S6D). Conversely, mTOR activity—and that of its targets S6K1 and PKCα —was elevated in HOIP-KO cells (Figs. 6D and S6D), consistent with reduced AMPK activation. Together, these phosphoproteomic data confirm, on an unbiased systems-wide scale, that AMPK signalling is impaired in HOIP-KO cells during glucose starvation and that LUBAC specifically regulates AMPK signalling during energetic stress.

To focus our analysis of the cellular processes controlled by LUBAC during energetic stress, we collapsed the phosphosites in the ‘*Slow up*’ cluster into their corresponding genes for functional annotation (Fig. 6A). KEGG analysis revealed that AMPK signalling was the strongest and earliest response in WT cells, but was reduced and delayed in HOIP-KO cells (Fig. 6E and Table S5), consistent with our KSEA results. HOIP deficiency broadly caused impairment, delay, and disrupted coordination of starvation-responsive pathways (Fig. 6E), affecting early pathways such as Wnt signalling, consistent with reported M1-linked Ub functions [4], as well as insulin, adipokine, and longevity signalling in the later response (Fig. 6E and Table S5).

We also observed markedly impaired autophagy in HOIP-KO cells (Fig. 6E), consistent with reduced activation of AMPK. Closer inspection of autophagy- and AMPK-related phosphosites within this cluster revealed several sites with reduced phosphorylation in HOIP-KO cells, including ATG9A (S761), CRTC2 (S613), AMPKβ1/2 (S108), and GYS1 (S706) (Fig. 6F). These changes indicate that the phosphorylation-dependent, AMPK-mediated autophagic response to glucose starvation is compromised in the absence of HOIP. Consistently, HOIP-KO cells exhibited impaired autophagy upon glucose withdrawal or HBSS starvation (Fig. 6G). LC3B-I to LC3B-II conversion and p62 degradation were assessed with or without Bafilomycin A1 (BafA1), which blocks lysosomal acidification and thereby prevents autophagosome degradation. Starvation-induced p62 degradation (without BafA1) was impaired in HOIP-KO cells, and BafA1-mediated accumulation of LC3B-II and p62 was reduced relative to WT cells, indicating defective autophagic flux (Fig. 6G). These findings suggest that LUBAC-deficient cells have impaired phosphorylation-dependent induction of autophagy.

In summary, our phosphoproteomic analysis shows that LUBAC controls a defined subset of kinase signalling events, primarily AMPK signalling, during energetic stress and identifies LUBAC as a regulator of starvation-induced autophagic signalling.

### LUBAC regulates bioenergetic metabolism and survival in response to energetic stress

**Fig. 7:**
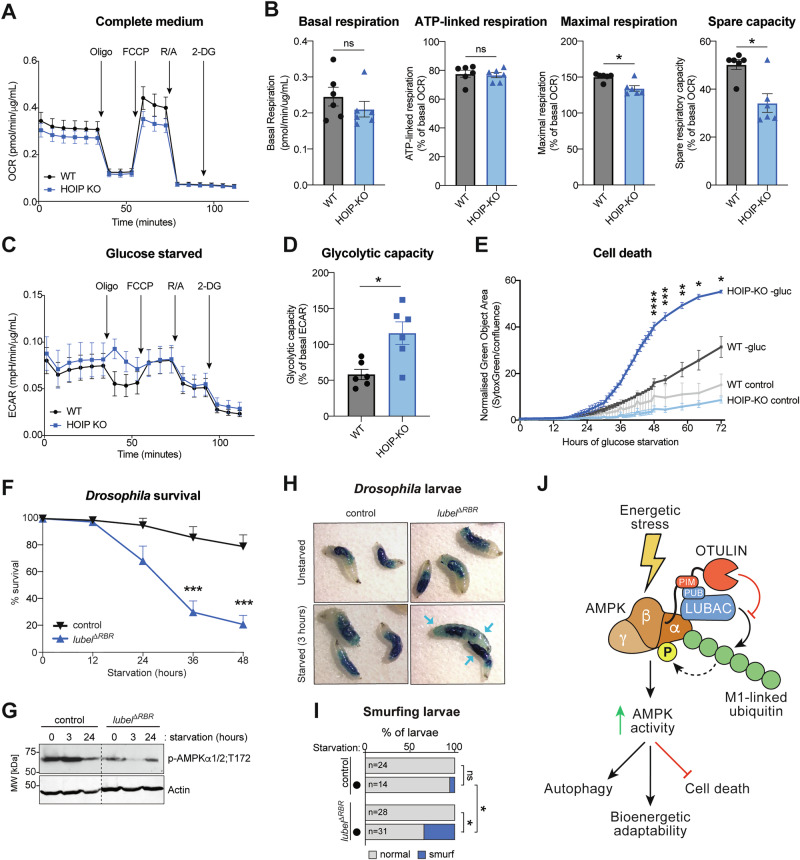
LUBAC regulates bioenergetic metabolism and survival in response to starvation. **A** Oxygen consumption rate (OCR) in U2OS WT (black) and HOIP-KO (blue) cells measured with the indicated perturbations. **B** Bar plots showing basal, ATP-linked, and maximal respiration, and spare respiratory capacity. Bars show mean ± SEM (*n* = 6). Analysed by two-sided Mann-Whitney U test. **C** Extracellular acidification rate (ECAR) in U2OS WT and HOIP-KO cells in glucose-free medium with the indicated perturbations. **D** Glycolytic capacity (% basal ECAR) in U2OS WT and HOIP-KO cells. Bars show mean ± SEM (*n* = 6). Analysed by two-sided Mann-Whitney U test. **E** Cell death measured over 72 h in AML12 WT and HOIP-KO cells in complete or glucose-starved conditions. Death was quantified as Sytox Green normalised to confluence. Lines show mean ± SEM (*n* = 3). Analysed by two-sided Student’s *t*-test. **F** Survival of control and *lubel*^ΔRBR^ *Drosophila* upon starvation. Data represent mean ± SEM (*n* ≥ 3). Analysed by two-way ANOVA with Tukey’s post-hoc test. **G** Immunoblots control and *lubel*^ΔRBR^ *Drosophila* starved for 3–24 h. Dashed line indicates spliced blots. Blots are representative of three independent experiments. **H** Images of third instar control and *lubel*^ΔRBR^ *Drosophila* larvae on Brilliant Blue-supplemented control or starvation medium. Arrows indicate ‘smurf’ phenotype. **I** Bar plots showing ‘smurf’ proportion in control and *lubel*^ΔRBR^ *Drosophila* larvae. Analysed by Fisher’s exact test with FDR correction. **J** Model of LUBAC- and OTULIN-mediated regulation of AMPK. LUBAC and OTULIN form a complex with AMPK, with OTULIN recruited primarily through its interaction with HOIP and additional interactions with AMPK via its N-terminal part. LUBAC can directly M1-ubiquitinate AMPKα and β subunits, and its ubiquitin ligase activity promotes AMPKα1/2 T172 phosphorylation and activation during energetic stress. OTULIN counteracts this and restricts AMPK activation. Through this dynamic, LUBAC enhances the energetic stress response by supporting AMPK activation, autophagy, and bioenergetic adaptability, thereby protecting cells from starvation-induced death.

Because LUBAC is required for robust AMPK activation and induction of autophagy during starvation, we examined whether LUBAC controls cellular metabolism and energetics. Bioenergetic profiling of WT and HOIP-KO cells in complete and glucose-free medium (Fig. S7A, B) showed that HOIP-KO cells had normal basal oxygen consumption rate (OCR) and responded to mitochondrial perturbations similar to WT cells, indicating no overt defects their baseline respiration (Fig. 7A). Basal and ATP-linked respiration were also unchanged (Fig. 7B), showing that baseline mitochondrial function and energy demand are similar. However, maximal respiration and spare respiratory capacity were reduced in HOIP-KO cells (Fig. 7B), indicating that they operate closer to their maximal bioenergetic limit and are less able to adapt to energetic stress. This decrease in spare capacity is consistent with impaired AMPK signalling, which normally promotes mitochondrial function and stress resilience [49].

To assess metabolic adaptation, we measured extracellular acidification rate (ECAR) after shifting cells to glucose-free medium (Fig. 7C). WT cells displayed a glycolytic capacity of ~55% following oligomycin treatment, indicating that glycolysis was already near maximal during starvation (Fig. 7C, D). In contrast, HOIP-KO cells showed a glycolytic capacity of ~115%, revealing that glycolysis remained submaximal in glucose-free medium and could still increase when mitochondrial ATP synthesis was inhibited (Fig. 7C, D). This failure to fully engage glycolysis suggests that HOIP-KO cells fail to execute a full glycolytic shift during starvation, consistent with impaired AMPK activation [50, 51]. Accordingly, HOIP-KO cells were significantly more sensitive to starvation-induced cell death after 24–72 h of glucose deprivation (Figs. 7E and S7C). These findings show that HOIP-deficient cells have compromised bioenergetic adaptability, rendering them more vulnerable to starvation and energetic stress.

To determine whether LUBAC also supports starvation resistance in vivo, we investigated the starvation response in *Drosophila melanogaster*, where both AMPK [52] and LUBAC (Lubel) [53, 54] are conserved. Control flies and flies with genetic deletion of Lubel’s catalytic RBR domain (*lubel*^ΔRBR^ flies) were starved for 48 hours. Strikingly, loss of Lubel activity caused a dramatic survival defect, with fewer than 25% surviving after 48 h (Fig. 7F), correlating with impaired AMPK T172 phosphorylation in the *lubel*^ΔRBR^ flies (Fig. 7G). We further assessed the acute starvation response in *lubel*^ΔRBR^ larvae using the ‘smurf’ assay, which reports intestinal barrier failure due to cell death [55]. Consistent with the reduced adult survival, after 3 h of starvation, there was a significantly higher proportion of ‘smurfs’ among *lubel*^ΔRBR^ larvae than controls (Fig. 7H, I), indicating that Lubel activity protects against starvation-induced cell death in larvae, similar to cultured cells.

In conclusion, our findings demonstrate that M1-linked Ub signalling supports an effective bioenergetic response to starvation and promotes AMPK activation and survival during energetic stress in both mammalian cells and *Drosophila*.

## Discussion

Our work identifies LUBAC and OTULIN as central regulators of AMPK signalling and the cellular response to energetic stress (Fig. 7J), establishing the first metabolic pathway governed by M1-linked ubiquitination. LUBAC’s catalytic activity is required for full AMPK activation, whereas OTULIN counteracts this by removing M1-linked Ub chains. Thus, M1-linked Ub signalling tunes both the amplitude and sensitivity of AMPK activation. Loss of LUBAC compromises metabolic adaptability, autophagy, and survival during starvation, and these defects are conserved in *Drosophila*, highlighting the physiological relevance of the LUBAC-AMPK signalling axis. Consistent with our observations, HOIP-deficient tumours in mice show reduced glycolytic activity [56], supporting our notion and further linking LUBAC to metabolic control. Together, our findings extend the functional scope of the M1-linked Ub machinery beyond inflammation and position LUBAC and OTULIN as key modulators of AMPK-driven stress responses.

Although LUBAC and OTULIN are central to control of TNF-induced IKK and NF-κB signalling, our data indicate that their role in AMPK signalling is independent of TNF, IKK, or NF-κB pathways. Glucose starvation did not induce IKK activation, and TNF blockade had no impact on AMPK signalling, clearly indicating that LUBAC’s function in AMPK signalling is distinct from its canonical immune signalling roles. The increased sensitivity of HOIP-deficient cells to prolonged starvation reflects an inability to mount an effective AMPK-driven stress response rather than secondary effects of early cell death, as no caspase activation was detectable during the first 6 h of starvation, showing that reduced AMPK signalling is not a consequence of cell death. The mechanisms of late-onset, starvation-induced cell death in LUBAC-deficient cells remain to be defined.

Mechanistically, HOIP loss leads to reduced AMPK activation with lower T172 phosphorylation and diminished phosphorylation of downstream substrates ACC and RAPTOR, although signalling is not completely blocked. Conversely, OTULIN deficiency causes moderate AMPK hypersignalling. These opposing effects mirror LUBAC’s and OTULIN’s roles in immune signalling, where M1-linked chains fine-tune, rather than are essential for, IKK/NF-κB activation and cell fate decisions [1, 57]. OTULIN is not the only deubiquitinase counteracting LUBAC. The SPATA2-CYLD complex also limits LUBAC signalling at immune receptors [58–60], raising the possibility that additional DUBs, including CYLD, might modulate AMPK. Overall, our findings suggest that in AMPK signalling, as in the IKK/NF-κB pathway, LUBAC and OTULIN act as modulators that shape signalling output and cellular outcomes rather than being essential for kinase activation.

We further show that LUBAC’s ubiquitin ligase activity (i.e. HOIP’s catalytic cysteine) is required for full AMPK activation. LUBAC can M1-ubiquitinate endogenous AMPK subunits in cells when overexpressed and, crucially, directly ubiquitinate purified AMPK in vitro, establishing AMPK as a bona fide LUBAC substrate. Starvation did not further enhance AMPK ubiquitination when LUBAC was overexpressed, likely due to saturation, but starvation induces rapid accumulation of M1-linked Ub in *Drosophila* [54], suggesting that Lubel is activated during energetic stress to modify signalling proteins. In this context, it is noteworthy that ATG13, an autophagy regulator downstream of AMPK, has also been reported as an LUBAC substrate [61], further linking M1-linked ubiquitin to AMPK-autophagy signalling. Our findings do not exclude the possibility that additional LUBAC substrates exist within the AMPK pathway, analogous to TNF signalling where multiple substrates (e.g. RIPK1, TNFR1, TRADD, and NEMO) are ubiquitinated by LUBAC [1]. Identifying such substrates remains an open and important question.

Intriguingly, our data suggest that AMPK may be a ubiquitin-activated kinase, analogous to IKK or TAK1. In the IKK pathway, M1-linked Ub chains recruit and activate the IKK complex via NEMO’s UBAN domain [40]. By analogy, M1-linked Ub, potentially conjugated to AMPK, could promote AMPK activation by enhancing LKB1 engagement, protecting T172 from phosphatases, or altering AMPK localisation or complex composition. Defining how M1-linked ubiquitin supports AMPK activation will be a major direction for future work.

In addition to severe TNF-driven autoinflammation, LUBAC deficiency and ORAS are associated with poorly understood abnormalities in glycogen and lipid metabolism, including glycogen storage disease (amylopectinosis), lipodystrophy, subcutaneous adipose tissue inflammation, and liver steatosis [5, 8, 12, 13, 15–17]. Given that both HOIP and OTULIN deficiency alter AMPK signalling in cells, tissues, and animal models, our findings suggest that AMPK dysregulation may contribute to the metabolic and clinical features of these disorders. Consistent with this, fibroblasts derived from an OTULIN^G281R^ ORAS patient display pronounced AMPK dysregulation. How altered AMPK signalling might contribute to metabolic defects or inflammatory pathology in LUBAC deficiency and ORAS remains an important area for future investigation.

In conclusion, our work conceptually expands the biological role of LUBAC and M1-linked Ub to include control of metabolic signalling and identifies atypical ubiquitination as a previously unrecognised layer of AMPK regulation.

## Methods

### Reagents and resources table

A list of key reagents and resources used in this study can be found in Table 1.

**Table 1:** Reagents and resources table.

Reagent or resource	Source	Identifier
**Antibodies:**		
Mouse monoclonal anti-SNX27 [1C6]	Abcam	Cat#ab77799
Rabbit monoclonal anti-AMPKγ1 [Y308]	Abcam	Cat#ab32508
Rabbit monoclonal anti-HOIL-1/RBCK1 [EPR28157-53]	Abcam	Cat#ab309104
Rabbit monoclonal anti-HOIP/RNF31 [EPR27947-265]	Abcam	Cat#ab315162
Rabbit polyclonal anti-β Actin	Abcam	Cat#ab8226
Rabbit polyclonal anti-RNF31/HOIP	Abcam	Cat#ab46322
Mouse monoclonal anti-V5-Tag (SV5-Pk1)	Bio-Rad	Cat#MCA1360GA
Mouse monoclonal anti-AMPKα1/2 (F6)	Cell Signaling Technology	Cat#2793
Mouse monoclonal anti-IκBα (L35A5)	Cell Signaling Technology	Cat#4814
Rabbit monoclonal anti-acetyl-CoA carboxylase (C83B10)	Cell Signaling Technology	Cat#3676
Rabbit monoclonal anti-AMPKβ1/2 (57C12)	Cell Signaling Technology	Cat#4150
Rabbit monoclonal anti-phospho-IκBα (Ser32) (14D4)	Cell Signaling Technology	Cat#2859
Rabbit monoclonal anti-HA-tag	Cell Signaling Technology	Cat#3724
Rabbit monoclonal anti-IKKβ (2C8)	Cell Signaling Technology	Cat#2370
Rabbit monoclonal anti-LKB1 (27D10)	Cell Signaling Technology	Cat#3050
Rabbit monoclonal anti-NF-κB p65 (D14E12) XP®	Cell Signaling Technology	Cat#8242
Rabbit monoclonal anti-OTULIN (E3B8L)	Cell Signaling Technology	Cat#74125
Rabbit monoclonal anti-phospho-IKKα/β (Ser176/180) (16A6)	Cell Signaling Technology	Cat#2697
Rabbit monoclonal anti-phospho-NF-κB p65 (Ser536) (93H1)	Cell Signaling Technology	Cat#3033
Rabbit monoclonal anti-RAPTOR (24C12)	Cell Signaling Technology	Cat#2280
Rabbit monoclonal anti-RIPK1 (D94C12) XP®	Cell Signaling Technology	Cat#3493
Rabbit monoclonal anti-TBK1/NAK (D1B4)	Cell Signaling Technology	Cat#3504
Rabbit monoclonal anti-Tuberin/TSC2 (D57A9)	Cell Signaling Technology	Cat#3990
Rabbit monoclonal anti-ULK1 (D8H5)	Cell Signaling Technology	Cat#8054
Rabbit monoclonal phospho-TBK1/NAK (Ser172) (D52C2) XP®	Cell Signaling Technology	Cat#5483
Rabbit monoclonal phospho-ULK1 (Ser757) (D7O6U)	Cell Signaling Technology	Cat#14202
Rabbit polyclonal anti-AMPKα1	Cell Signaling Technology	Cat#2795
Rabbit polyclonal anti-AMPKα2	Cell Signaling Technology	Cat#2757
Rabbit polyclonal anti-OTULIN	Cell Signaling Technology	Cat#14127
Rabbit polyclonal anti-phospho-Acetyl-CoA Carboxylase (Ser79)	Cell Signaling Technology	Cat#3661
Rabbit polyclonal anti-phospho-RAPTOR (Ser792)	Cell Signaling Technology	Cat#2083
Rabbit monoclonal anti-phospho-AMPKα, (Thr172) (40H9)	Cell Signaling Technology	Cat#2535
Rabbit polyclonal anti-mTOR	Cell Signaling Technology	Cat#2972
Rabbit monoclonal anti-DEPTOR (D9F5)	Cell Signaling Technology	Cat#11816
Rabbit monoclonal anti-LST8/GβL (86B8)	Cell Signaling Technology	Cat#3274
Rabbit monoclonal anti-S6rp (5G10)	Cell Signaling Technology	Cat#2217
Rabbit polyclonal anti-S6K1	Cell Signaling Technology	Cat#9202
Rabbit polyclonal anti-4E-BP1	Cell Signaling Technology	Cat#9452
Rabbit polyclonal anti-p62 (SQSTM1)	MBL Lifescience	Cat#PM045
Mouse monoclonal anti-LC3B	Nanotools	Cat#0260-100/LC3-2G6
Rabbit polyclonal anti-SHARPIN	ProteinTech	Cat#14626-1-AP
Rat monoclonal anti-HA-Tag (3F10)	Roche	Cat#11867423001
Mouse monoclonal anti-FLAG M2^®^	Sigma-Merck	Cat#F3165
Mouse monoclonal anti-Vinculin	Sigma-Merck	Cat#V9131
Rabbit monoclonal anti-linear ubiquitin (1E3) ZooMAb®	Sigma-Merck	Cat#ZRB2114
InVivoSIM anti-human TNFα (Adalimumab Biosimilar)	BioXCell	Cat#SIM0001
Secondary HRP-conjugated rabbit anti-mouse IgG (light chain specific) (D3V2A)	Cell Signaling Technology	Cat#58802
Secondary HRP-conjugated goat anti-mouse IgG	Southern Biotech	Cat#1030-05
Secondary HRP-conjugated goat anti-rabbit IgG	Southern Biotech	Cat#4030-05
Secondary HRP-conjugated goat anti-rat IgG	Southern Biotech	Cat#3030-05
Secondary HRP-conjugated mouse anti-rabbit IgG (light chain specific)	Southern Biotech	Cat#4060-05
**Bacterial** **strains:**		
*E. coli* Stellar™	Takara Bio	Cat#636763
*E. coli* BL21(DE3)	Agilent	Cat#200131
*E. coli* Rosetta (DE3)	Merck Novagen	Cat#70954
*E. coli* max efficiency™ DH10Bac	Invitrogen	Cat#10361012
**Chemicals, peptides, and recombinant proteins:**		
2-deoxyglucose (2DG)	Sigma-Aldrich	Cat#D8375
ANTI-FLAG® M2 Affinity Gel	Sigma-Aldrich	Cat#A2220
Antimycin A	Sigma-Aldrich	Cat#A8674
Bafilomycin A1 (CAS 88899-55-2)	Santa Cruz Biotechnology	Cat#sc-201550B
Brilliant Blue FCF (C.I. 42090; CAS 3844-45-9)	Carl Roth	Cat#2981.3
Carbonyl cyanide p-triflouromethoxyphenylhydrazone (FCCP)	Sigma-Aldrich	Cat#C2920
cOmplete™, EDTA-free Protease Inhibitor Cocktail	Merck	Cat#11873580001
Cycloheximide (CAS 66-81-9)	Santa Cruz Biotechnology	Cat#sc-3508A
Gibco™ Human TNF-alpha Recombinant Protein	Thermo Fisher Scientific	Cat#PHC3013
Glutathione High-Capacity Magnetic Agarose Beads	Merck	Cat#G0924
Glutathione Sepharose™ 4B	Merck	Cat#17-0756-01
L-glutamine	Thermo Fisher Scientific	Cat#25030024
Lysyl EndopeptidaseR, MS Grade (Lys-C)	FUJIFILM Wako	Cat#125-05061
Magnetic Beads Anti-FLAG® Mouse Monoclonal Antibody [M2]	Sigma-Aldrich	Cat#M8823
MK-8722	Selleckchem	Cat#E1031
Monoclonal Anti-HA−Agarose antibody produced in mouse	Sigma-Aldrich	Cat#A2095
Oligomycin	Sigma-Aldrich	Cat#O4876
PhosSTOP™ tablets	Merck	Cat#4906845001
Pierce™ Protein A/G Magnetic Beads	Thermo Fisher Scientific	Cat#88802
Pierce™ Protein A/G Plus Agarose	Thermo Fisher Scientific	Cat#20423
Recombinant GST-NEMO-CoZi-His (M1-SUB)	This study	N/A
Recombinant GST-OTU-His, OTULIN aa 80-348, C129A (M1-Trap)	This study	N/A
Lipofectamine® RNAiMAX Transfection Reagent	Thermo Fisher Scientific	Cat#13778075
Rotenone	Sigma-Aldrich	Cat#45656
Sodium Pyruvate	Thermo Fisher Scientific	Cat#11360070
Strep-Tactin™XT Superflow™ resin	IBA Lifesciences	Cat#15583756
SytoxGreen	Thermo Fisher Scientific	Cat#S7020
Thermo Scientific™ Pierce™ Anti-HA Magnetic Beads	Thermo Fisher Scientific	Cat#13474229
TMTpro™ 16plex Label Reagent Set	Thermo Fisher Scientific	Cat#A44520
Trypsin Gold, MS Grade	Promega	Cat#V5280
X-tremeGENE™ HP DNA Transfection Reagent	Merck	Cat#6366546001
**Critical commercial assays:**		
Pierce™ BCA Protein Assay Kit	Thermo Fisher Scientific	Cat#23225
Pierce™ Dilution-Free™ Rapid Gold BCA Protein Assay	Thermo Fisher Scientific	Cat#A55860
Quick Start™ Bradford 1× Dye Reagent	Bio-Rad	Cat#5000205
SOLAμ™ SPE Plates	Thermo Fisher Scientific	Cat#60209-001
**Deposited data:**		
*Proteomics:*		
OTULIN interactome: MassIVE (https://massive.ucsd.edu/ProteoSAFe/static/massive.jsp) dataset identifier MSV000096400; ftp://massive-ftp.ucsd.edu/v07/MSV000096400/		
AMPKα1 interactome: MassIVE (https://massive.ucsd.edu/ProteoSAFe/static/massive.jsp) dataset identifier MSV000096389; ftp://massive-ftp.ucsd.edu/v07/MSV000096389/		
Phosphoproteomics: MassIVE (https://massive.ucsd.edu/ProteoSAFe/static/massive.jsp) dataset identifier MSV000096382; ftp://massive-ftp.ucsd.edu/v07/MSV000096382/		
**Experimental models: cell lines**		
AML12	Provided by Prof. Sabine Werner	N/A
AML12 HOIP-KO clone C3	This study	N/A
AML12 OTULIN-KO clone C2	This study	N/A
HEK293T	Provided by Prof. M. Gyrd-Hansen	N/A
HepG2	Provided by Prof. G. van Loo	N/A
HepG2 OTULIN-KO clone C11	This study	N/A
HepG2 HOIP-KO clone A15	This study	N/A
Human dermal fibroblasts from healthy donor	Damgaard et al. [5]	N/A
Human dermal fibroblasts from ORAS patient (OTULIN^G281R^)	Damgaard et al. [5]	N/A
MEF *Hoip*-C879S	Provided by Prof. Sir P. Cohen; Emmerich et al. [37]	N/A
MEF *Hoip*-WT	Provided by Prof. Sir P. Cohen; Emmerich et al [37]	N/A
Sf9	Provided by Prof. P.R. Elliott	N/A
U2OS	Provided by Prof. M. Gyrd-Hansen	N/A
U2OS AMPKα1/2-DKO	Provided by Prof. K. Sakamoto; Sanders et al. [62]	N/A
U2OS HOIP-KO clone C33	This study	N/A
U2OS OTULIN-KO clone C1	This study	N/A
**Experimental models: Organisms/strains:**		
*Drosophila melanogaster* Canton^S^	A. Meinander lab	N/A
*Drosophila melanogaster* Daughterless-Gal4 (*DaGal4)*	A. Meinander lab	N/A
*Drosophila melanogaster* Mi{ET1}LUBELMB00197 (*lubel*^*ΔRBR*^*)*	Bloomington *Drosophila* Stock Center	Cat#22725
**Oligonucleotides:**		
Ambion™ Silencer™ Select Pre-Designed siRNA: siHOIP#1 (s30109)	Thermo Fisher Scientific	Cat#4392420- s30109
Ambion™ Silencer™ Select Pre-Designed siRNA: siHOIP#2 (s30110)	Thermo Fisher Scientific	Cat#4392420- s30110
cDNA amplification primer for PRKAA1 3’-end: GCCCTCTAGACTCGAGTTATTGTGCAAGAATTTTAATTAGA	This study	N/A
cDNA amplification primer for PRKAA1 5’-end: ATCACACTGGCGGCCGCTGCGCAGACTCAGTTCCT	This study	N/A
cDNA amplification primer for PRKAA2 3’-end: GCCCTCTAGACTCGAGTCAACGGGCTAAAGTAGTAATCAGA	This study	N/A
cDNA amplification primer for PRKAA2 5’-end: ATCACACTGGCGGCCGCTGGCTGAGAAGCAGAAGCACG	This study	N/A
cDNA amplification primer for LKB1 3’-end: TCACTGCTGCTTGCAGGCCGACAG	This study	N/A
cDNA amplification primer for LKB1 5´-end: ATGGAGGTGGTGGACCCGCAGCAG	This study	N/A
gRNA targeting human OTULIN #1: CACCGGAATTGCTTATACATGAAAG	Draber et al. [79]	N/A
gRNA targeting human OTULIN #2: CACCGGTGCGCCGAGACGCCGGCG	Verboom et al. [80]	N/A
gRNA targeting human HOIP: CACCGATGCAAGTTCTCGTACGCCC	Hua et al. [81]	N/A
gRNA targeting mouse *Hoip* #1 (exon 3) CACCGGAACTATGAGTTGTTGGACG	Freeman et al. [82]	N/A
gRNA targeting mouse *Hoip* #2 (exon 10) CACCGGATGGATTGAGTTTCCCCGA	Freeman et al. [82]	N/A
Sequencing forward primer CMV 5’-end: CGCAAATGGGCGGTAGGCGTG	This study	N/A
Sequencing reverse primer pcDNA3.1 3’-end: TAGAGCCCCAGCTGGTTCTTTCCGC	This study	N/A
siRNA targeting sequence negative control (siCtrl): GGGAUACCUAGACGUUCUA	Poulsen et al. [83]	N/A
siRNA targeting sequence OTULIN: GACUGAAAUUUGAUGGGAA	Keusekotten et al. [3]	N/A
siRNA targeting sequence RBCK1/HOIL-1: GCUCAGAUGCACACCGUCA	Lewis et al. [84]	N/A
siRNA targeting sequence SHARPIN: CCUGGAAACUUGACGGAGA	Lewis et al. [84]	N/A
**Recombinant DNA:**		
pcDNA3-2xFLAG-2xStrep-LKB1	This study	N/A
pcDNA3-2xFLAG-2xStrep-PRKAA1	This study	N/A
pcDNA3-2xFLAG-2xStrep-PRKAA2	This study	N/A
pcDNA3-2xHA-2xStrep-HOIP-C885S	This study	N/A
pcDNA3-2xHA-2xStrep-HOIP-WT	This study	N/A
pcDNA3-2xHA-2xStrep-mmHOIP-C879S (mouse)	This study	N/A
pcDNA3-2xHA-2xStrep-mmHOIP-WT (mouse)	This study	N/A
pcDNA3-2xHA-2xStrep-OTULIN-C129A	This study	N/A
pcDNA3-2xHA-2xStrep-OTULIN-deltaC (1-348)	This study	N/A
pcDNA3-2xHA-2xStrep-OTULIN-deltaN (80-352)	This study	N/A
pcDNA3-2xHA-2xStrep-OTULIN-WT	Fiil et al. [32]	N/A
pcDNA3-3xHA (empty backbone)	M. Gyrd-Hansen lab	N/A
pcDNA3-V5/His-HOIL-1	D. Komander lab	N/A
pcDNA3-V5/His-SHARPIN	D. Komander lab	N/A
pcDNA3-V5-HOIP	D. Komander lab	N/A
pGEX6P1-GST-M1-SUB (NEMO-CoZi)	Fiil et al. [32]	N/A
pGEX6P1-GST-M1-Trap (OTULIN aa 80-348, C129A)	This study	N/A
pSpCas9(BB)-2A-EGFP (PX458)	Addgene/Feng Zhang [85]	Cat#48138
pSpCas9(BB)-2A-Puro (PX459)	Addgene/Feng Zhang [85]	Cat#48139
pACEBac Twin Strep mScarlet 3 C protease UBA1	This study	N/A
pBIG Twin Strep mScarlet 3 C protease HOIP, HOIL-1, SHARPIN	This study	N/A
pOPIN B UBE2D2	This study	N/A
**Software, algorithms, and online resources:**		
clusterProfiler R package (v 4.12.6)	Wu et al. [78]	RRID:SCR_016884
EnrichmentBrowser R package (v 2.34.1)	Geistlinger et al. [72]	N/A
ggplot2 R package (v 3.5.1)	Wickham [86]	RRID:SCR_014601
GraphPad Prism 9 and 10	GraphPad Software	RRID:SCR_002798
Image Lab (v 6.1.0)	Image Lab Software	RRID:SCR_014210
Fiji (Fiji Is Just ImageJ) (v 2.14.0)	ImageJ Software	RRID:SCR_002285
KSEA App R package (v 1.0)	Wiredja et al. [77]; Casado et al. [87]	N/A
limma R package (v 3.60.6)	Ritchie et al. [71]	RRID:SCR_010943
Mfuzz R package (v 2.64.0)	Kumar and Futschik [76]	RRID:SCR_000523
OmnipathR R package (3.12.4)	Türei et al. [88]	N/A
pheatmap R package (v 1.0.12)	Kolde [89]	RRID:SCR_016418
ProteomeDiscoverer (v 2.4 and 2.5)	Thermo Fisher	RRID:SCR_014477
R (v 4.4.1)	R Core Team	RRID:SCR_001905
RStudio (v 2024.09.0 + 375)	Posit Software	RRID:SCR_000432
Seahorse Wave Desktop Software	Agilent	RRID:SCR_014526
tidyverse R packages (v 2.0.0)	Wickham et al. [90]	RRID:SCR_019186
VennDiagram R package (v 1.7.3)	Lam et al. [91]	RRID:SCR_002414
DepMap, Broad (2025). DepMap Public Portal 25Q3. https://depmap.org/portal/	Arafeh et al. [92]	RRID:SCR_017655

List of key reagents and resources, including their identifiers, used in this study.

### Cell culture

AML12, HEK293T, HepG2, and U2OS cell lines, as well as primary mouse embryonic fibroblast (MEFs), were cultured in Dulbecco’s Modified Eagle’s Medium (DMEM) with high glucose, GlutaMAX™, and pyruvate (Gibco, 31966047) supplemented with 10% (v/v) foetal bovine serum (FBS) (Biowest, S181B) and 1% (v/v) penicillin-streptomycin (PS) (Gibco, L0022). U2OS cells with double knockout (DKO) of AMPKα1 and AMPKα2 [62], as well as WT MEFs and MEFs with knock-in of HOIP^C879S^ [37], have been described previously. All cells were kept at 37 °C in humidified incubators with 5% CO_2_ pressure under mycoplasma-free conditions and tested regularly for contamination. Human fibroblast cell lines derived from an ORAS patient harbouring the OTULIN^G281R^ mutation and a healthy, unrelated donor were previously described [5] and cultured in Ham’s F-12 Nutrient Mix supplemented with 20% FBS and 1% PS. Written informed consent was obtained from all subjects and family members and approved by the ethical committee of Hadassah Medical Center and the Ministry of Health, Israel [5]. Mutational landscapes of U2OS and HepG2 cells are listed in Table S6.

### Mice

The C57BL/6N-Rnf31^<tm1c(KOMP)Wtsi>/Tcp^ mouse line was made at the Toronto Centre for Phenogenomics [63], and the B6.129-*Gt(ROSA)26Sor*^tm1(cre/ERT2)Tyj/J^ were from the University of Copenhagen transgenic mouse library. Both lines were rederived on C57BL/6N background and crossed to generate CreERT2-*Hoip*^flox/flox^ mice. Tamoxifen was dissolved in a vehicle of 10% absolute ethanol and 90% sunflower seed oil at 20 mg/mL and was administered to 10–14-week-old female mice by intraperitoneal injection (75 mg/kg bodyweight) every day for 5 days to induce deletion of *Hoip*; control mice were injected with an equal volume of vehicle only. Body weight was monitored daily. Whole-body composition analysis was conducted by TD-NMR (Minispec LF90II, Bruker). *Ad libitum*-fed blood glucose was measured once before anaesthesia for hepatocyte isolation using a handheld glucometer (Contour NEXT, Bayer). Euthanasia was confirmed by cervical dislocation. *Hoip*^flox/flox^-CreERT2^+^ mice were used in experiments with *Hoip*^flox/flox^- or *Hoip*^WT/flox^-CreERT2^-^ animals as controls.

Mice with adipocyte-specific deletion of *Hoip (Hoip*^A-KO^*)* were generated by crossing the *Hoip*^flox/flox^ mice [6] with AdipoQ.cre Strain #:010803 B6;FVB^Tg(Adipoq-cre)1Evdr/J^ from The Jackson Laboratories as described previously [64]. From the age of 6 weeks, male mice were fed a normal diet (DIO LS, 13% fat, 11% sucrose; Ssniff, E15748-04). Body weight was measured weekly. At 22–23 weeks, mice were euthanised using Ketamine/Xylazine Cocktail (100 mg/kg Ketamine and 20 mg/kg Xylazine) with a dose of 5 µL/g, followed by heart puncture, cervical dislocation, and tissue sampling. Agarose gel electrophoresis was used to genotype mouse ear DNA for the presence of WT and mutant alleles. Genomic DNA was extracted and amplified using allele-specific primers. PCR products were separated on a 2% agarose gel stained with SYBR Safe, and bands were visualised under UV light. *Hoip*^flox/flox^-*Adipoq*Cre^+^ mice were used in experiments with *Hoip*^flox/flox^-*Adipoq*Cre^-^ animals as controls.

*Otulin*^Δhep^ mice with constitutive albumin-Cre-driven hepatocyte-specific deletion of OTULIN were described previously [17].

All mice were housed under specific pathogen-free conditions. No method of randomisation was applied. All experiments were conducted with the approval of the Danish Animal Experiments Inspectorate; the German Federal Ministry for Nature, Environment and Consumers’ Protection of the state of North Rhine-Westphalia; United Kingdom Home Office; and the MRC Centre Ethical Review Committee.

### *Drosophila melanogaster*

*Drosophila melanogaster* flies were maintained at 25 °C with a 12hour light-dark cycle on medium containing 0.6% (w/v) agar, 6.5% (w/v) malt, 3.2% (w/v) semolina, 1.8% (w/v) baker’s yeast, 2.4% nipagin, and 0.7% propionic acid. Mi{ET1}LUBELMB00197 (referred to as *lubel*^∆RBR^) were obtained from Bloomington and *Daughterless-Gal4* (referred to as *DaGal4*) and *Canton*^S^ flies were used as controls.

### Cell treatments and stimulations

For glucose starvation experiments, cells were washed with PBS and incubated in glucose-free DMEM (without sodium pyruvate) (Gibco, 11966025), supplemented with 10% FBS and 1% PS. Control cells were similarly washed and incubated in fresh growth medium containing 4.5 g/L D-glucose. For Bafilomycin A1 treatment, cells were incubated either in regular growth medium, glucose-free DMEM, or Hank’s Balanced Salt Solution (HBSS) for 2 h with or without 100 nM Bafilomycin A1. For protein stability assay, cells were treated with either 100 µM cycloheximide or DMSO for up to 6 h, under both high glucose and glucose starvation conditions. NF-κB signalling was assessed by treating cells with 10 ng/mL human TNF (Thermo Fisher Scientific) for 10 min. To block TNF signalling, cells were pre-treated with 1 µg/mL anti-TNF antibody (BioXCell, Adalimumab biosimilar) for 1 h, followed by glucose starvation or 10 ng/mL TNF treatment with continuous TNF neutralisation.

### Plasmids and cloning

Inserts were PCR-amplified from in-house constructs or from cDNA from HEK293T cells using the SV Total RNA Isolation System (Promega, Z3101) and QuantiTect Reverse Transcription Kit (Qiagen). Purified PCR products were cloned into pcDNA3.1, pSpCas9, or pGEX6P1 vectors using In-Fusion primers and cloning kit (Takara Bio, 638948). Mutations were generated by overlap extension PCR and confirmed by sequencing. The M1-Trap was generated by inserting human OTULIN (80-348); C129A into pGEX6P1. Plasmids were amplified in Stellar™ competent *E. coli* (Thermo Fisher Scientific).

### Plasmid DNA transfection

Cells at ~80% confluency were transfected using X-tremeGENE HP (Merck) at a 2:1 (v/w) ratio according to the manufacturer’s instructions 24–48 h before additional treatments, stimulations, or lysis.

### Generation of knockout cells using CRISPR-Cas9

Knockout cells were generated by transfection or electroporation of gRNAs encoded in PX458 or PX459. PX458 and PX459 were gifts from Professor Feng Zhang (Addgene plasmids #48138 and #48139). gRNA sequences can be found in Table 1. Full experimental details are provided in the Supplementary Methods.

### siRNA transfection

Cells were transfected with the oligos listed in Table 1 using Lipofectamine® RNAiMAX Transfection Reagent (Thermo Fisher Scientific). Full experimental details are provided in the Supplementary Methods.

### Isolation of primary hepatocytes

Murine hepatocytes were isolated as described previously [65]. Viable cells were diluted in plating medium supplemented with 0.1 μM dexamethasone (Sigma, D4902) and 1 nM insulin (Sigma, I9278) and seeded onto collagen-coated plates at a density of 200,000 cells/mL and allowed to attach for 2–4 h before changing to culture medium (low-glucose DMEM; Gibco, 10567014; with 10% FBS and 1% PS). The following day, the hepatocytes were used for experiments. Culture medium with glucose-free DMEM was used for hepatocyte starvation (Gibco, 11966025). Full experimental details are provided in the Supplementary Methods.

### Cell and tissue lysis and protein extraction for immunoblotting

Cells, mouse tissues, and flies were lysed using appropriate buffers with protease and phosphatase inhibitors, and in some cases with mechanical homogenisation. Lysates were mixed with 4× Laemmli sample buffer (LSB) to obtain a 1× concentration (Tris pH 6.8, 10% glycerol (v/v), 100 mM DTT, 2% SDS (w/v), bromophenol blue) and heated heat-denatured for downstream analysis. Full experimental details are provided in the Supplementary Methods.

### Immunoprecipitation and affinity-precipitation

Cells were transfected or treated as indicated, washed with ice-cold PBS, and lysed on ice in IP buffer (25 mM HEPES (pH 7.4), 150 mM KCl, 2 mM MgCl₂, 1 mM EGTA, 0.5% Triton X-100) supplemented with cOmplete™ Protease Inhibitor Cocktail (Merck), PhosSTOP™ (Merck), and 5 mM NEM. Lysates were cleared by centrifugation at 13,000 rcf for 10 min at 4 °C, and supernatants were transferred to clean tubes and supplemented with 1 mM DTT. For endogenous IPs, primary antibodies (concentrations according to manufacturers’ recommendations) were added along with 20 µL bead slurry/sample pre-washed protein A/G agarose beads (Thermo Scientific) or magnetic beads (Thermo Scientific) and incubated with lysates at 4 °C for 2–18 h with gentle agitation. For endogenous HOIP IPs, samples were supplemented with 10% (v/v) glycerol. For tagged protein immunoprecipitation, lysates were incubated with either 20 µL bead slurry/sample pre-washed magnetic anti-HA beads (Thermo Scientific) or pre-washed magnetic anti-FLAG^®^ M2 beads (Sigma-Aldrich). For affinity-precipitation, lysates were incubated with 40 µL bead slur/sample of pre-washed Strep-Tactin™XT Superflow™ (IBA Lifesciences). After IP or pulldown, beads were washed two times with 500 µL of ice-cold high-salt IP buffer (IP buffer with 1 M KCl) followed by two times with 500 µL of ice-cold regular IP buffer. After incubation and washing, bound material was eluted in either LSB (for SDS-PAGE and immunoblotting) by heating to 60 °C for 5 min, or in MS lysis buffer (6 M guanidinium hydrochloride, 10 mM TCEP, 40 mM CAA, 50 mM HEPES, pH 8.5) for mass spectrometry analysis.

### Purification of M1-linked Ubiquitin Conjugates

M1-linked Ub conjugates were purified using either the M1-SUB or the M1-Trap (Table 1) as previously described [32, 66]. Full experimental details are provided in the Supplementary Methods.

### Immunoblotting

LSB-supplemented lysates were resolved by SDS-PAGE on NuPAGE Tris-Acetate 3–8% or Bis-Tris 4–12% gels according to manufacturer instructions, or self-cast 9% Bis-Tris gels. Proteins were transferred to 0.2 µm nitrocellulose membranes using the Trans-Blot Turbo system (Bio-Rad). Membranes were stained with Ponceau S to verify transfer efficiency and equal loading, then blocked in 5% skimmed milk powder in PBS-T. Membranes were incubated overnight at 4 °C with primary antibodies (Table 1) in 3% (w/v) BSA and 0.01% NaN₃ in PBS-T. Membranes were then washed with PBS-T and incubated with HRP-conjugated secondary antibodies (Table 1) for 45 min at room temperature. After washing, blots were developed using Clarity™ Western ECL substrate (Bio-Rad, 1705061), visualised on a Bio-Rad ChemiDoc™ MP Imager, and processed using the Image Lab software (Bio-Rad). Densitometric analysis of immunoblots was performed using Fiji (Fiji Is Just ImageJ) by calculating the area under the curve for bands of interest, subtracting local background, and normalising the intensity of each phosphorylated protein band to the intensity of its corresponding total protein band or an appropriate loading control.

### Immunoprecipitation-coupled AMPK kinase assays

Cells were lysed in lysis buffer (50 mM Tris–HCl (pH 7.4), 150 mM NaCl, 1% (v/v) Triton X-100, 50 mM NaF, 5 mM sodium pyrophosphate, 1 mM EDTA, 1 mM EGTA, 1 mM DTT, 0.1 mM benzamidine, 0.1 mM PMSF) and AMPK was immunoprecipitated using anti-AMPK-α1 and -α2 antibodies and protein G-Sepharose beads. After washing, AMPK-bound beads were incubated with AMARA peptide [67] and [γ-^33^P]-ATP (Hartmann Analytic). Following incubation, reactions were captured on P81 filters, washed, and quantified by scintillation counting. Full experimental details are provided in the Supplementary Methods.

### Seahorse XF bioenergetics measurements

Cells were seeded into XF96 Polystyrene Cell Culture Plate (Agilent Technologies) 24–48 h before assay at 30,000 cells per well. On the day of the assay, the assay medium was prepared fresh using Seahorse XF base medium (Agilent Technologies) adjusted to pH 7.4, supplemented with 2 mM L-glutamine (Thermo Fisher Scientific) and optionally 10 mM glucose (Merck) and 1 mM sodium pyruvate (Merck). One hour before the assay, cells were incubated with assay medium, with or without glucose and sodium pyruvate, then incubated at 37 °C in an incubator absent of CO_2_. Six baseline OCR and ECAR recordings were made, followed by sequential injection of ATP synthase inhibitor oligomycin (1 μM, Merck), mitochondrial uncoupler carbonyl cyanide p-triflouromethoxyphenylhydrazone (FCCP; 1 μM, Merck), the Complex I and III inhibitors rotenone and antimycin A (R/A; 5 μM, Merck), and the glucose analogue 2-deoxyglucose (2DG; 50 mM, Merck). Mitochondrial respiration and glycolytic activity were measured using the Seahorse XFe96 Analyzer (Agilent Technologies) with downstream analysis using the Seahorse Wave Desktop Software (Agilent Technologies). Measurements were normalised to total well protein content using the Pierce™ BCA Protein Assay Kit (Thermo Fisher Scientific).

### Starvation and survival in Drosophila flies and larvae

Adult flies (20–30 flies per genotype) were starved in tubes containing a layer of 2% agarose for up to 48 h and the survival was counted at indicated time points.

### Smurf assay in Drosophila larvae

Smurf assays were performed using Brilliant Blue FCF (Carl Roth). Control larvae were transferred to standard fly food supplemented with 7.8 mL of 10% Brilliant Blue stock solution per 100 mL of food and incubated for 3 h. For starvation, larvae were placed on 2% agarose prepared with the same concentration of dye for 3 h. Following starvation, larvae were imaged on a Leica DFC290HD camera with a 2.5× objective. The number of Smurf-positive larvae (systemic dye leakage beyond the gut) was quantified from the photographs by two independent, blinded scorers.

### Cell death assay

Cells were seeded at 10,000 cells per well in 48 well plates. Twenty four hours after seeding, cells medium was changes to either complete or glucose-free DMEM containing SytoxGreen (200 nM, ThermoFisher). Cells were imaged on an IncuCyte S3 live cell analysis system (Sartorius). Four images per well were taken at 60-min intervals for 48 h, then switched to 6-hour intervals for the remaining 24 h for a total of 72 h. The relative proportion of dead cells was determined by dividing SytoxGreen-positive cells by confluence using IncuCyte software.

### Purification of ubiquitin affinity reagents

The M1-SUB (GST-NEMO-CoZi-His) and M1-Trap (GST-OTULIN^80-348^-His; C129A) were purified from *E. coli* BL21(DE3) by IMAC or GST purification. Full experimental details are provided in the Supplementary Methods.

### Purification of recombinant E1, E2, and LUBAC

A single co-expressing LUBAC, consisting of full-length human HOIP, HOIL-1, and SHARPIN, generated using the pBIG cloning strategy [68] and purified from Sf9 cells. E1 (human UBA1) was purified from Sf9 cells and E2 (human UBE2D2) purified from Rosetta DE3 *E. coli* as previously described [69]. Full experimental details are provided in the Supplementary Methods.

### In vitro ubiquitination assay

HEK293T cells were transfected with plasmids encoding HA-tagged AMPKα1 and AMPKα2 in a 1:1 ratio and immunoprecipitated using anti-HA beads (Thermo Scientific). Beads with purified HA-tagged AMPKα1/2 were resuspended in reaction buffer (40 mM HEPES (pH 7.3), 10 mM MgCl_2_, 0.6 mM DTT) including 150 µM Ub, 100 nM E1 (UBA1), 500 nM E2 (UBE2D2), 1 µM LUBAC, and 10 mM ATP in a reaction volume of 50 µL per sample. Samples were incubated at 37 °C on a shaker at 1300 rpm. Reactions were stopped by adding DTT to a final concentration of 10 mM. Bound material was eluted and denatured in LSB for 2 min at 70 °C before analysis by Coomassie staining or immunoblotting.

### Nucleotide extraction and measurement of ATP, ADP and AMP

Nucleotides were extracted and analysed by liquid chromatography-tandem mass spectrometry (LC-MS/MS) on a TSQ Quantiva interfaced with Ultimate 3000 Liquid Chromatography system (Thermo-Scientific) as described previously [70]. Full experimental details are provided in the Supplementary Methods. The relative abundances of AMP, ADP and ATP were converted to AEC defined as:

$${{\rm{AEC}}}=\frac{[{{\rm{ATP}}}]\times 0.5[{{\rm{ADP}}}]}{[{{\rm{ATP}}}]+[{{\rm{ADP}}}]+[{{\rm{AMP}}}]}$$

### Proteomics and phosphoproteomics

Proteomic and phosphoproteomic analyses were performed LC-MS/MS on Q-Exactive or Exploris instruments (Thermo Fisher Scientific). Enrichment, analyses, and quantification relied on label-free or TMT-based workflows, with stringent filtering, normalisation, imputation, and statistical testing using limma as previously described [71–74]. Differential protein and phosphosite regulation, pathway mapping, clustering, and kinase-substrate enrichment analyses were performed in R [75–78]. Full experimental details are provided in the Supplementary Methods.

### Statistical testing

Data are presented as mean ± standard error of the mean (SEM). The sample size (*n*) is the number of independent biological replicates for each experiment. Sample numbers for each experiment are provided in the figures and figure legends. Statistical analyses were performed using R (Posit) or GraphPad Prism. Depending on the data structure and distribution, the following tests were applied: Student’s *t*-test for parametric two-group comparisons, Mann–Whitney U test for non-parametric two-group comparisons, Two-way ANOVA followed by Tukey’s post-hoc test for multiple group comparisons, or Fisher’s exact test for categorical data. Where applicable, corrections for multiple comparisons were applied. For time-course comparisons of densitometric immunoblot analyses, generalised additive models (GAMs) were used in R to account for non-linear dynamics over time. Statistical significance is reported as: ns (not significant, *p* > 0.05), * (*p* < 0.05), ** (*p* < 0.01), *** (*p* < 0.001), **** (*p* < 0.0001).

## Supplementary information

Supplementary Methods

Supplementary Figures S1-S7

Figure S8

Table S1

Table S2

Table S3

Table S4

Table S5

Table S6

## Data Availability

Proteomics data analyses are available as Supplementary Tables 1–5 and the data has been deposited in public repositories (see Table 1: Regents and Resources). Uncropped immunoblots are available in Fig. S8. Further information and requests for resources or reagents should be directed to corresponding author, Rune Busk Damgaard, e-mail: rudam@dtu.dk.

## References

[1] Jahan AS, Elbæk CR, Damgaard RB. Met1-linked ubiquitin signalling in health and disease: inflammation, immunity, cancer, and beyond. Cell Death Differ. 2021;28:473–92. 33441937 10.1038/s41418-020-00676-w PMC7862443

[2] Kirisako T, Kamei K, Murata S, Kato M, Fukumoto H, Kanie M, et al. A ubiquitin ligase complex assembles linear polyubiquitin chains. Embo J. 2006;25:4877–87. 17006537 10.1038/sj.emboj.7601360 PMC1618115

[3] Keusekotten K, Elliott PR, Glockner L, Fiil BK, Damgaard RB, Kulathu Y, et al. OTULIN antagonizes LUBAC signaling by specifically hydrolyzing Met1-linked polyubiquitin. Cell. 2013;153:1312–26. 23746843 10.1016/j.cell.2013.05.014 PMC3690481

[4] Rivkin E, Almeida SM, Ceccarelli DF, Juang Y-C, MacLean TA, Srikumar T, et al. The linear ubiquitin-specific deubiquitinase Gumby regulates angiogenesis. Nature. 2013;498:318–24. 23708998 10.1038/nature12296 PMC4931916

[5] Damgaard RB, Elliott PR, Swatek KN, Maher ER, Stepensky P, Elpeleg O, et al. OTULIN deficiency in ORAS causes cell type-specific LUBAC degradation, dysregulated TNF signalling and cell death. Embo Mol Med. 2019;11:e9324. 30804083 10.15252/emmm.201809324 PMC6404114

[6] Peltzer N, Rieser E, Taraborrelli L, Draber P, Darding M, Pernaute B, et al. HOIP deficiency causes embryonic lethality by aberrant TNFR1-mediated endothelial cell death. Cell Rep. 2014;9:153–65. 25284787 10.1016/j.celrep.2014.08.066

[7] Peltzer N, Darding M, Montinaro A, Draber P, Draberova H, Kupka S, et al. LUBAC is essential for embryogenesis by preventing cell death and enabling haematopoiesis. Nature. 2018;557:112–7. 29695863 10.1038/s41586-018-0064-8 PMC5947819

[8] Damgaard RB, Walker JA, Marco-Casanova P, Morgan NV, Titheradge HL, Elliott PR, et al. The deubiquitinase OTULIN is an essential negative regulator of inflammation and autoimmunity. Cell. 2016;166:1215–1230.e20. 27523608 10.1016/j.cell.2016.07.019 PMC5002269

[9] Gerlach B, Cordier SM, Schmukle AC, Emmerich CH, Rieser E, Haas TL, et al. Linear ubiquitination prevents inflammation and regulates immune signalling. Nature. 2011;471:591–6. 21455173 10.1038/nature09816

[10] Ikeda F, Deribe YL, Skånland SS, Stieglitz B, Grabbe C, Franz-Wachtel M, et al. SHARPIN forms a linear ubiquitin ligase complex regulating NF-κB activity and apoptosis. Nature. 2011;471:637–41. 21455181 10.1038/nature09814 PMC3085511

[11] Tokunaga F, Nakagawa T, Nakahara M, Saeki Y, Taniguchi M, Sakata S, et al. SHARPIN is a component of the NF-κB-activating linear ubiquitin chain assembly complex. Nature. 2011;471:633–6. 21455180 10.1038/nature09815

[12] Boisson B, Laplantine E, Prando C, Giliani S, Israelsson E, Xu Z, et al. Immunodeficiency, autoinflammation and amylopectinosis in humans with inherited HOIL-1 and LUBAC deficiency. Nat Immunol. 2012;13:1178–86. 23104095 10.1038/ni.2457 PMC3514453

[13] Boisson B, Laplantine E, Dobbs K, Cobat A, Tarantino N, Hazen M, et al. Human HOIP and LUBAC deficiency underlies autoinflammation, immunodeficiency, amylopectinosis, and lymphangiectasia. J Exp Med. 2015;212:939–51. 26008899 10.1084/jem.20141130 PMC4451137

[14] Oda H, Manthiram K, Chavan PP, Rieser E, Veli Ö, Kaya Ö, et al. Biallelic human SHARPIN loss of function induces autoinflammation and immunodeficiency. Nat Immunol. 2024;25:764–77. 10.1038/s41590-024-01817-w PMC11626442 38609546

[15] Zhou Q, Yu X, Demirkaya E, Deuitch N, Stone D, Tsai WL, et al. Biallelic hypomorphic mutations in a linear deubiquitinase define otulipenia, an early-onset autoinflammatory disease. Proc Natl Acad Sci USA. 2016;113:10127–32. 27559085 10.1073/pnas.1612594113 PMC5018768

[16] Nilsson J, Schoser B, Laforet P, Kalev O, Lindberg C, Romero NB, et al. Polyglucosan body myopathy caused by defective ubiquitin ligase RBCK1. Ann Neurol. 2013;74:914–9. 23798481 10.1002/ana.23963

[17] Damgaard RB, Jolin HE, Allison MED, Davies SE, Titheradge HL, McKenzie ANJ, et al. OTULIN protects the liver against cell death, inflammation, fibrosis, and cancer. Cell Death Differ. 2020;27:1457–74. 32231246 10.1038/s41418-020-0532-1 PMC7206033

[18] Verboom L, Martens A, Priem D, Hoste E, Sze M, Vikkula H, et al. OTULIN Prevents liver inflammation and hepatocellular carcinoma by inhibiting FADD- and RIPK1 kinase-mediated hepatocyte apoptosis. Cell Rep. 2020;30:2237–2247.e6. 32075762 10.1016/j.celrep.2020.01.028

[19] Steinberg GR, Hardie DG. New insights into activation and function of the AMPK. Nat Rev Mol Cell Biol. 2023;24:255–72. 36316383 10.1038/s41580-022-00547-x

[20] Gowans GJ, Hawley SA, Ross FA, Hardie DG. AMP is a true physiological regulator of AMP-activated protein kinase by both allosteric activation and enhancing net phosphorylation. Cell Metab. 2013;18:556–66. 24093679 10.1016/j.cmet.2013.08.019 PMC3791399

[21] Xin F-J, Wang J, Zhao R-Q, Wang Z-X, Wu J-W. Coordinated regulation of AMPK activity by multiple elements in the α-subunit. Cell Res. 2013;23:1237–40. 23999859 10.1038/cr.2013.121 PMC3790237

[22] Yan Y, Mukherjee S, Harikumar KG, Strutzenberg TS, Zhou XE, Suino-Powell K, et al. Structure of an AMPK complex in an inactive, ATP-bound state. Science. 2021;373:413–9. 34437114 10.1126/science.abe7565 PMC8428800

[23] Lin S-C, Hardie DG. AMPK: sensing glucose as well as cellular energy status. Cell Metab. 2018;27:299–313. 29153408 10.1016/j.cmet.2017.10.009

[24] Hardie DG, Ross FA, Hawley SA. AMPK: a nutrient and energy sensor that maintains energy homeostasis. Nat Rev Mol Cell Biol. 2012;13:251–62. 22436748 10.1038/nrm3311 PMC5726489

[25] Deleyto-Seldas N, Efeyan A. The mTOR–autophagy axis and the control of metabolism. Front Cell Dev Biol. 2021;9:655731. 34277603 10.3389/fcell.2021.655731 PMC8281972

[26] O’Neill LAJ, Hardie DG. Metabolism of inflammation limited by AMPK and pseudo-starvation. Nature. 2013;493:346–55. 23325217 10.1038/nature11862

[27] Steinberg GR, Carling D. AMP-activated protein kinase: the current landscape for drug development. Nat Rev Drug Discov. 2019;18:527–51. 30867601 10.1038/s41573-019-0019-2

[28] Jeon S-M. Regulation and function of AMPK in physiology and diseases. Exp Mol Med. 2016;48:e245–e245. 27416781 10.1038/emm.2016.81 PMC4973318

[29] Gwinn DM, Shackelford DB, Egan DF, Mihaylova MM, Mery A, Vasquez DS, et al. AMPK phosphorylation of raptor mediates a metabolic checkpoint. Mol Cell. 2008;30:214–26. 18439900 10.1016/j.molcel.2008.03.003 PMC2674027

[30] Inoki K, Zhu T, Guan K-L. TSC2 mediates cellular energy response to control cell growth and survival. Cell. 2003;115:577–90. 14651849 10.1016/s0092-8674(03)00929-2

[31] Stangl A, Elliott PR, Pinto-Fernandez A, Bonham S, Harrison L, Schaub A, et al. Regulation of the endosomal SNX27-retromer by OTULIN. Nat Commun. 2019;10:4320. 31541095 10.1038/s41467-019-12309-z PMC6754446

[32] Fiil BK, Damgaard RB, Wagner SA, Keusekotten K, Fritsch M, Bekker-Jensen S, et al. OTULIN restricts Met1-linked ubiquitination to control innate immune signaling. Mol Cell. 2013;50:818–30. 23806334 10.1016/j.molcel.2013.06.004 PMC4194427

[33] Elliott PR, Nielsen SV, Marco-Casanova P, Fiil BK, Keusekotten K, Mailand N, et al. Molecular basis and regulation of OTULIN-LUBAC interaction. Mol Cell. 2014;54:335–48. 24726323 10.1016/j.molcel.2014.03.018 PMC4017264

[34] Schaeffer V, Akutsu M, Olma MH, Gomes LC, Kawasaki M, Dikic I. Binding of OTULIN to the PUB domain of HOIP controls NF-κB signaling. Mol Cell. 2014;54:349–61. 24726327 10.1016/j.molcel.2014.03.016

[35] Fujita H, Tokunaga A, Shimizu S, Whiting AL, Aguilar-Alonso F, Takagi K, et al. Cooperative domain formation by homologous motifs in HOIL-1L and SHARPIN plays a crucial role in LUBAC stabilization. Cell Rep. 2018;23:1192–204. 29694895 10.1016/j.celrep.2018.03.112 PMC6044281

[36] Fang C, Pan J, Qu N, Lei Y, Han J, Zhang J, et al. The AMPK pathway in fatty liver disease. Front Physiol. 2022;13:970292. 36203933 10.3389/fphys.2022.970292 PMC9531345

[37] Emmerich CH, Ordureau A, Strickson S, Arthur JSC, Pedrioli PGA, Komander D, et al. Activation of the canonical IKK complex by K63/M1-linked hybrid ubiquitin chains. Proc Natl Acad Sci USA. 2013;110:15247–52. 23986494 10.1073/pnas.1314715110 PMC3780889

[38] Myers RW, Guan H-P, Ehrhart J, Petrov A, Prahalada S, Tozzo E, et al. Systemic pan-AMPK activator MK-8722 improves glucose homeostasis but induces cardiac hypertrophy. Science. 2017;357:507–11. 28705990 10.1126/science.aah5582

[39] Koch J, Elbæk CR, Priesmann D, Damgaard RB. The molecular toolbox for linkage type-specific analysis of ubiquitin signaling. ChemBioChem. 2025;26:e202500114. 40192223 10.1002/cbic.202500114 PMC12118340

[40] Rahighi S, Ikeda F, Kawasaki M, Akutsu M, Suzuki N, Kato R, et al. Specific recognition of linear ubiquitin chains by NEMO is important for NF-κB activation. Cell. 2009;136:1098–109. 19303852 10.1016/j.cell.2009.03.007

[41] Hildebrandt X, Ibrahim M, Peltzer N. Cell death and inflammation during obesity: “Know my methods, WAT(son).” Cell Death Differ. 2022;30:279–92. 10.1038/s41418-022-01062-4 PMC9520110 36175539

[42] Lee M-S, Han H-J, Han SY, Kim IY, Chae S, Lee C-S, et al. Loss of the E3 ubiquitin ligase MKRN1 represses diet-induced metabolic syndrome through AMPK activation. Nat Commun. 2018;9:3404. 30143610 10.1038/s41467-018-05721-4 PMC6109074

[43] Liu H, Ding J, Köhnlein K, Urban N, Ori A, Villavicencio-Lorini P, et al. The GID ubiquitin ligase complex is a regulator of AMPK activity and organismal lifespan. Autophagy. 2020;16:1618–34. 31795790 10.1080/15548627.2019.1695399 PMC8386601

[44] Ovens AJ, Scott JW, Langendorf CG, Kemp BE, Oakhill JS, Smiles WJ. Post-translational modifications of the energy guardian AMP-activated protein kinase. Int J Mol Sci. 2021;22:1229. 33513781 10.3390/ijms22031229 PMC7866021

[45] DAVIES SP, CARLING D, HARDIE DG. Tissue distribution of the AMP-activated protein kinase, and lack of activation by cyclic-AMP-dependent protein kinase, studied using a specific and sensitive peptide assay. Eur J Biochem. 1989;186:123–8. 2574667 10.1111/j.1432-1033.1989.tb15185.x

[46] Zhang C-S, Hawley SA, Zong Y, Li M, Wang Z, Gray A, et al. Fructose-1,6-bisphosphate and aldolase mediate glucose sensing by AMPK. Nature. 2017;548:112–6. 28723898 10.1038/nature23275 PMC5544942

[47] Yan Y, Zhou XE, Xu HE, Melcher K. Structure and physiological regulation of AMPK. Int J Mol Sci. 2018;19:3534. 30423971 10.3390/ijms19113534 PMC6274893

[48] Hsu C-C, Peng D, Cai Z, Lin H-K. AMPK signaling and its targeting in cancer progression and treatment. Semin Cancer Biol. 2022;85:52–68. 33862221 10.1016/j.semcancer.2021.04.006 PMC9768867

[49] Herzig S, Shaw RJ. AMPK: guardian of metabolism and mitochondrial homeostasis. Nat Rev Mol Cell Biol. 2018;19:121–35. 28974774 10.1038/nrm.2017.95 PMC5780224

[50] Marsin A-S, Bertrand† L, Rider MH, Deprez J, Beauloye C, Vincent‡ MF, et al. Phosphorylation and activation of heart PFK-2 by AMPK has a role in the stimulation of glycolysis during ischaemia. Curr Biol. 2000;10:1247–55. 11069105 10.1016/s0960-9822(00)00742-9

[51] Marsin A-S, Bouzin C, Bertrand L, Hue L. The stimulation of glycolysis by hypoxia in activated monocytes is mediated by AMP-activated protein kinase and inducible 6-phosphofructo-2-kinase*. J Biol Chem. 2002;277:30778–83. 12065600 10.1074/jbc.M205213200

[52] Sinnett SE, Brenman JE. The Role of AMPK in Drosophila melanogaster. Exp Suppl. 2016;107:389–401. 27812989 10.1007/978-3-319-43589-3_16 PMC5835264

[53] Asaoka T, Almagro J, Ehrhardt C, Tsai I, Schleiffer A, Deszcz L, et al. Linear ubiquitination by LUBEL has a role in Drosophila heat stress response. Embo Rep. 2016;17:1624–40. 27702987 10.15252/embr.201642378 PMC5090701

[54] Aalto AL, Mohan AK, Schwintzer L, Kupka S, Kietz C, Walczak H, et al. M1-linked ubiquitination by LUBEL is required for inflammatory responses to oral infection in Drosophila. Cell Death Differ. 2019;26:860–76. 30026495 10.1038/s41418-018-0164-x PMC6462001

[55] Rera M, Clark RI, Walker DW. Intestinal barrier dysfunction links metabolic and inflammatory markers of aging to death in Drosophila. Proc Natl Acad Sci USA. 2012;109:21528–33. 23236133 10.1073/pnas.1215849110 PMC3535647

[56] Sasaki K, Hayamizu Y, Murakami R, Toi M, Iwai K. Linear ubiquitination-induced necrotic tumor remodeling elicits immune evasion. FEBS Lett. 2023;597:1193–212. 37060248 10.1002/1873-3468.14623

[57] Loo G, van, Bertrand MJM. Death by TNF: a road to inflammation. Nat Rev Immunol. 2023;23:289–303. 36380021 10.1038/s41577-022-00792-3 PMC9665039

[58] Elliott PR, Leske D, Hrdinka M, Bagola K, Fiil BK, McLaughlin SH, et al. SPATA2 Links CYLD to LUBAC, activates CYLD, and controls LUBAC signaling. Mol Cell. 2016;63:990–1005. 27591049 10.1016/j.molcel.2016.08.001 PMC5031558

[59] Wagner SA, Satpathy S, Beli P, Choudhary C. SPATA2 links CYLD to the TNF-α receptor signaling complex and modulates the receptor signaling outcomes. Embo J. 2016;35:1868–84. 27307491 10.15252/embj.201694300 PMC5007551

[60] Schlicher L, Wissler M, Preiss F, Brauns-Schubert P, Jakob C, Dumit V, et al. SPATA2 promotes CYLD activity and regulates TNF-induced NF-κB signaling and cell death. Embo Rep. 2016;17:1485–97. 27458237 10.15252/embr.201642592 PMC5048381

[61] Chu Y, Kang Y, Yan C, Yang C, Zhang T, Huo H, et al. LUBAC and OTULIN regulate autophagy initiation and maturation by mediating the linear ubiquitination and the stabilization of ATG13. Autophagy. 2020;17:1684–99. 10.1080/15548627.2020.1781393 PMC8354604 32543267

[62] Sanders MJ, Ratinaud Y, Neopane K, Bonhoure N, Day EA, Ciclet O, et al. Natural (dihydro)phenanthrene plant compounds are direct activators of AMPK through its allosteric drug and metabolite–binding site. J Biol Chem. 2022;298:101852. 35331736 10.1016/j.jbc.2022.101852 PMC9108889

[63] Bradley A, Anastassiadis K, Ayadi A, Battey JF, Bell C, Birling M-C, et al. The mammalian gene function resource: the international knockout mouse consortium. Mamm Genome. 2012;23:580–6. 22968824 10.1007/s00335-012-9422-2 PMC3463800

[64] Hildebrandt X, Veli Ö, Hyoubi A, Zinngrebe J, Abdallah AT, Rodefeld J, et al. Linear ubiquitination prevents lipodystrophy and obesity-associated metabolic syndrome. Sci Adv. 2025;11:eadw2539. 40961178 10.1126/sciadv.adw2539 PMC12442851

[65] Gandin V, Miluzio A, Barbieri AM, Beugnet A, Kiyokawa H, Marchisio PC, et al. Eukaryotic initiation factor 6 is rate-limiting in translation, growth and transformation. Nature. 2008;455:684–8. 18784653 10.1038/nature07267 PMC2753212

[66] Hrdinka M, Fiil BK, Zucca M, Leske D, Bagola K, Yabal M, et al. CYLD limits Lys63- and Met1-linked ubiquitin at receptor complexes to regulate innate immune signaling. Cell Rep. 2016;14:2846–58. 26997266 10.1016/j.celrep.2016.02.062 PMC4819907

[67] Dale S, Wilson WA, Edelman AM, Hardie DG. Similar substrate recognition motifs for mammalian AMP-activated protein kinase, higher plant HMG-CoA reductase kinase-A, yeast SNF1, and mammalian calmodulin-dependent protein kinase I. FEBS Lett. 1995;361:191–5. 7698321 10.1016/0014-5793(95)00172-6

[68] Weissmann, Petzold F, VanderLinden G, Veld R, in PJH, t, Brown NG, Lampert F, et al. biGBac enables rapid gene assembly for the expression of large multisubunit protein complexes. Proc Natl Acad Sci USA. 2016;113:E2564–E2569. 27114506 10.1073/pnas.1604935113 PMC4868461

[69] Dietz L, Ellison CJ, Riechmann C, Cassidy CK, Felfoldi FD, Pinto-Fernández A, et al. Structural basis for SMAC-mediated antagonism of caspase inhibition by the giant ubiquitin ligase BIRC6. Science. 2023;379:1112–7. 36758106 10.1126/science.ade8840

[70] Ross FA, Hawley SA, Auciello FR, Gowans GJ, Atrih A, Lamont DJ, et al. Mechanisms of paradoxical activation of AMPK by the kinase inhibitors SU6656 and Sorafenib. Cell Chem Biol. 2017;24:813–824.e4. 28625738 10.1016/j.chembiol.2017.05.021 PMC5522529

[71] Ritchie ME, Phipson B, Wu D, Hu Y, Law CW, Shi W, et al. limma powers differential expression analyses for RNA-sequencing and microarray studies. Nucleic Acids Res. 2019;43:e47–e47. 10.1093/nar/gkv007 PMC4402510 25605792

[72] Geistlinger L, Csaba G, Zimmer R. Bioconductor’s EnrichmentBrowser: seamless navigation through combined results of set- & network-based enrichment analysis. BMC Bioinform. 2016;17:45. 10.1186/s12859-016-0884-1 PMC4721010 26791995

[73] Batth TS, Francavilla C, Olsen JV. Off-line high-pH reversed-phase fractionation for in-depth phosphoproteomics. J Proteome Res. 2014;13:6176–86. 25338131 10.1021/pr500893m

[74] Post H, Penning R, Fitzpatrick MA, Garrigues LB, Wu W, MacGillavry HD, et al. Robust, sensitive, and automated phosphopeptide enrichment optimized for low sample amounts applied to primary hippocampal neurons. J Proteome Res. 2017;16:728–37. 28107008 10.1021/acs.jproteome.6b00753

[75] Ting L, Cowley MJ, Hoon SL, Guilhaus M, Raftery MJ, Cavicchioli R. Normalization and statistical analysis of quantitative proteomics data generated by metabolic labeling*. Mol Cell Proteomics. 2009;8:2227–42. 19605365 10.1074/mcp.M800462-MCP200 PMC2758752

[76] Kumar L, Futschik ME. Mfuzz: a software package for soft clustering of microarray data. Bioinformation. 2007;2:5–7. 18084642 10.6026/97320630002005 PMC2139991

[77] Wiredja DD, Koyutürk M, Chance MR. The KSEA App: a web-based tool for kinase activity inference from quantitative phosphoproteomics. Bioinformatics. 2017;33:3489–91. 28655153 10.1093/bioinformatics/btx415 PMC5860163

[78] Wu T, Hu E, Xu S, Chen M, Guo P, Dai Z, et al. clusterProfiler 4.0: a universal enrichment tool for interpreting omics data. Innovation. 2021;2:100141. 34557778 10.1016/j.xinn.2021.100141 PMC8454663

[79] Draber P, Kupka S, Reichert M, Draberova H, Lafont E, de Miguel D, et al. LUBAC-recruited CYLD and A20 regulate gene activation and cell death by exerting opposing effects on linear ubiquitin in signaling complexes. Cell Rep. 2015;13:2258–72. 26670046 10.1016/j.celrep.2015.11.009 PMC4688036

[80] Verboom L, Anderson CJ, Jans M, Petta I, Blancke G, Martens A, et al. OTULIN protects the intestinal epithelium from apoptosis during inflammation and infection. Cell Death Dis. 2023;14:534. 37598207 10.1038/s41419-023-06058-7 PMC10439912

[81] Hua F, Hao W, Wang L, Li S. Linear ubiquitination mediates EGFR-induced nf-κb pathway and tumor development. Int J Mol Sci. 2021;22:11875. 34769306 10.3390/ijms222111875 PMC8585052

[82] Freeman AJ, Vervoort SJ, Michie J, Ramsbottom KM, Silke J, Kearney CJ, et al. HOIP limits anti-tumor immunity by protecting against combined TNF and IFN-gamma-induced apoptosis. EMBO Rep. 2021;22:e53391. 34467615 10.15252/embr.202153391 PMC8567220

[83] Poulsen M, Lukas C, Lukas J, Bekker-Jensen S, Mailand N. Human RNF169 is a negative regulator of the ubiquitin-dependent response to DNA double-strand breaks. J Cell Biol. 2012;197:189–99. 22492721 10.1083/jcb.201109100 PMC3328375

[84] Lewis MJ, Vyse S, Shields AM, Boeltz S. UBE2L3 polymorphism amplifies nf-κb activation and promotes plasma cell development, linking linear ubiquitination to multiple autoimmune diseases. Am J Hum Genet. 2015;96. 10.1016/j.ajhg.2014.12.024. 10.1016/j.ajhg.2014.12.024 PMC4320258 25640675

[85] Ran FA, Hsu PD, Wright J, Agarwala V, Scott DA, Zhang F. Genome engineering using the CRISPR-Cas9 system. Nat Protoc. 2013;8:2281–308. 24157548 10.1038/nprot.2013.143 PMC3969860

[86] Wickham H. ggplot2, elegant graphics for data analysis. 2016. p. 89–107.

[87] Casado P, Rodriguez-Prados J-C, Cosulich SC, Guichard S, Vanhaesebroeck B, Joel S, et al. Kinase-substrate enrichment analysis provides insights into the heterogeneity of signaling pathway activation in leukemia cells. Sci Signal. 2013;6:rs6. 23532336 10.1126/scisignal.2003573

[88] Türei D, Valdeolivas A, Gul L, Palacio-Escat N, Klein M, Ivanova O, et al. Integrated intra- and intercellular signaling knowledge for multicellular omics analysis. Mol Syst Biol. 2021;17:e9923. 33749993 10.15252/msb.20209923 PMC7983032

[89] Kolde R. pheatmap: Pretty Heatmaps. 2019. https://CRAN.R-project.org/package=pheatmap.

[90] Wickham H, Averick M, Bryan J, Chang W, McGowan L, François R, et al. Welcome to the Tidyverse. J Open Source Softw. 2019;4:1686.

[91] Lam F, Lalansingh CM, Babaran HE, Wang Z, Prokopec SD, Fox NS, et al. VennDiagramWeb: a web application for the generation of highly customizable Venn and Euler diagrams. BMC Bioinform. 2016;17:401. 10.1186/s12859-016-1281-5 PMC5048655 27716034

[92] Arafeh R, Shibue T, Dempster JM, Hahn WC, Vazquez F. The present and future of the Cancer Dependency Map. Nat Rev Cancer. 2025;25:59–73. 39468210 10.1038/s41568-024-00763-x

