# Supplementary material for: M1-linked ubiquitination by LUBAC regulates AMPK signalling and the response to energetic stress
Source: Cell Death Differ. 2026 Feb 13;33(8):1685–705. doi: 10.1038/s41418-026-01675-z (PMC13434424; doi:10.1038/s41418-026-01675-z)
Supplement: Supplementary file 1 [file 41418_2026_1675_MOESM1_ESM.pdf]

Supplementary Methods

M1-linked Ubiquitination by LUBAC Regulates AMPK Signalling and the Response to Energetic Stress

Camilla Reiter Elbæk, Sophie Gradinaru, Anna M. Dahlström, Alexander Frueh, Akhee S. Jahan, Joyceline Cuenco, Anna L. Aalto, John Rizk, Simon A. Hawley, Sarah N. J. Franks, Michael Stumpe, Srinivasa Prasad Kolapalli, Chris Kedong Wang, Cara J. Ellison, Ximena Hildebrandt, Klara Nielsen, Dominik Priesmann, Julian Koch, Mathilde Deichmann, Josef Gullmets, Lien Verboom, Geert van Loo, Nieves Peltzer, Lisa B. Frankel, Paul R. Elliott, Mads Gyrd-Hansen, Jörn Dengjel, Brent J. Ryan, D. Grahame Hardie, Annika Meinander, Kei Sakamoto, and Rune Busk Damgaard

Supplementary Methods

Generation of knockout cells using CRISPR-Cas9

For the generation of knockout cells, U2OS, HepG2, or AML12 cells were grown to ~60% confluency and then transfected with HOIP- or OTULIN-targeting gRNA cloned into the pSpCas9(BB)-2A-Puro (PX459) backbone. 48 hours post-transfection, puromycin selection was initiated (5 µg/mL). Following selection, cells were plated into 96-well plates and grown from single clones. Successful knockout was confirmed by immunoblotting. Individual clones were maintained in selection-free media. OTULIN-deficient HepG2 cells were generated using a gRNA designed using the CRISPRscan tool (OTULIN gRNA #2) and cloned into the pSpCas9(BB)-2A-GFP (PX458) vector. Cells were electroporated with plasmid DNA using the NEPA21 cuvette electroporator. Two days post-electroporation, GFP-positive single cells were sorted into 96-well plates. Clones were screened by immunoblotting. gRNA sequences are listed in **Table 1**. PX458 and PX459 were gifts from Professor Feng Zhang (Addgene plasmids #48138 and #48139).

siRNA transfection

Cells were seeded and cultured to ~50-60% confluency before transfection using Lipofectamine® RNAiMAX Transfection Reagent (Thermo Fisher Scientific) following the manufacturer's instructions. The final concentration of siRNA was either 40 nm (for siOTULIN and siHOIP) or 50 nM (siLUBAC: 25 nM siHOIP + 12.5 nM siHOIL-1 + 12.5 nM siSHARPIN). Non-targeting siRNA (siCtrl) was used as control at the same concentrations. Oligo sequences are listed in **Table 1**. After overnight incubation, the transfected cells were reseeded and allowed to grow for an additional 24 hours before they were used in experiments.

Isolation of primary hepatocytes

Murine hepatocytes were isolated as described previously[65] with some modifications. Briefly, the liver was perfused with washing buffer (10 mM HEPES and 200 µM EGTA in HBSS without calcium or magnesium (Gibco, 14170112)) through the vena porta at a flow rate of 5 mL/min. The vena cava was cut to allow drainage. This was followed by perfusion of digestion buffer containing collagenase from *Clostridium histolyticum* (Sigma, C5138) in HBSS with calcium and magnesium (Gibco, 24020117). The liver was then excised and transferred to a dish containing ice-cold plating medium (Medium-199 (Gibco, 31150-022) + 10% FBS, 1% (v/v) PS), and the gallbladder was removed. The resulting cell suspension

was filtered through a 100 µm filter, centrifuged, and cells washed in plating medium. Viable hepatocytes were purified by centrifugation in 90% Percoll (Sigma P4937) in PBS. Live cells were washed in plating medium once more before being passed through a 70 µm filter. Finally, the cells were diluted in plating medium supplemented with 0.1 µM dexamethasone (Sigma, D4902) and 1 nM insulin (Sigma, I9278). Hepatocytes were seeded onto collagen-coated plates at a density of 200,000 cells/mL and allowed to attach for 2-4 hours before changing to culture medium (low-glucose DMEM; Gibco, 10567014; with 10% FBS and 1% PS). The following day, the hepatocytes were used for experiments. Plating medium with glucose-free DMEM was used for hepatocyte starvation (Gibco, 11966025).

Cell and tissue lysis and protein extraction for immunoblotting

Cells were scraped into lysis buffer (25 mM HEPES (pH 7.4), 150 mM KCl, 2 mM $MgCl_2$, 1 mM EGTA, 0.5% Triton X-100) supplemented with cOmplete™ EDTA-free Protease Inhibitor Cocktail (Merck), PhosSTOP™ (Merck), 5 mM N-Ethylmaleimide (NEM), and 1 mM dithiothreitol (DTT). Lysates were cleared by centrifugation for 10 minutes at 13,000 rcf at 4°C. Protein concentration was measured with a Bradford assay and concentrations were adjusted accordingly to ensure equal loading.

Inguinal subcutaneous white fat pads were harvested from mice and snap-frozen in liquid nitrogen. Frozen samples were cut on dry ice and placed in 2 mL round-bottom Eppendorf tubes containing stainless steel beads. In the first step, lysis buffer (50 mM Tris (pH 7.5), 150 mM NaCl, without detergent) supplemented with protease inhibitor, phosphatase inhibitor, NEM, and DTT was added at a ratio of 5 µL/mg ensuring a minimum volume of 500 µL per sample. Tissues were homogenized using the TissueLyser II (Qiagen) at 25 Hz until they were fully disrupted and homogeneous. Samples were centrifuged at 6,000 rcf for 15 min at 4°C to separate the lipid layer. The fat cake (white lipid layer) was carefully removed. NP-40 was added to the remaining lysate at 0.5% (v/v) and incubated for 30 min on ice. A final centrifugation at 13,000 rcf for 15 min at 4°C was performed to remove residual lipids.

Whole liver lysates from 9-day-old control and *Otulin*$^{\Delta hep}$ mice were prepared as previously described[23] using a TissueLyser II (Qiagen) to lyse tissue samples in RIPA buffer (50 mM Tris pH 7.4, 1% NP-40, 0.5% deoxycholate, 0.1% SDS, 150 mM NaCl, 2 mM EDTA, and 5 mM $MgCl_2$) supplemented with cOmplete protease inhibitor cocktail and PhosSTOP (Merck).

Adult *Drosophila melanogaster* flies (10 flies/genotype/sample) were homogenized and lysed with an electronic pestle for 15 min in ice-cold lysis buffer (50 mM Tris pH 7.5,

150 mM NaCl, 1% Triton X-100, 1 mM EDTA pH 8.0, and 10% glycerol) supplemented with Pierce Protease Inhibitor (Thermo Fisher Scientific) and the lysates were cleared by centrifugation.

Lysates were mixed with 4x Laemmli sample buffer (LSB) to obtain a 1x concentration (Tris pH 6.8, 10% glycerol (v/v), 100 mM DTT, 2% SDS (w/v), bromophenol blue) and heated for 5 min at 60°C.

Purification of M1-linked Ubiquitin Conjugates

M1-linked Ub conjugates were purified using either the M1-SUB or the M1-Trap (**Table 1**) as previously described[32,66]. Cells were lysed in ice-cold TUBE buffer (20 mM Na_2HPO_4, 20 mM NaH_2PO_4, 1% IGEPAL CA-630 (Sigma), 2 mM EDTA) supplemented with cOmplete™ Protease Inhibitor Cocktail (Merck), PhosSTOP™ (Merck), and 5 mM NEM. Lysates were cleared at 13,000 rcf for 10 minutes at 4˚C and then supplemented with 1 mM DTT and 100-150 µg/mL of purified GST-M1-SUB or GST-M1-Trap and incubated with pre-washed Glutathione Sepharose 4B (Merck) or Glutathione Magnetic Agarose Beads (Merck). Samples were incubated on beads for 2-18 hours at 4°C with gentle agitation, washed with ice-cold PBS-Tween (PBS-T; 0.1% Tween-20 in PBS), and eluted in LSB for immunoblot analysis.

Immunoprecipitation-coupled AMPK kinase assays

Cells were lysed in lysis buffer (50 mM Tris–HCl (pH 7.4), 150 mM NaCl, 1% (v/v) Triton X-100, 50 mM NaF, 5 mM sodium pyrophosphate, 1 mM EDTA, 1 mM EGTA, 1 mM DTT, 0.1 mM benzamidine, 0.1 mM PMSF). Lysates were cleared and protein concentration was determined using Bradford assay. Equal amounts of anti-AMPK-α1 and -α2 antibodies were coupled to protein G-Sepharose beads. The beads were incubated with 100-300 µg of cell lysate for 2 hours at 2-8°C. Beads were washed twice with high salt IP buffer (IP buffer with 1 M NaCl), twice with regular IP buffer, and twice with HEPES assay buffer (50 mM HEPES, 0.02% Brij-35, and 1 mM DTT). Beads were resuspended in HEPES assay buffer and aliquoted to a final volume of 20 µL for kinase assays. AMPK activity was measured based on its ability to phosphorylate the synthetic AMARA peptide (AMARAASAAALARRR) derived from rat ACC[67]. The kinase reaction was initiated by adding 30 µL of HEPES assay buffer containing 200 µM AMP, 200 µM [γ-^{33}P]-ATP (Hartmann Analytic), 5 mM $MgCl_2$, and 200 µM AMARA peptide to reach a final volume of 50 µL. Assays were incubated at 30°C on an orbital shaker for 30-60 min. Reactions were stopped by spotting 30 µL of the mixture

onto P81 filter paper, followed by immersion in 1% orthophosphoric acid. Filter papers were washed with water to remove unincorporated ATP, dried at room temperature, and submersed in scintillation fluid. Radioactivity was measured on a scintillation counter. One unit of AMPK activity was defined as the incorporation of 1 nanomole of ^{33}P into the AMARA peptide mg^{-1} min^{-1}.

Purification of Ubiquitin Affinity reagents

The M1-SUB (GST-NEMO-CoZi-His) and M1-Trap (GST-OTULIN80-348-His; C129A) were purified from E. Coli BL21(DE3). Chemically transformed bacteria in LB-broth were grown to OD 0.6, induced with 0.5 mM IPTG, grown overnight at 18 °C, and then pelleted and flash frozen. Recombinant proteins were purified by IMAC or GST purification. For IMAC, pellets were resuspended in Buffer A (25 mM Tris, 200 mM NaCl, 20 mM Imidazole (pH 8.0)) supplemented with cOmplete™ Protease Inhibitor Cocktail (Merck) and 10 U/ml rDNase I (Roche). After sonication, pelleting (29,000 rcf, 20 min, 4°C) and filtration (1 μm PES), the cleared lysate was loaded on a HisTrap FF Column (Cytiva). Protein was eluted by gradient up to 100% Buffer B (25 mM Tris, 200 mM NaCl, 500 mM Imidazole (pH 8.0)). Protein was further purified and buffer-exchanged into PBS (10 mM Pi, 138 mM NaCl, 2.7 mM KCl (pH 7.4)) on a Superdex 75 Increase 10/300 GL SEC column (Cytiva). For GST purification, pellets were resuspended in PBS supplemented with cOmplete™ Protease Inhibitor Cocktail (Merck) and 10 U/ml rDNase I (Roche) and lysed by sonication. Cleared lysate was incubated with 0.5 mL slurry of Glutathione Sepharose 4B beads (Cytiva) at 4°C for 1 h while tumbling slowly in a 49 mL Econo-Column (Bio-Rad). After releasing the flowthrough, beads were washed with ice cold PBS and eluted via addition of 5 x 1 mL GSH-elution buffer (PBS supplemented with 50 mM GSH and 1 mM DTT). Purified protein was buffer-exchanged into PBS via NAP-25 columns (Cytiva).

Purification of recombinant E1, E2, and LUBAC

E1 (human UBA1) was cloned into a modified pACEBac-1 vector encoding an N-terminal Twin Strep tag, mScarlet, and a 3C protease site. Individual human LUBAC components were cloned into pLIB vectors with an N-terminal Twin Strep tag, followed by mScarlet, and a 3C protease site on HOIP. HOIL-1 and SHARPIN contained no affinity tags. A single co-expressing LUBAC complex was generated using the pBIG cloning strategy[68]. Plasmids were transformed into DH10Bac cells (Invitrogen). Bacmids were isolated and transfected into Sf9 cells to produce baculovirus followed by amplification in a larger volume of Sf9 cells for infection of at least 1 L of Sf9 cells. Cells were harvested 72 hours post-infection and

lysed in 20 mM Tris pH 8.5, 200 mM NaCl, 4 mM DTT, 10% (v/v) glycerol, 0.5% (v/v) Tween-20, cOmplete protease inhibitor (Merck), and 1 mM PMSF, then clarified and applied to Strep-Tactin resin (IBA). Recombinant protein was eluted by incubation with 3C protease and resin was washed with 20 mM Tris pH 8.5, 4 mM DTT followed by purification by anion exchange chromatography (Resource Q, Cytiva) and size exclusion chromatography (Superdex 200, Cytiva) in 20 mM HEPES pH 7.5, 200 mM NaCl, 4 mM DTT. E2 (human UBE2D2) was cloned into a pOPIN B vector and expressed in Rosetta DE3 *E. coli* grown in 2x TY medium. Recombinant protein expression was induced by addition of 0.4 mM IPTG at 18 °C overnight and purified as previously described[69].

Nucleotide extraction and measurement of ATP, ADP and AMP

Culture medium was aspirated from cells, followed by washing in ice-cold PBS and lysis in ice-cold 5% perchloric acid. Samples were vortexed and lysates clarified by centrifugation. The supernatant was collected and an equal volume of 1:1 mixture of tri-n-octylamine and 1,1,2-trichlorotrifluoroethane added. Samples were vortexed, centrifuged, and the aqueous phase collected. This procedure was repeated twice more, and nucleotides stored at -20°C before analysis. All centrifugation steps were performed at 13,000 rcf, 4°C for 3 minutes. The levels of AMP, ADP and ATP were measured using a TSQ Quantiva interfaced with Ultimate 3000 Liquid Chromatography system (Thermo-Scientific), as described previously[70]. The porous graphitic carbon column (Hyper Carb 30x1mm ID 3 mm; Part No: C-35003-031030, Thermo-Scientific) was maintained at a constant temperature of 42˚C and equilibrated for 11 min with 7% buffer B at a constant flow rate 0.045 mL/min. Aliquot of 1 µL of each sample were loaded onto the column and the three nucleotides were eluted with a linear gradient of 7% to 12% buffer B (80% (v/v) acetonitrile) over 3 min, then from 12%-90% buffer B within 2 min and finally from 90%-100 buffer B within 4 min. The area of each nucleotide was determined by manually integrating area under the peak. The relative abundances of AMP, ADP and ATP were converted to adenylate energy charge (AEC) defined as:

$$AEC = \frac{[ATP]\times 0.5[ADP]}{[ATP]+[ADP]+[AMP]}$$

Proteomics

Sample preparation:

Immunoprecipitated proteins were digested on-beads by eluting in 20 µL of MS lysis buffer (6 M Guanidinium-HCl, 10 mM TCEP, 40 mM CAA, 50 mM HEPES, pH 8.5). The samples were heated to 95°C for 5 minutes, followed by sonication at high intensity for 5 cycles of 30 seconds in a Bioruptor sonication water bath (Diagenode) at 4°C. 500 µg of protein was used for digestion. Samples were diluted 1:3 with 10% acetonitrile in 50 mM HEPES (pH 8.5). Proteins were first digested with LysC (MS grade, Wako) at a 1:50 enzyme-to-protein ratio and incubated at 37˚C for 4 hours. The samples were further diluted 1:10 with 10% acetonitrile in 50 mM HEPES (pH 8.5), and trypsin (MS grade, Promega) was added at a 1:100 enzyme-to-protein ratio for overnight digestion at 37˚C. Digestion was quenched by adding 2% trifluoroacetic acid (TFA) to a final concentration of 1%. Peptides were desalted using a SOLAµ™ SPE Plate (Thermo Fisher) according to the manufacturer's instructions. The eluted peptides were then concentrated using an Eppendorf Speedvac and reconstituted in Buffer A* (0.1% formic acid + iRT peptides (Biognosys, KI-3002-2)). A total of 500 ng of peptides were loaded onto the instruments for analysis.

MS data acquisition:

Peptides were loaded onto a 2 cm C18 trap column (ThermoFisher, 164946), connected in-line to a 15 cm C18 reverse-phase analytical column (Thermo EasySpray, ES904) using 100% Buffer A at 750 bar, using the Thermo EasyLC 1200 HPLC system. Peptides were eluted over a 70 minute gradient ranging from 6 to 60% of Buffer B at 250 nL/min, and the Q Exactive instrument (Thermo Fisher Scientific) was run in a DD-MS2 top10 method. Full MS spectra were collected at a resolution of 70,000, with an AGC target of 3×10^6 or maximum injection time of 20 ms and a scan range of 300–1750 m/z. The MS2 spectra were obtained at a resolution of 17,500, with an AGC target value of 1×10^6 or maximum injection time of 60 ms, a normalised collision energy of 25 and an intensity threshold of 1.7e4. Dynamic exclusion was set to 60 s, and ions with a charge state <2 or unknown were excluded. MS performance was verified for consistency by running complex cell lysate quality control standards, and chromatography was monitored to check for reproducibility.

Data analysis:

All raw LC-MS/MS data files were processed together using Proteome Discoverer version 2.4 (Thermo Fisher). In the processing step, Oxidation (M), Acetylation (K), and protein N-termini acetylation and met-loss were set as dynamic modifications. All results were filtered with 0.05 delta Cn for PSMs. SequestHT was used as database, matching spectra against the *Homo sapiens* database from Uniprot together with recombinant sequence. Identified

proteins and their quantified abundances were imported into RStudio software (Posit, version 2024.09.0+375) and filtered for missing values, retaining proteins detected in at least 75% of bait samples (with no filtering on empty vector control samples). Abundances were normalised to total sample loading by scaling each sample (column) so that its total protein intensity matched the median total intensity across all samples. After log2 transformation, missing values were imputed for "clean" hits using normal downshift (width = 0.3, downshift = 1.8). “Clean” hits are defined as those not detected in any replicate of control pulldowns but detected in ≥75% of replicates in bait pulldown. Differentially enriched proteins were tested using limma linear modelling[71] applying a fold-change cutoff of 2.5 and a false discovery rate (FDR) below 0.05 after Benjamini-Hochberg (BH)-adjustment for multiple testing. Proteins belonging to the KEGG pathway "hsa04152_AMPK_signaling_pathway" were extracted using the ‘EnrichmentBrowser’ package (version 2.34.1)[72].

Phosphoproteomics

Sample preparation:

Proteins were extracted by lysing cells in 6 M GdnHCl in 250 mM HEPES (pH 8.0) using a bead beater, reduced with 15 mM TCEP for 1h at 55°C, and alkylated with 30 mM IAA for 30 min at room temperature. After acetone precipitation, proteins were resuspended in a small volume of 2.5 M GdnHCl in 100 mM HEPES (pH 8.0) and 120 µg protein of each sample was digested overnight with LysC and trypsin (ratio 50:1, final volume of 100 µL, final GdnHCl concentration of 0.75 M).

Enrichment of phospho-peptides and TMT labelling for proteomics:

TMTpro™ 16-plex labelling was performed according to the manufacturer’s recommendations (Thermo Fisher, A44520). Two TMT 16-plexes were used for the 30 samples, resulting in two batches. After combining the same amount of each TMT label, samples were acidified using 50% TFA (final concentration app. 0.5%, (pH<2.0) and peptides were purified by SPE using HR-X columns in combination with C18 cartridges (Macherey-Nagel). Columns were washed in Buffer A (0.1% formic acid) and eluted in Buffer B (0.1% formic acid in 80% acetonitrile). Eluates were frozen in liquid nitrogen and lyophilized overnight. Peptide mixture was fractionated by high-pH reverse-phase chromatography resulting in eight fractions[73]. Phosphopeptide enrichment was done on each fraction according to Post *et al.*[74] using Fe(III) cartridges on Bravo liquid handling system (Agilent). Flow-through was collected for global proteome analysis.

MS data acquisition:

LC-MS/MS measurements were performed on an Exploris 480 mass spectrometer (Thermo Scientific) coupled to an EasyLC 1200 nanoflow-HPLC (Thermo Scientific). Peptides were separated on a fused silica HPLC-column tip (I.D. 75 μm, New Objective, self-packed with ReproSil-Pur 120 C18-AQ, 1.9 μm (Dr. Maisch) to a length of 20 cm) using a gradient of Buffer A and Buffer B. Samples were loaded with 0% Buffer B with a flow rate of 600 nL/min; peptides were separated by 7%–38% B within 122 min with a flow rate of 250 nL/min. Spray voltage was set to 2.3 kV and the ion-transfer tube temperature to 250°C; no sheath and auxiliary gas were used. Mass spectrometers were operated in the data-dependent mode; after each MS scan (mass range m/z = 370 – 1750; resolution: 120'000) a maximum of twenty MS/MS scans were performed using an isolation window of 0.7, a normalized collision energy of 32%, a target AGC of 50% and a resolution of 45'000. MS raw files were analysed using ProteomeDiscoverer (version 2.5, Thermo Scientific) using a Uniprot full-length *Homo sapiens* database and common contaminants such as keratins and enzymes used for digestion as reference. Carbamidomethyl (C) was set as a fixed modification. For phospho-enriched samples, phosphorylation (S/T/Y), protein N-terminal acetylation, and methionine oxidation were specified as variable modifications. The MS/MS tolerance was set to 0.6 Da and three missed cleavages were allowed using trypsin/P as enzyme specificity.

Data analysis:

Identified peptides and proteins, along with their quantified abundances from Proteome Discoverer, were imported into R software (Posit). The intensities of phosphosites on peptides were summed, accounting for different phosphorylation multiplicities. The datasets were filtered for missing values to retain only the peptides and proteins detected in all samples (across the two TMTplexes). Protein group abundances were used for the proteome analysis, while phosphopeptide abundances were used for the phosphoproteome. Data normalization was carried out in three steps for both proteome and phosphoproteome datasets. First, sample loading was adjusted by multiplying the abundances by a sample-specific scaling factor. Subsequently, artificial internal reference standards were generated to create protein/peptide-specific scaling factors for the two TMTplex batches. Finally, phosphosite abundances were corrected by multiplying them with their corresponding protein abundance scaling factor. In the case of phosphosites without a corresponding protein value, the scaling factor was set to 1. After log2 transformation, differentially regulated phosphosites were tested by limma linear modelling[75], applying a fold-change cutoff of 1.52 (log2-FC=0.6) and a BH-adjusted false discovery rate (FDR) below 0.05. Heatmap was generated using the pheatmap package in R to visualise normalised

phosphosite intensities. To identify patterns of phosphorylation dynamics, fuzzy c-means clustering was employed using the Mfuzz R package[76]. Before clustering, normalised phosphosite abundances were scaled. Clustering was conducted with optimal parameters with c = 4 and m = 1.99, which were determined using the built-in functions of the package. Kinase-substrate enrichment analysis was conducted using the KSEA App R package[77] with annotations from both PhosphoSitePlus (July 2016 release) and NetworKIN with a minimum NetworKIN score of 10 and otherwise default parameters. For functional enrichment, relevant phosphosites were collapsed to their UNIPROT protein ID and sorted by fold-change in descending order. Gene-Set Enrichment Analysis (GSEA) was performed using the clusterProfiler R package[78]. The human proteome served as the background for the representation analysis. KEGG pathway gene sets were tested for enrichment with a BH-adjusted p-value cutoff of <0.05 or 0.01 as indicated.

References (see also reference list in main text)

32 Fiil BK, Damgaard RB, Wagner SA, Keusekotten K, Fritsch M, Bekker-Jensen S *et al.* OTULIN Restricts Met1-Linked Ubiquitination to Control Innate Immune Signaling. *Mol Cell* 2013; **50**: 818–830.

65 Gandin V, Miluzio A, Barbieri AM, Beugnet A, Kiyokawa H, Marchisio PC *et al.* Eukaryotic initiation factor 6 is rate-limiting in translation, growth and transformation. *Nature* 2008; **455**: 684–688.

66 Hrdinka M, Fiil BK, Zucca M, Leske D, Bagola K, Yabal M *et al.* CYLD Limits Lys63- and Met1-Linked Ubiquitin at Receptor Complexes to Regulate Innate Immune Signaling. *Cell Reports* 2016; **14**: 2846–2858.

67 Dale S, Wilson WA, Edelman AM, Hardie DG. Similar substrate recognition motifs for mammalian AMP-activated protein kinase, higher plant HMG-CoA reductase kinase-A, yeast SNF1, and mammalian calmodulin-dependent protein kinase I. *FEBS Lett* 1995; **361**: 191–195.

68 Weissmann F, Petzold G, VanderLinden R, Veld PJH in ’t, Brown NG, Lampert F *et al.* biGBac enables rapid gene assembly for the expression of large multisubunit protein complexes. *Proc Natl Acad Sci* 2016; **113**: E2564–E2569.

69 Dietz L, Ellison CJ, Riechmann C, Cassidy CK, Felfoldi FD, Pinto-Fernández A *et al.* Structural basis for SMAC-mediated antagonism of caspase inhibition by the giant ubiquitin ligase BIRC6. *Science* 2023; **379**: 1112–1117.

70 Ross FA, Hawley SA, Auciello FR, Gowans GJ, Atrih A, Lamont DJ *et al.* Mechanisms of Paradoxical Activation of AMPK by the Kinase Inhibitors SU6656 and Sorafenib. *Cell Chem Biol* 2017; **24**: 813-824.e4.

71 Ritchie ME, Phipson B, Wu D, Hu Y, Law CW, Shi W *et al.* limma powers differential expression analyses for RNA-sequencing and microarray studies. *Nucleic Acids Res* 2019; **43**: e47–e47.

72 Geistlinger L, Csaba G, Zimmer R. Bioconductor's EnrichmentBrowser: seamless navigation through combined results of set- & network-based enrichment analysis. *BMC Bioinform* 2016; **17**: 45.

73 Batth TS, Francavilla C, Olsen JV. Off-Line High-pH Reversed-Phase Fractionation for In-Depth Phosphoproteomics. *J Proteome Res* 2014; **13**: 6176–6186.

74 Post H, Penning R, Fitzpatrick MA, Garrigues LB, Wu W, MacGillavry HD *et al.* Robust, Sensitive, and Automated Phosphopeptide Enrichment Optimized for Low Sample Amounts Applied to Primary Hippocampal Neurons. *J Proteome Res* 2017; **16**: 728–737.

75 Ting L, Cowley MJ, Hoon SL, Guilhaus M, Raftery MJ, Cavicchioli R. Normalization and Statistical Analysis of Quantitative Proteomics Data Generated by Metabolic Labeling*. *Mol Cell Proteomics* 2009; **8**: 2227–2242.

76 Kumar L, Futschik ME. Mfuzz: A software package for soft clustering of microarray data. *Bioinformation* 2007; **2**: 5–7.

77 Wu T, Hu E, Xu S, Chen M, Guo P, Dai Z *et al.* clusterProfiler 4.0: A universal enrichment tool for interpreting omics data. *Innov* 2021; **2**: 100141.

78 Wiredja DD, Koyutürk M, Chance MR. The KSEA App: a web-based tool for kinase activity inference from quantitative phosphoproteomics. *Bioinformatics* 2017; **33**: 3489–3491.
