# Supplementary material for: M1-linked ubiquitination by LUBAC regulates AMPK signalling and the response to energetic stress
Source: Cell Death Differ. 2026 Feb 13;33(8):1685–705. doi: 10.1038/s41418-026-01675-z (PMC13434424; doi:10.1038/s41418-026-01675-z)
Supplement: Supplementary file 2 [file 41418_2026_1675_MOESM2_ESM.pdf]

Supplementary Figures and Legends

M1-linked Ubiquitination by LUBAC Regulates AMPK Signalling and the Response to Energetic Stress

Camilla Reiter Elbæk, Sophie Gradinaru, Anna M. Dahlström, Alexander Frueh, Akhee S. Jahan, Joyceline Cuenco, Anna L. Aalto, John Rizk, Simon A. Hawley, Sarah N. J. Franks, Michael Stumpe, Srinivasa Prasad Kolapalli, Chris Kedong Wang, Cara J. Ellison, Ximena Hildebrandt, Klara Nielsen, Dominik Priesmann, Julian Koch, Mathilde Deichmann, Josef Gullmets, Lien Verboom, Geert van Loo, Nieves Peltzer, Lisa B. Frankel, Paul R. Elliott, Mads Gyrd-Hansen, Jörn Dengjel, Brent J. Ryan, D. Grahame Hardie, Annika Meinander, Kei Sakamoto, and Rune Busk Damgaard

Supplementary Figures S1-S7
Legends Figures S1-S7

Supplementary Figure 1

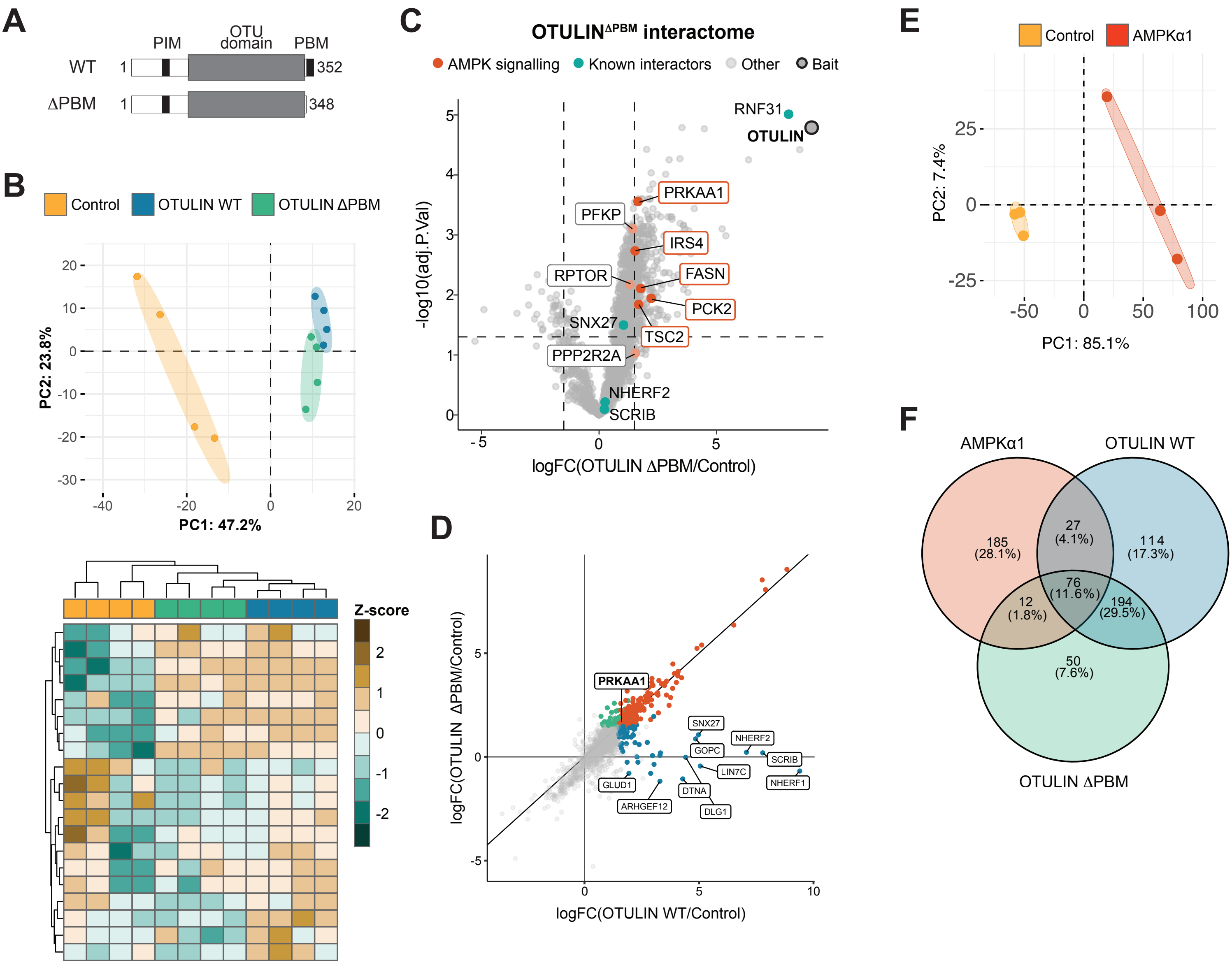

Supplementary Figure 2

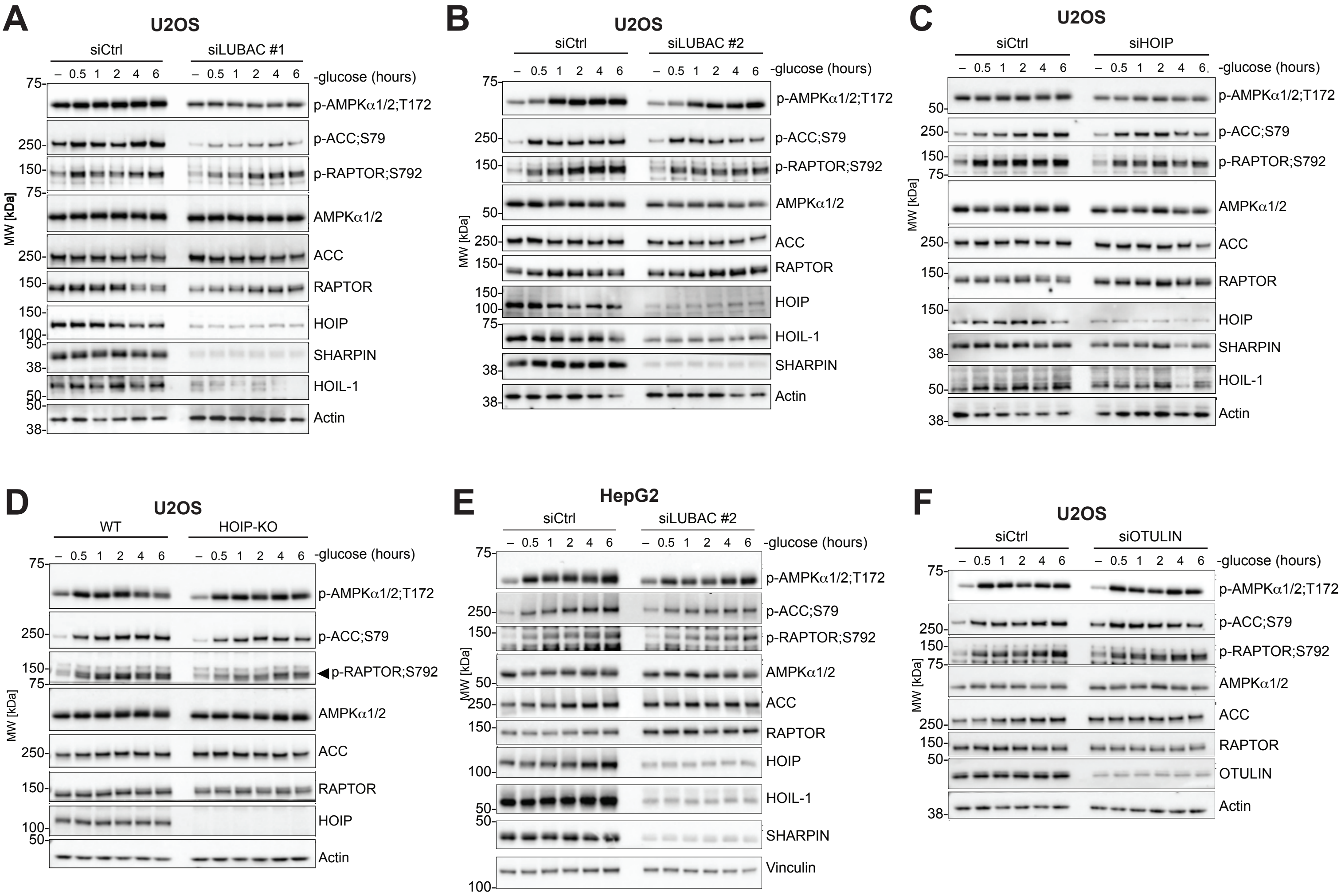

Supplementary Figure 3

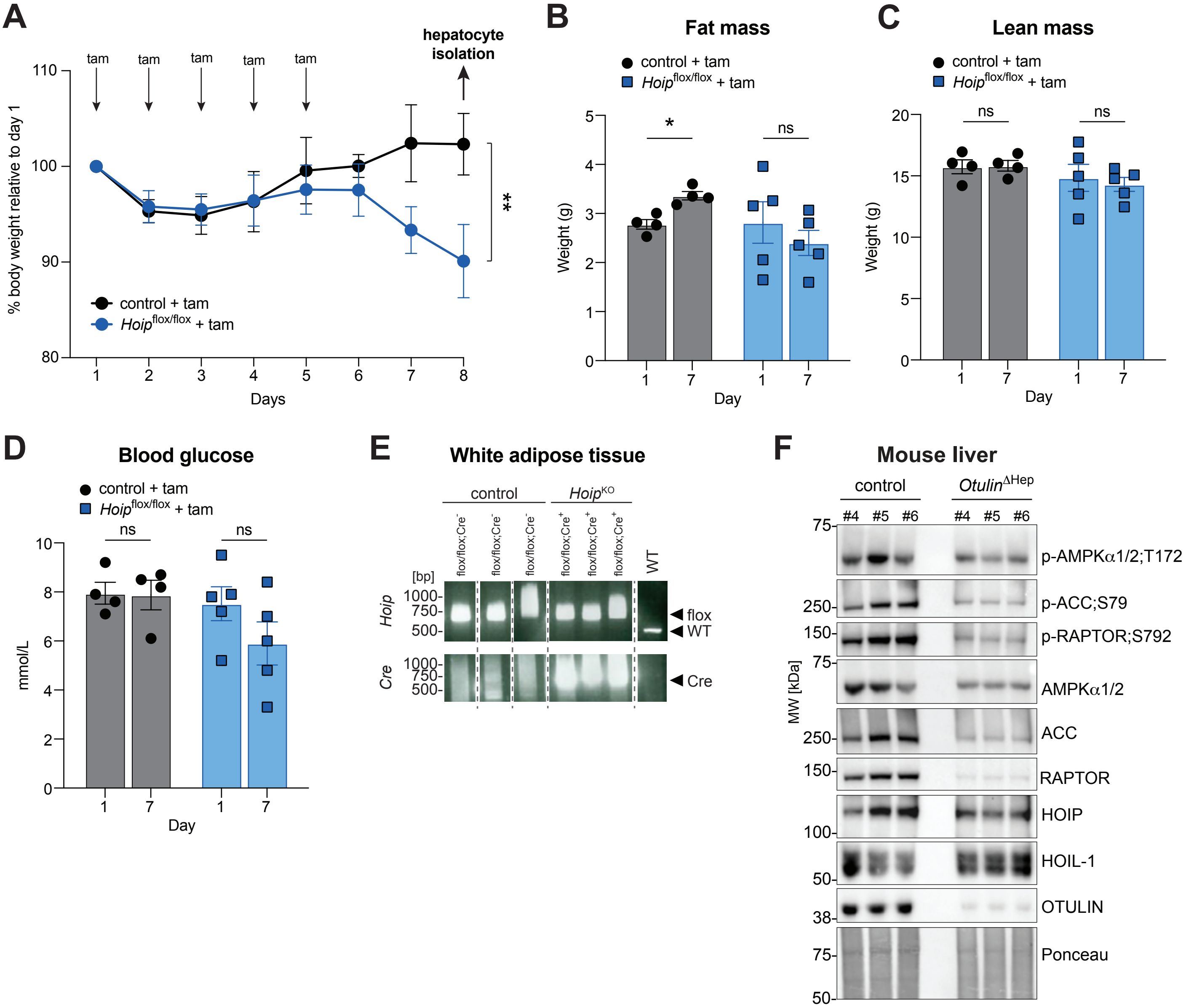

Supplementary Figure 4

A

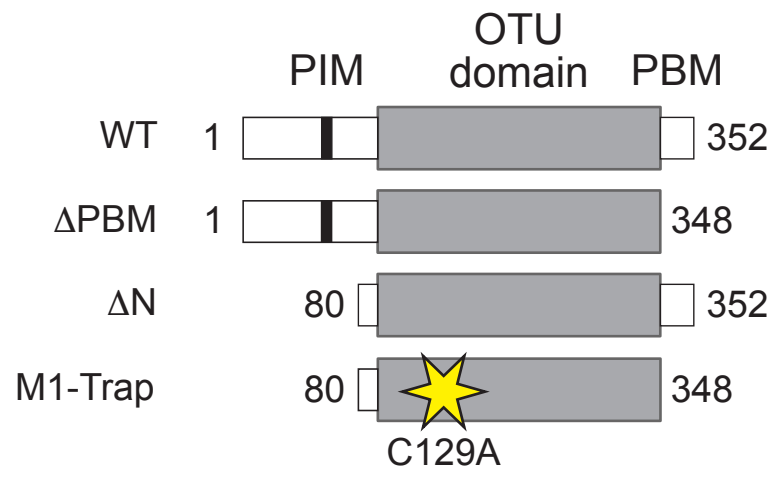

B

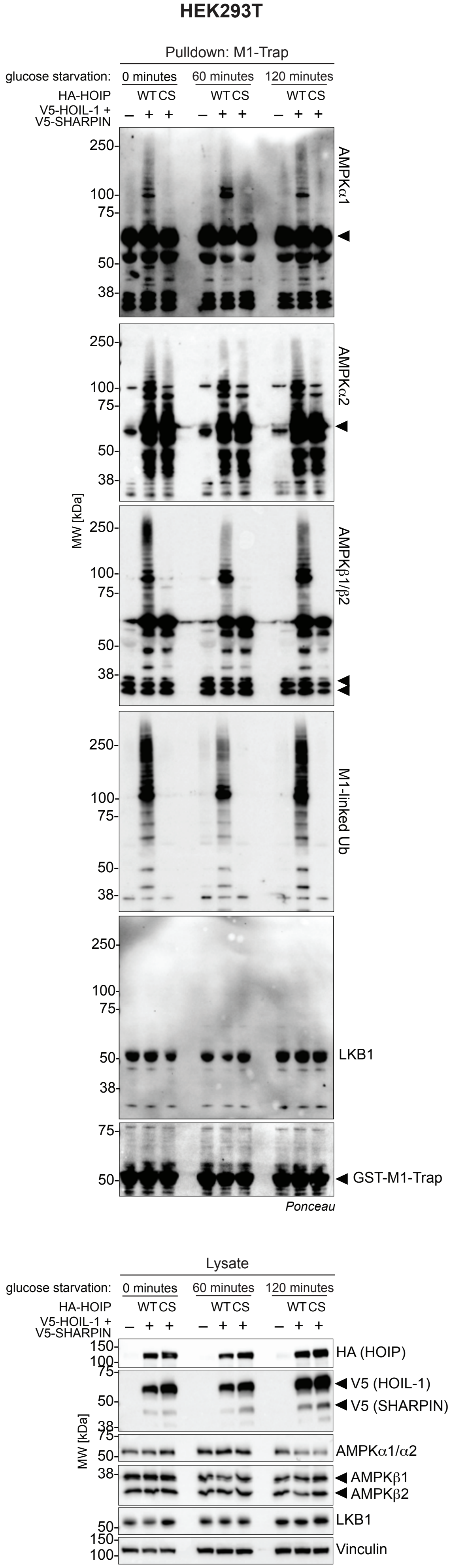

Supplementary Figure 5

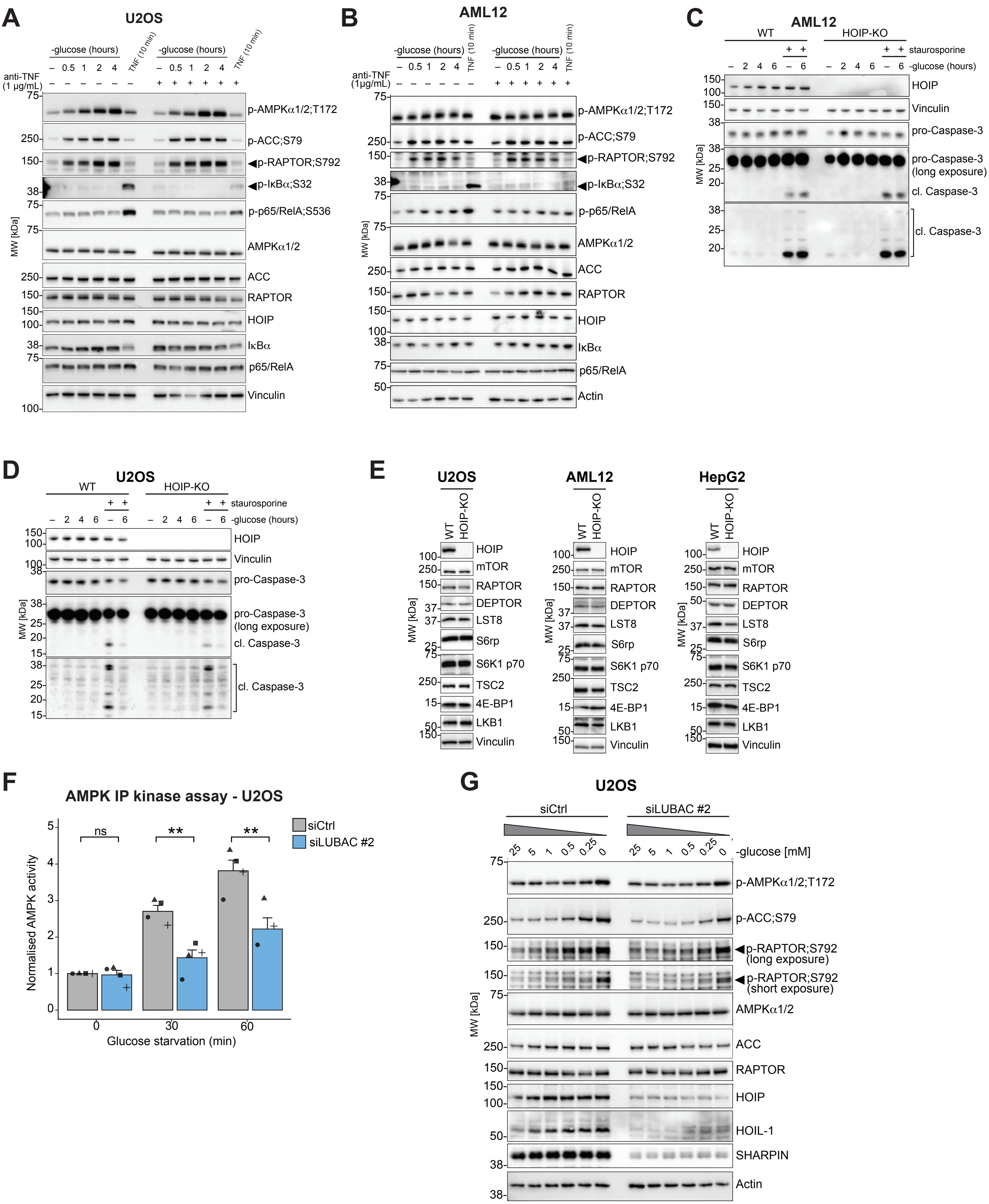

Supplementary Figure 6

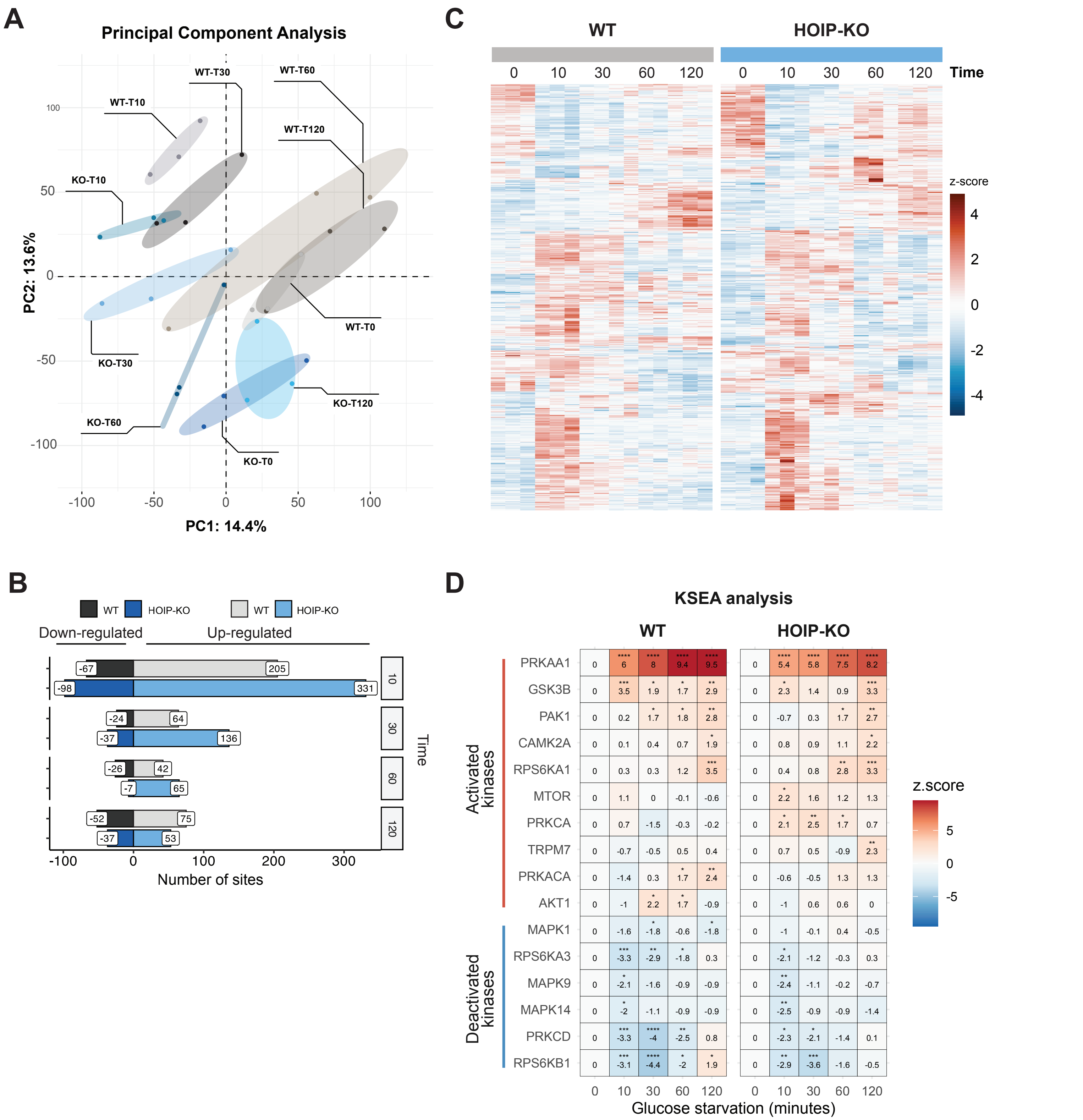

Supplementary Figure 7

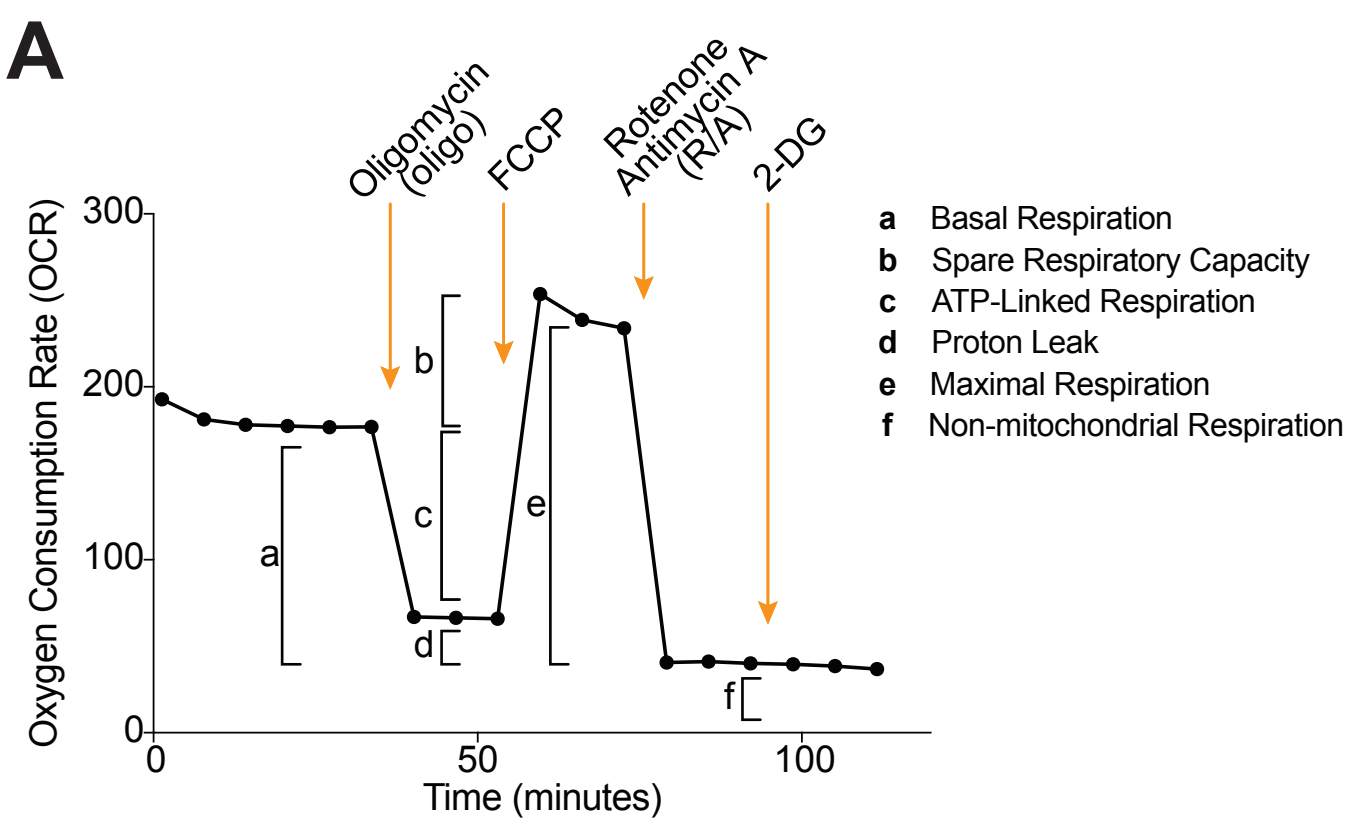

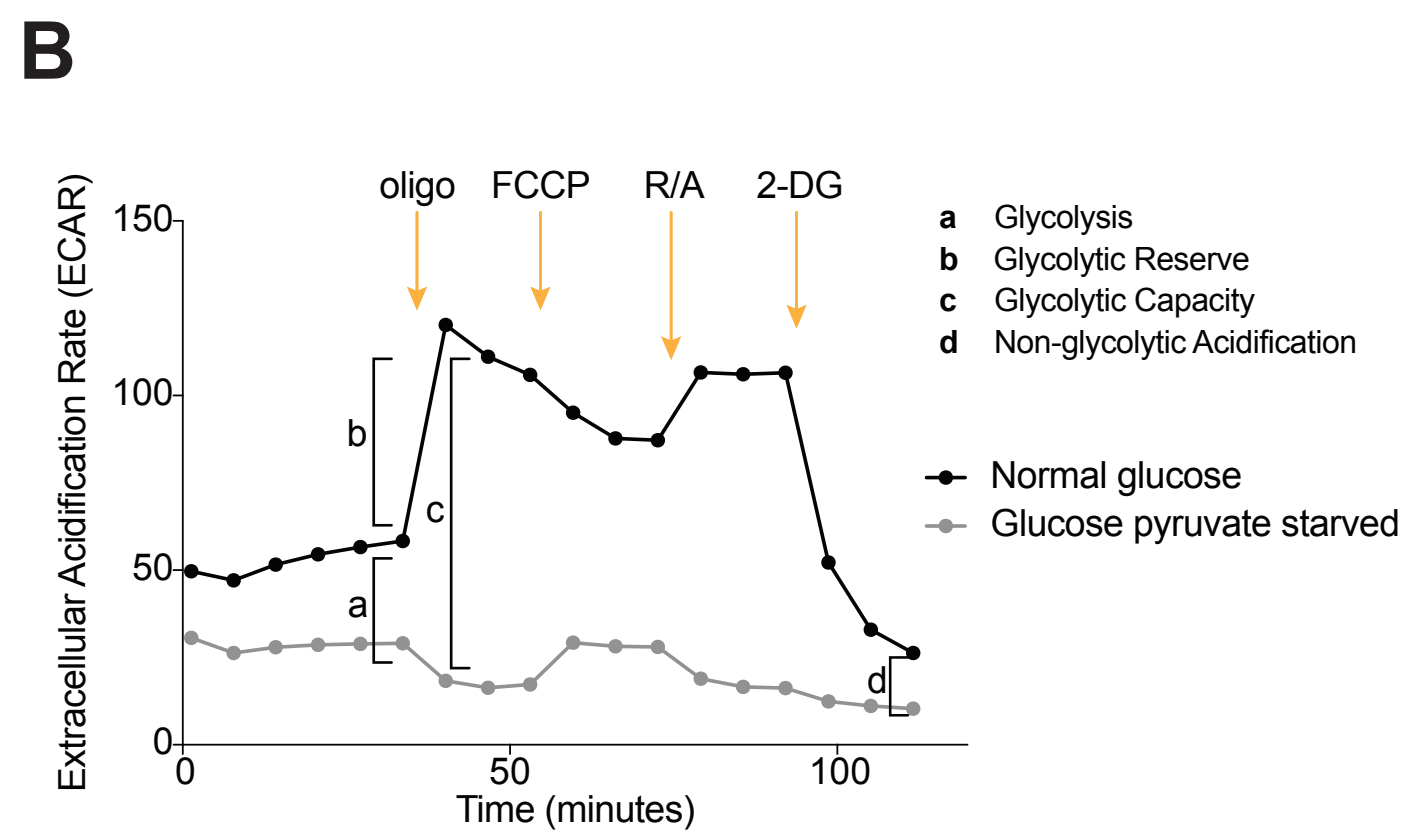

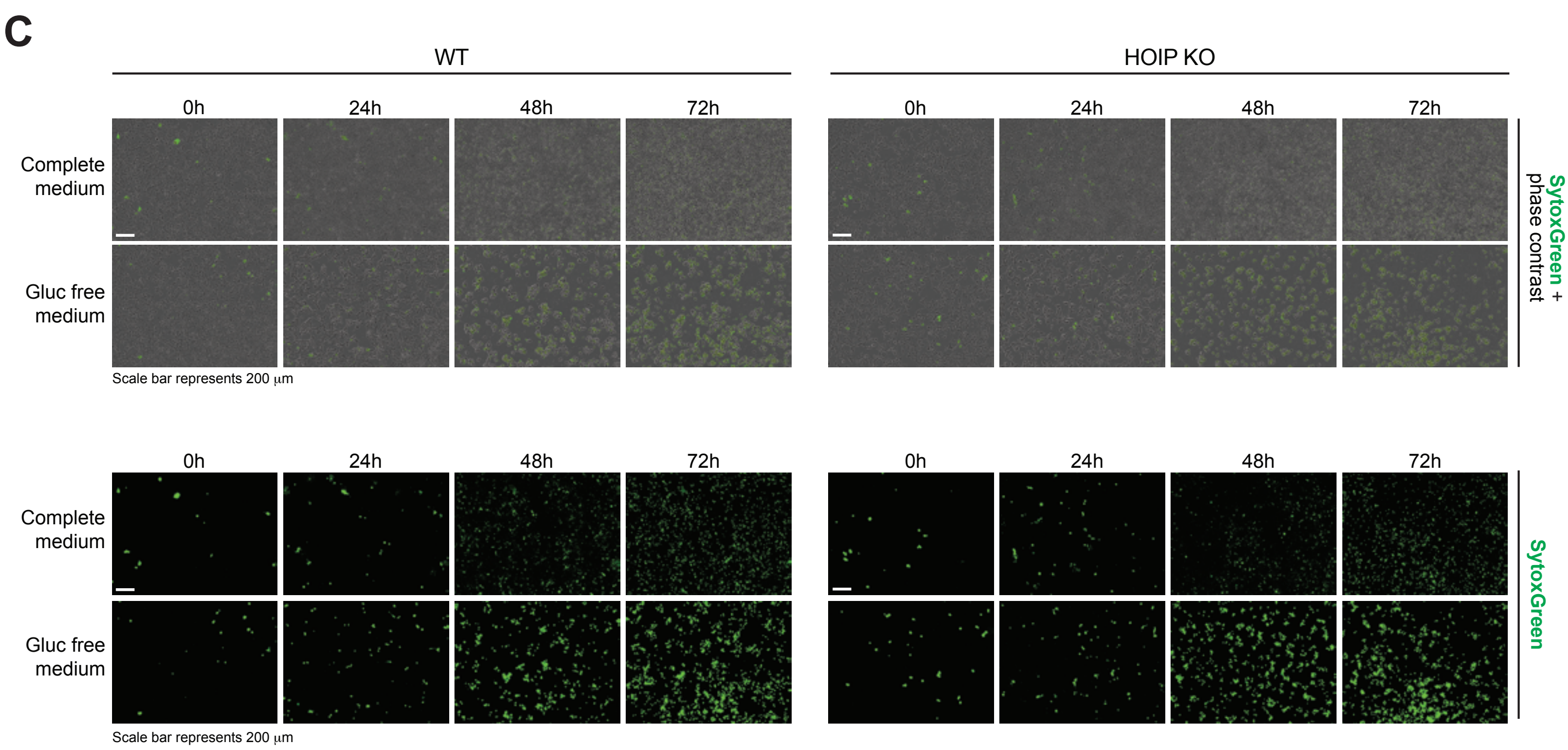

Supplementary Figure Legends

Supplementary Figure S1. Relating to Figure 1

A) Schematic representation of OTULIN wild-type (WT) and PDZ domain-binding motif deletion mutant (ΔPBM). **B**) Top: Principal Component Analysis (PCA) of Strep-pulldown mass spectrometry from HEK293T cells expressing empty vector (Control), OTULIN WT, or OTULIN ΔPBM. PC1 and PC2 explain 71% of total variance. Bottom: Heatmap of normalised protein intensities, clustered using Ward's D2 linkage on Jaccard distances. Proteins were grouped into 20 clusters using k-means clustering. **C**) Volcano plot showing log2 fold-changes (logFC) in OTULIN-ΔPBM pulldown vs. vector control. Known OTULIN interactors (teal) and AMPK pathway proteins (orange) are highlighted. BH-adjusted FDR <0.05; FC cutoff 2.5. **D**) Biplot of logFC for OTULIN-ΔPBM vs. control against OTULIN-WT vs. control. Proteins selectively enriched in WT are labelled. BH-adjusted FDR <0.05; FC cutoff 3.7. **E**) PCA of AMPKα1 pulldown vs. vector control. PC1 and PC2 explain 92.5% of total variance. **F**) Venn diagram showing overlapping proteins between OTULIN WT, OTULIN ΔPBM, and AMPKα1 interactomes. Overlap and percentages indicated.

Supplementary Figure S2. Relating to Figure 2.

A-B) Immunoblots of U2OS cells were transfected with siRNAs targeting HOIP (oligo #1 or #2), HOIL-1, and SHARPIN (LUBAC #1 or #2) or control (siCtrl) and starved in glucose-free medium for the indicated times. **C**) Immunoblots of U2OS cells transfected with siCtrl or siHOIP (oligo #1) alone, starved as in (A). **D**) Immunoblots of U2OS-WT or HOIP-KO cells starved as in (A). **E**) Immunoblots of HepG2 cells transfected with siCtrl or siLUBAC#2, starved as in (A). **F**) Immunoblots of U2OS cells transfected with siCtrl or siRNAs targeting OTULIN, starved as in (A). All blots are representative of three independent experiments.

Supplementary Figure S3. Relating to Figure 3.

A) Body weight (% of day 1) in control and *Hoip*$^{flox/flox}$ 10–14-week-old female mice treated with tamoxifen (tam) for 5 days. Hepatocytes were isolated on day 8. n=6 (control), n=7 (*Hoip*$^{flox/flox}$); ± SEM. Analysed by unpaired Student's t-test on day 8. **B-C**) Fat mass (B) and lean mass (C) measured by MRI on day 1 and 7 after tamoxifen

treatment. Analysed by paired Student's t-test. **D**) Blood glucose levels (mmol/L) on day 1 and 7. Analysed by paired Student's t-test. **E**) Genotyping of subcutaneous adipose tissue from *Hoip*$^{A\text{-}KO}$ mice showing floxed *Hoip* alleles (650 bp band) and Cre recombinase. PCR reactions show the presence of floxed *Hoip* alleles in all animals (lanes 1–6), indicated by a band at the expected size (650 bp). Lanes 4–6 correspond to knockout (KO) animals, which also show the Cre transgene band. Lane 7 shows a WT genotype with *Hoip* at 507 bp and no Cre transgene. **F**) Immunoblot analysis of whole liver lysates from three 9-day-old control and three *Alb*Cre-*Otulin*$^{flox/flox}$ (*Otulin*$^{\Delta Hep}$) mice.

Supplementary Figure S4. Relating to Figure 4.
A) Schematic of OTULIN variants: WT, ΔPBM, ΔN, and M1-Trap. **B**) Immunoblots of endogenous M1-linked Ub conjugates purified from HEK293T cells transfected with empty vector or LUBAC (HOIL-1, SHARPIN, HOIP-WT or C885S) and starved for 0-120 min using recombinant GST-M1-Trap. Blots are representative of three independent experiments.

Supplementary Figure S5. Relating to Figure 5.
A-B) Immunoblots of U2OS (A) and AML12 (B) cells pre-treated with TNF-blocking antibody (anti-TNF) for 60 min and glucose starved or treated with TNF (10 ng/mL, 10 min). **C-D**) AML12 (C) and U2OS (D) WT and HOIP-KO cells were starved with/without 1 µM staurosporine for 6 h as positive control. Caspase 3 cleavage during glucose starvation up to 6 hours was assessed by immunoblotting as a readout of apoptotic cell death. **E**) Immunoblots of mTOR-related proteins in U2OS, AML12, and HepG2 cell lysate **F**) AMPK IP kinase assay with ^{33}P-labelled ATP from from U2OS cells transfected with siCtrl or siLUBAC#2 and starved for glucose as indicated. Bars represent mean ± SEM (n=3). Analysed by two-sided Student's t-test. **G**) Immunoblots of U2OS cells transfected with control siRNA (Ctrl) or siLUBAC#2 and cultured in decreasing glucose concentrations for 60 min. All blots are representative of three independent experiments.

Supplementary Figure S6. Relating to Figure 6.
A) PCA of phosphoproteomics samples: U2OS-WT (grey) and HOIP-KO (blue) cells starved for 0–120 min. PC1 + PC2 explain 28% variance. **B**) Bar plot of significantly

altered phosphosites in U20S-WT and HOIP-KO cells at each time point. Upregulated (right) and downregulated (left) sites shown. FDR < 0.05, FC > 1.5. **C**) Heatmap of normalised phosphosite intensities clustered by correlation. **D**) KSEA-based heatmap of kinase activity scores in U2OS-WT and HOIP-KO cells over time. Higher z-scores reflect greater inferred kinase activity. Top 10 kinases shown and bottom 6 kinases are shown. Significance: ns, *p < 0.05, **p < 0.01, ***p < 0.001, ****p < 0.0001.

Supplementary Figure S7. Relating to Figure 7.

A) Seahorse XF assay: OCR in U2OS-WT and HOIP-KO cells. Basal, maximal, spare respiratory capacity, ATP-linked respiration, and proton leak calculated based on OCR responses to mitochondrial inhibitors (oligomycin, FCCP, rotenone/antimycin A). **B**) ECAR measured in starved cells using Seahorse XF assay. Basal glycolysis, glycolytic capacity, glycolytic reserve, and non-glycolytic acidification calculated following sequential injection of glucose, oligomycin, and 2-DG. **C**) Fluorescence (SytoxGreen) and phase-contrast (confluency) micrographs from IncuCyte analysis of AML12-WT and HOIP-KO cells (Figure 7E). Scale bar = 200 μM.
