# Supplementary material for: M1-linked ubiquitination by LUBAC regulates AMPK signalling and the response to energetic stress
Source: Cell Death Differ. 2026 Feb 13;33(8):1685–705. doi: 10.1038/s41418-026-01675-z (PMC13434424; doi:10.1038/s41418-026-01675-z)

Supplementary Figure 8

M1-linked Ubiquitination by LUBAC Regulates AMPK Signalling and the Response to Energetic Stress

Camilla Reiter Elbæk, Sophie Gradinaru, Anna M. Dahlström, Alexander Frueh, Akhee S. Jahan, Joyceline Cuenco, Anna L. Aalto, John Rizk, Simon A. Hawley, Sarah N. J. Franks, Michael Stumpe, Srinivasa Prasad Kolapalli, Chris Kedong Wang, Cara J. Ellison, Ximena Hildebrandt, Klara Nielsen, Dominik Priesmann, Julian Koch, Mathilde Deichmann, Josef Gullmets, Lien Verboom, Geert van Loo, Nieves Peltzer, Lisa B. Frankel, Paul R. Elliott, Mads Gyrd-Hansen, Jörn Dengjel, Brent J. Ryan, D. Grahame Hardie, Annika Meinander, Kei Sakamoto, and Rune Busk Damgaard

Uncropped immunoblots and images

Figure 1

B

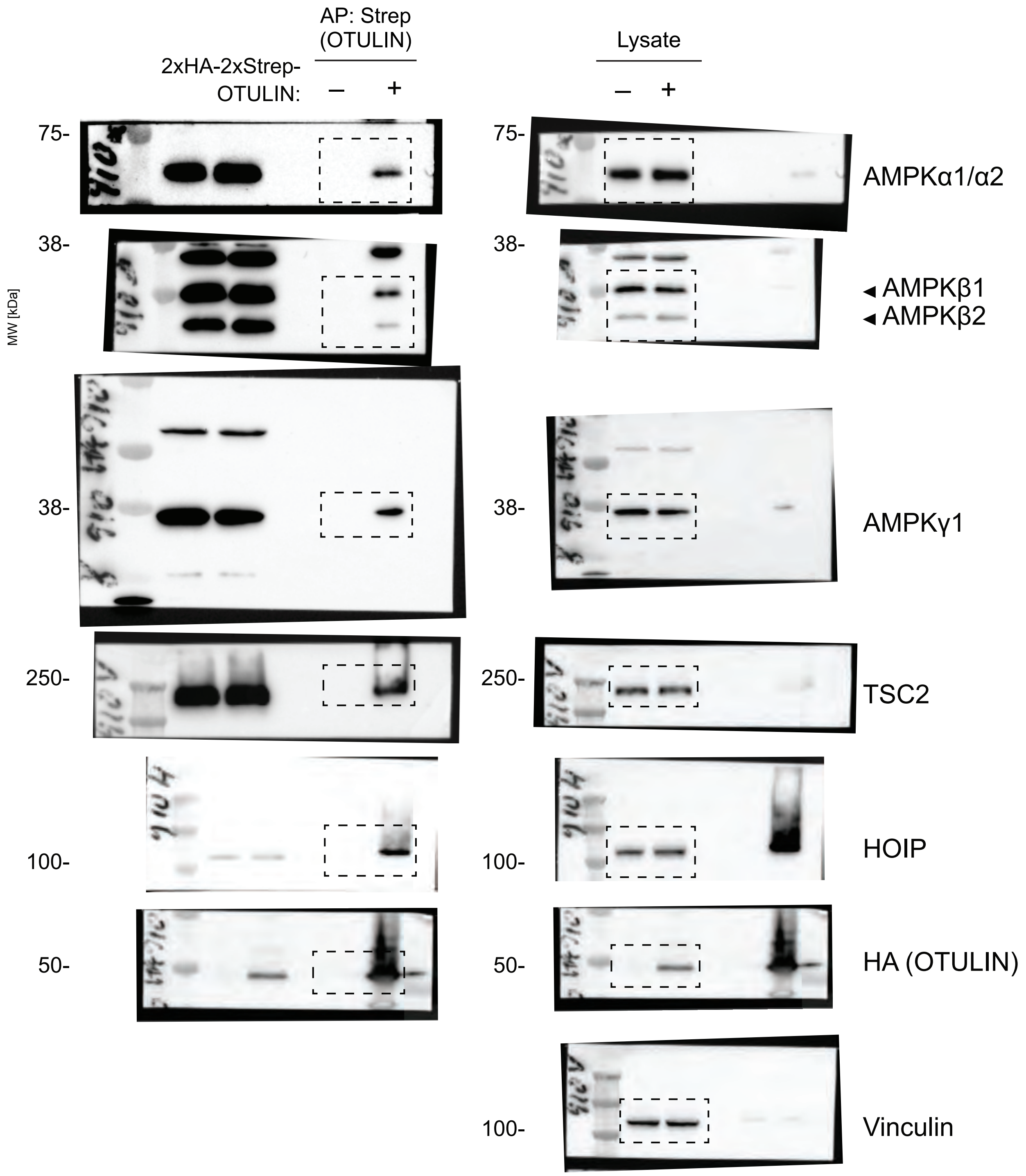
AP: Strep (OTULIN)
Lysate
2xHA-2xStrep-OTULIN:
–
+
–
+
75-
38-
38-
250-
100-
50-
MW [kDa]
75-
38-
38-
250-
100-
50-
100-
AMPKα1/α2
AMPKβ1
AMPKβ2
AMPKγ1
TSC2
HOIP
HA (OTULIN)
Vinculin

Figure 1

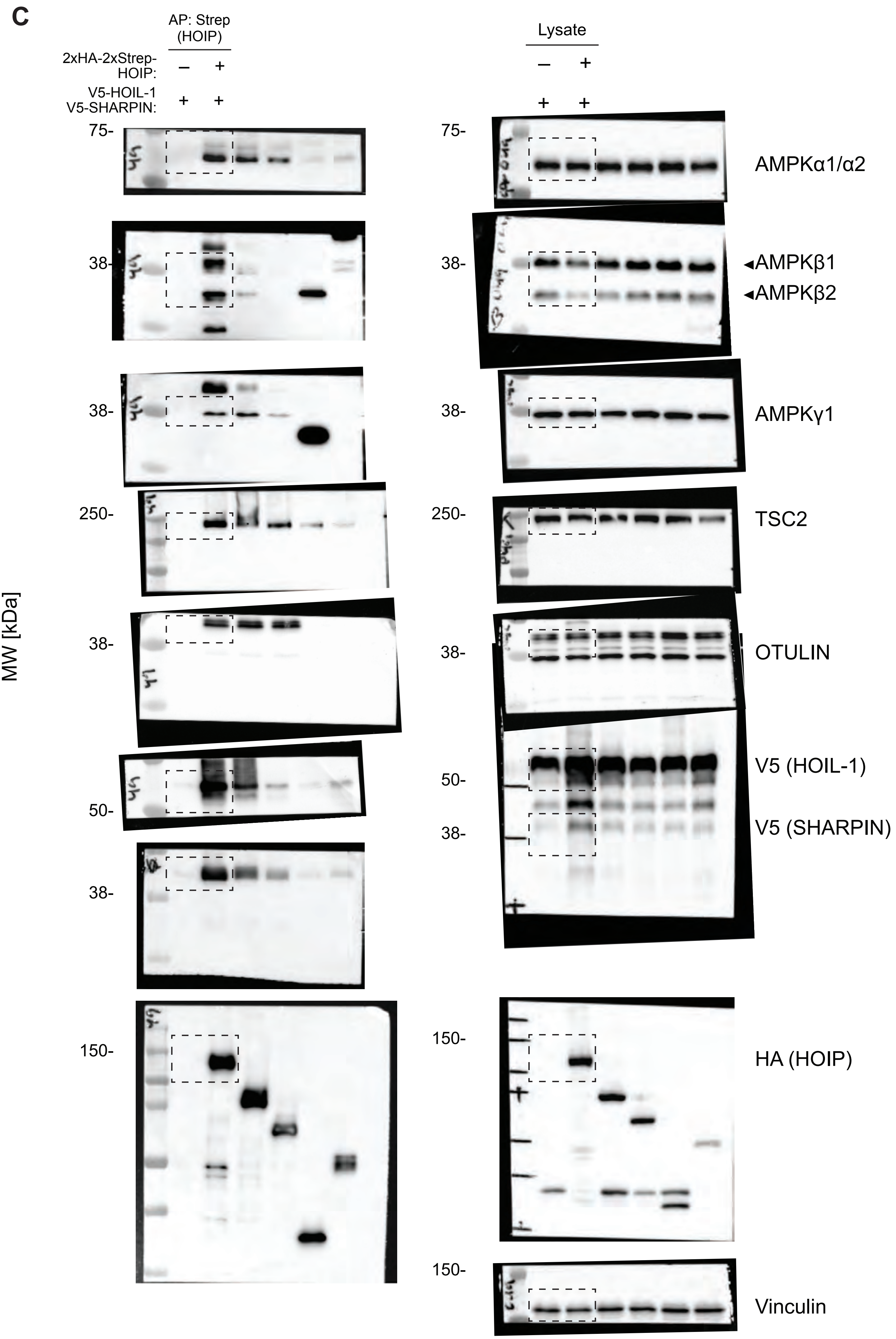
C
AP: Strep (HOIP)
Lysate
2xHA-2xStrep-HOIP:
–
+
V5-HOIL-1
V5-SHARPIN:
+
+
75-
38-
250-
50-
150-
MW [kDa]
AMPKα1/α2
◂AMPKβ1
◂AMPKβ2
AMPKγ1
TSC2
OTULIN
V5 (HOIL-1)
V5 (SHARPIN)
HA (HOIP)
Vinculin

Figure 1

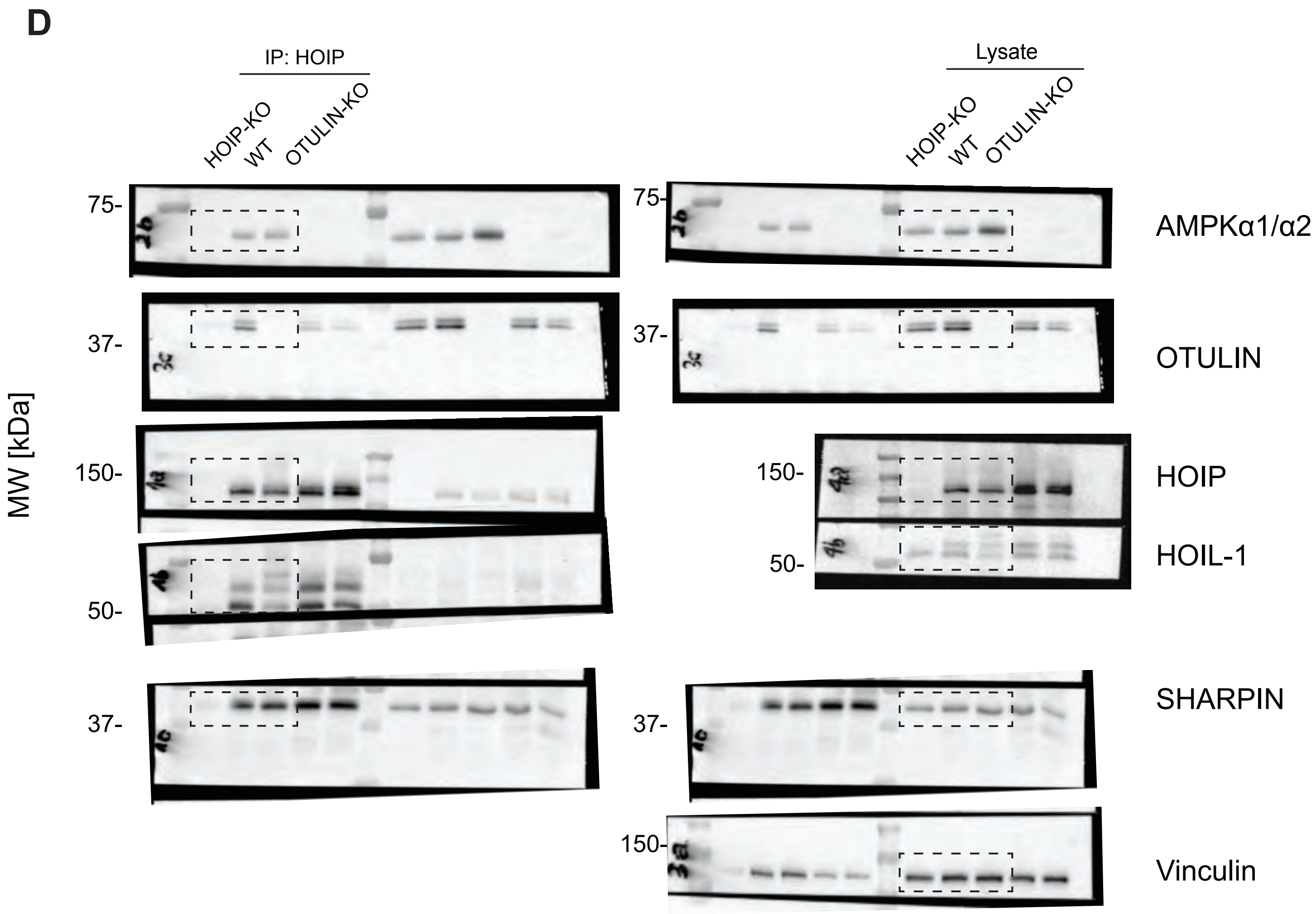
D
IP: HOIP
Lysate
HOIP-KO
WT
OTULIN-KO
HOIP-KO
WT
OTULIN-KO
MW [kDa]
75-
37-
150-
50-
37-
75-
37-
150-
50-
37-
150-
AMPKα1/α2
OTULIN
HOIP
HOIL-1
SHARPIN
Vinculin

Figure 1

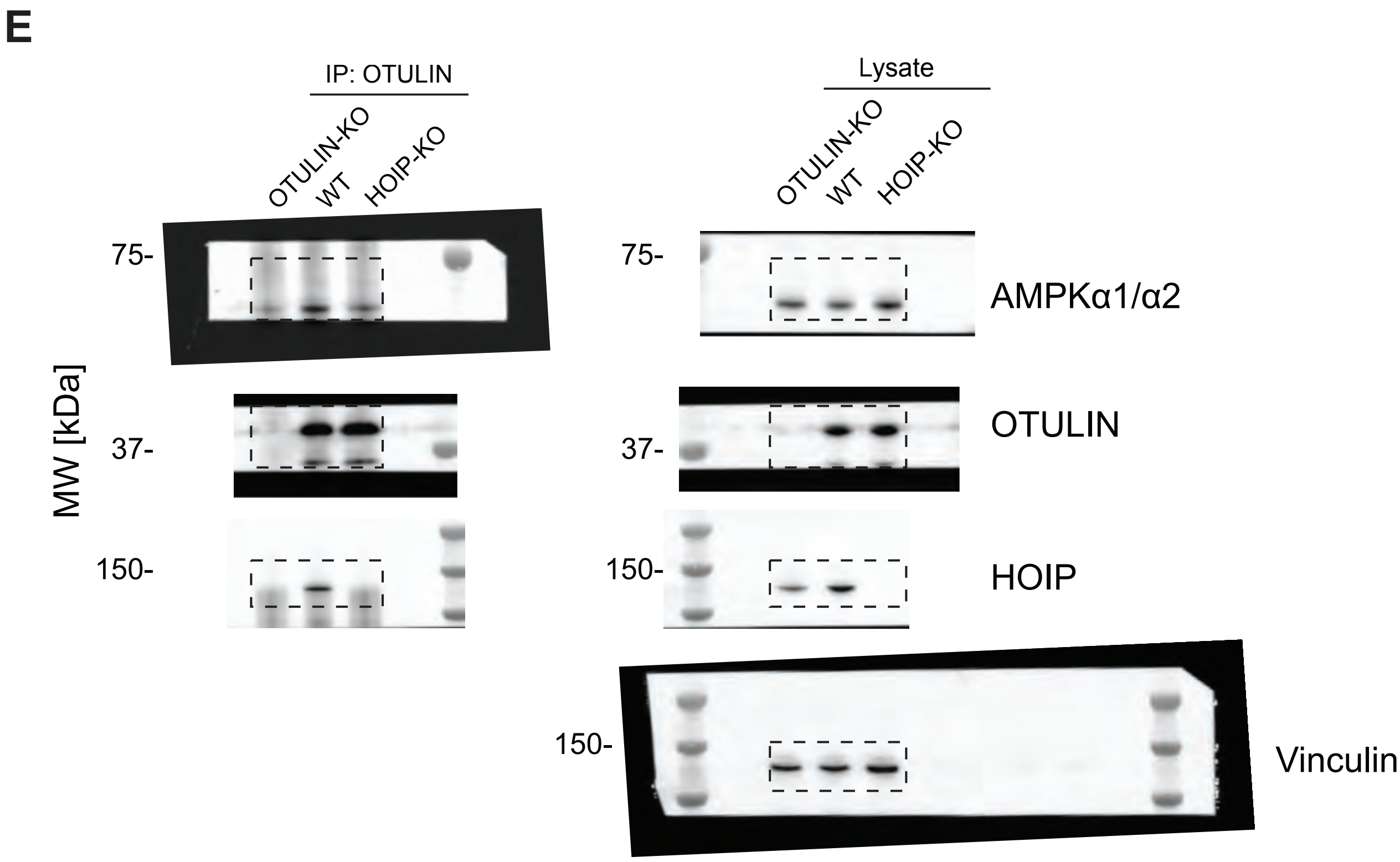
E
IP: OTULIN
Lysate
OTULIN-KO
WT
HOIP-KO
OTULIN-KO
WT
HOIP-KO
75-
75-
AMPKα1/α2
MW [kDa]
37-
37-
OTULIN
150-
150-
HOIP
150-
Vinculin

Figure 1

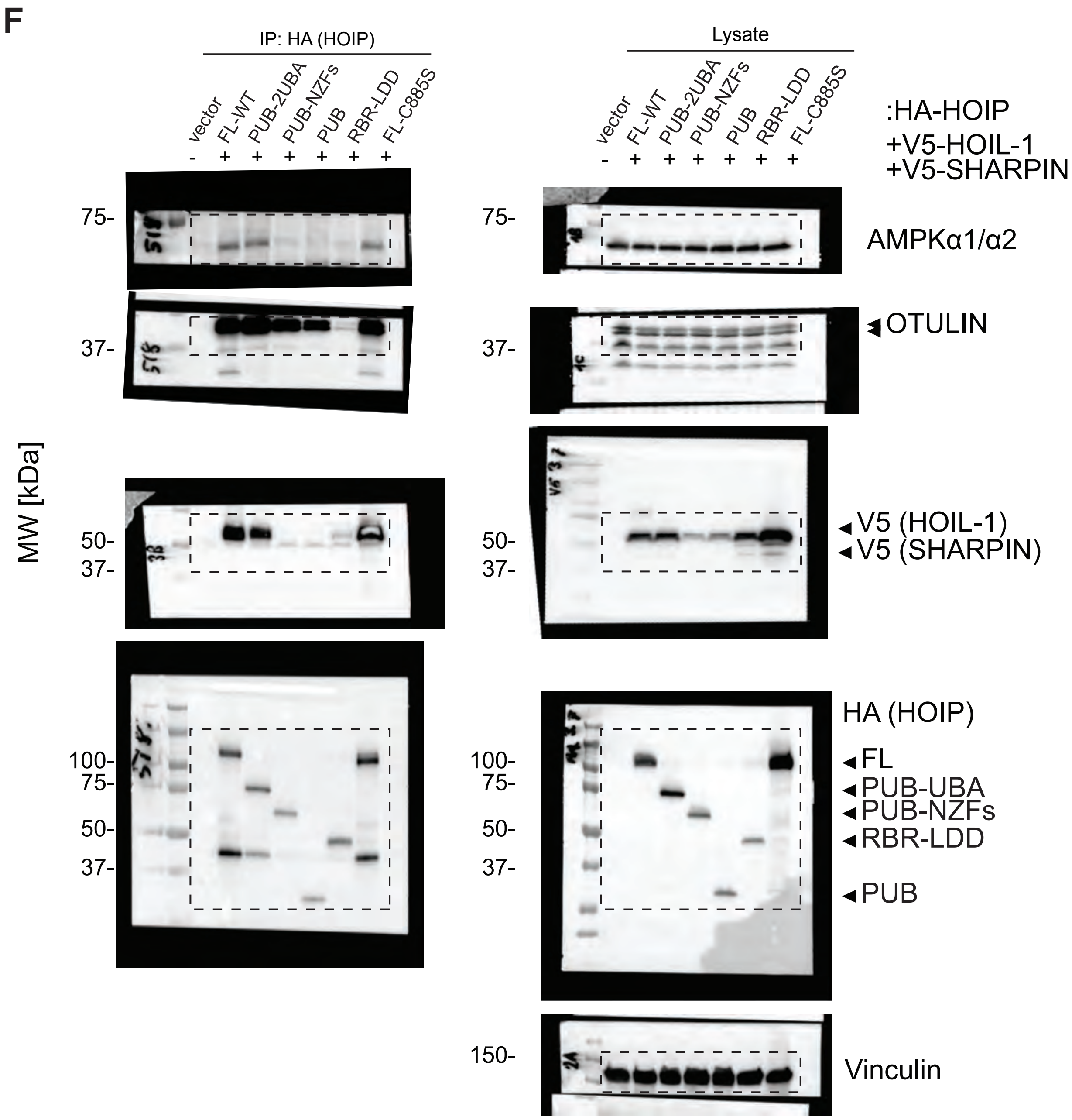
F
IP: HA (HOIP)
Lysate
vector
FL-WT
PUB-2UBA
PUB-NZFs
PUB
RBR-LDD
FL-C885S
- + + + + + +
:HA-HOIP
+V5-HOIL-1
+V5-SHARPIN
MW [kDa]
75-
37-
50-
37-
100-
75-
50-
37-
150-
AMPKα1/α2
OTULIN
V5 (HOIL-1)
V5 (SHARPIN)
HA (HOIP)
FL
PUB-UBA
PUB-NZFs
RBR-LDD
PUB
Vinculin

Figure 1

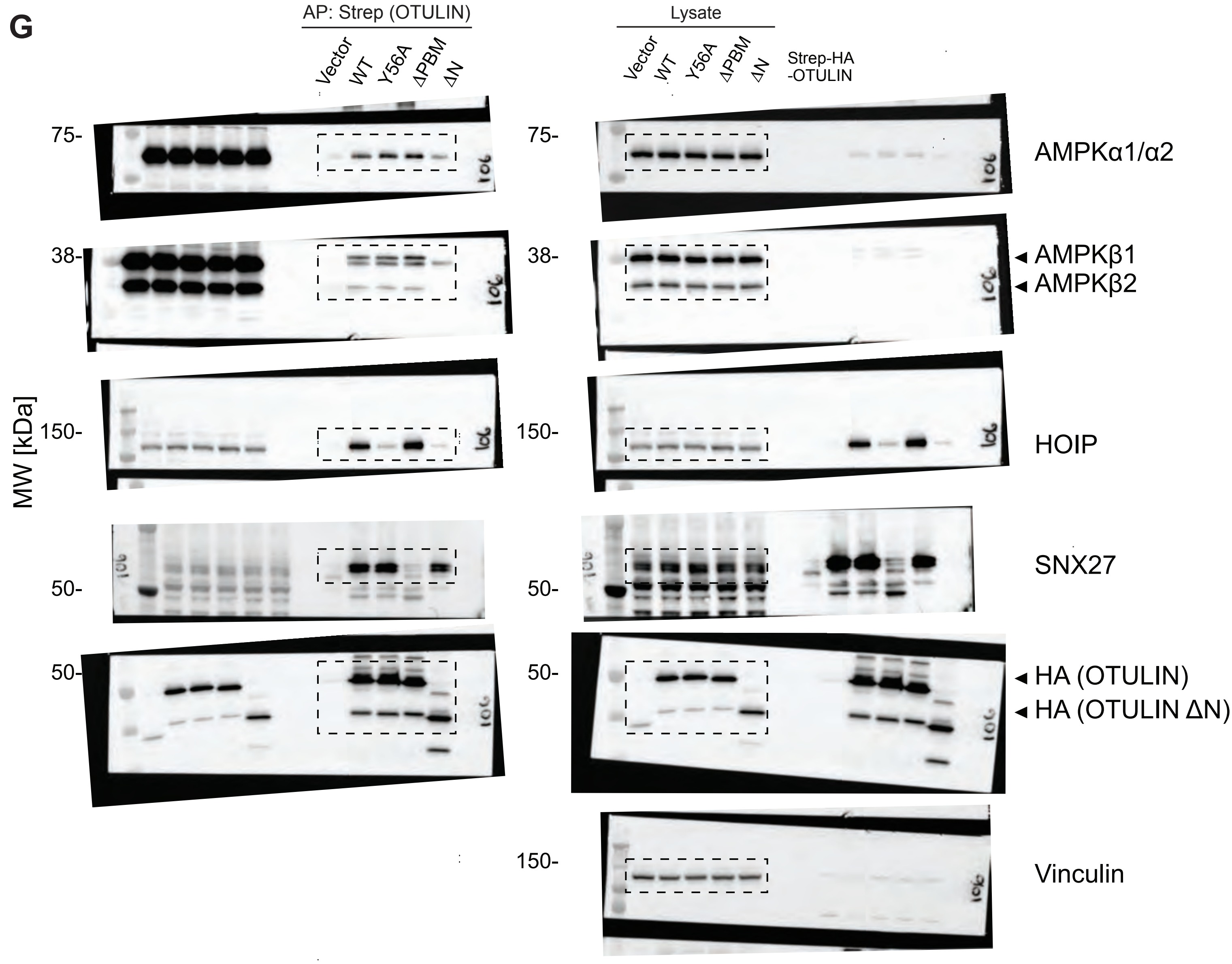
G
AP: Strep (OTULIN)
Lysate
Vector
WT
Y56A
ΔPBM
ΔN
Strep-HA
-OTULIN
MW [kDa]
75-
38-
150-
50-
AMPKα1/α2
AMPKβ1
AMPKβ2
HOIP
SNX27
HA (OTULIN)
HA (OTULIN ΔN)
Vinculin

Figure 1

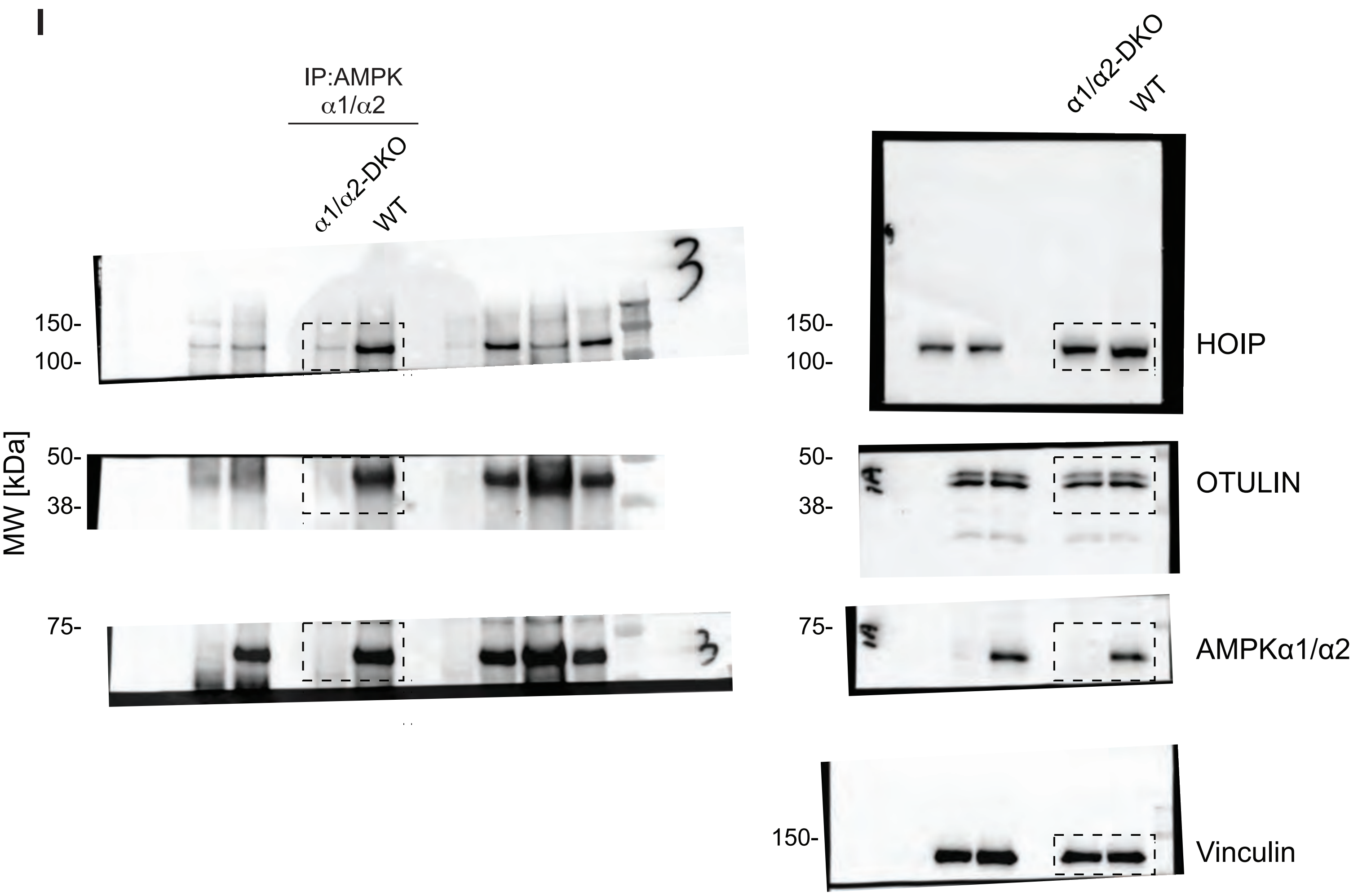
I
IP:AMPK α1/α2
α1/α2-DKO
WT
Lysate
α1/α2-DKO
WT
150-
100-
50-
38-
75-
MW [kDa]
HOIP
OTULIN
AMPKα1/α2
Vinculin

Figure 1

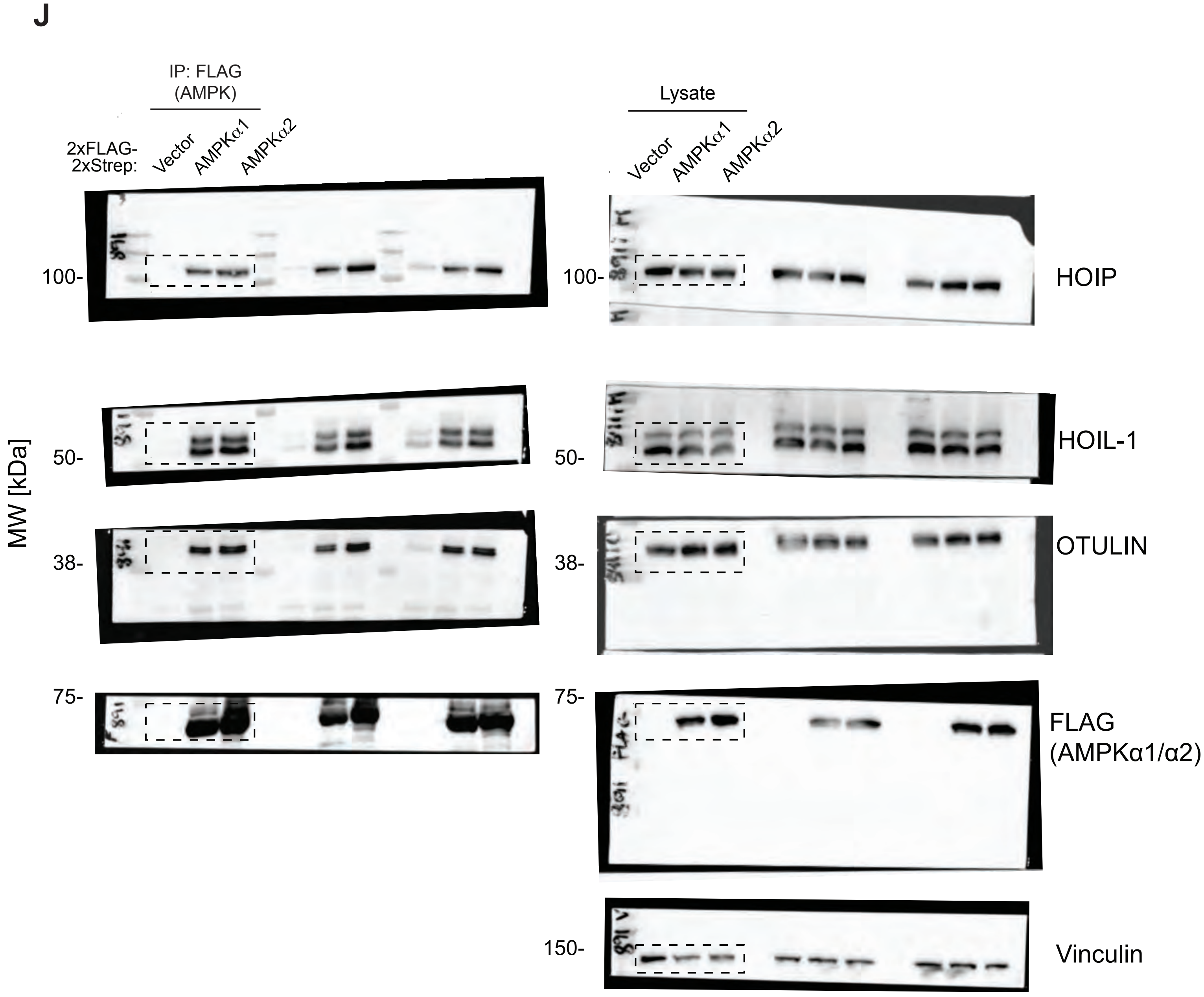
J
IP: FLAG
(AMPK)
Lysate
2xFLAG-
2xStrep:
Vector
AMPKα1
AMPKα2
100-
50-
38-
75-
150-
MW [kDa]
HOIP
HOIL-1
OTULIN
FLAG
(AMPKα1/α2)
Vinculin

Figure 2

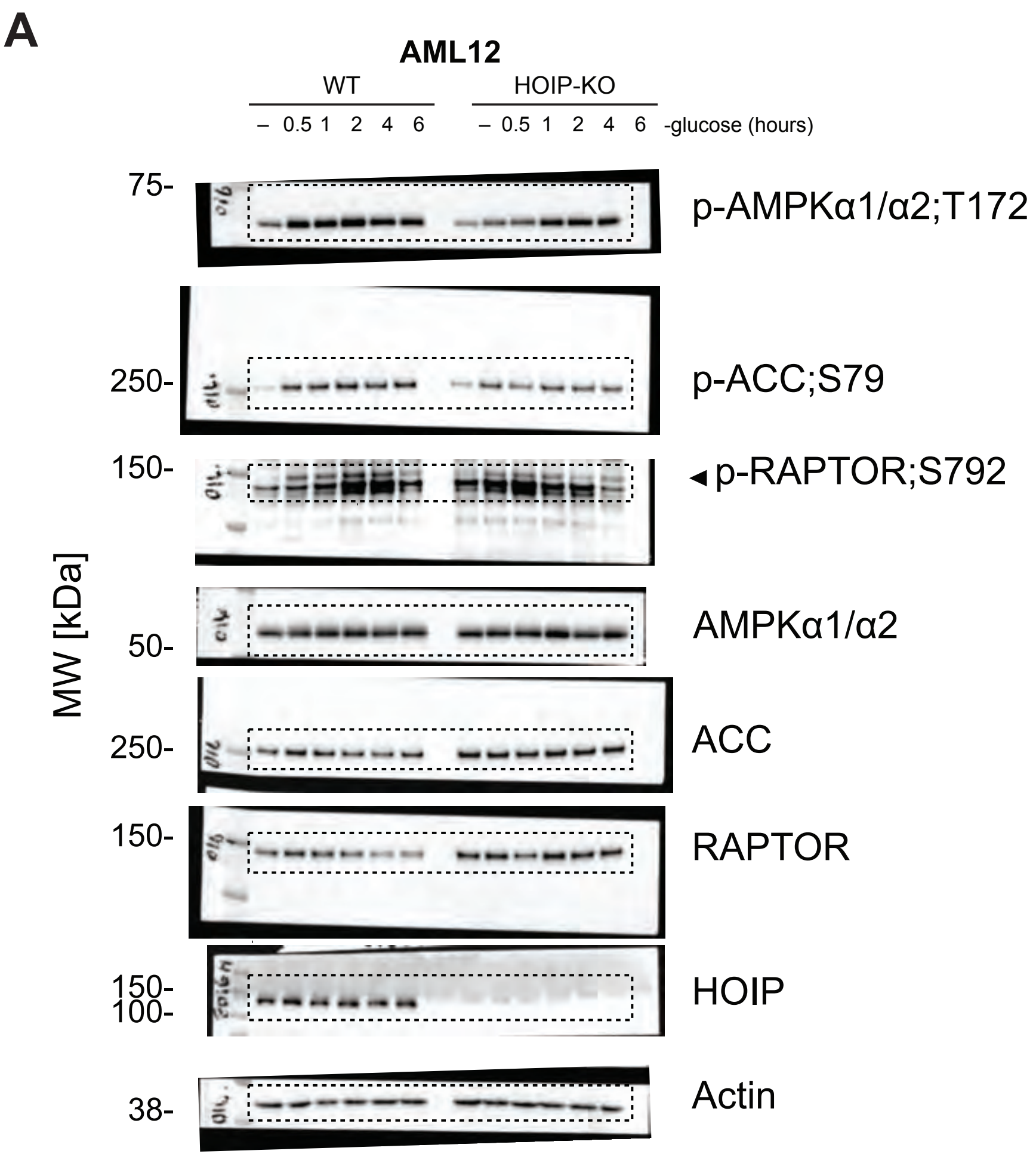
A
AML12
WT
HOIP-KO
– 0.5 1 2 4 6
– 0.5 1 2 4 6 -glucose (hours)
MW [kDa]
75-
p-AMPKα1/α2;T172
250-
p-ACC;S79
150-
p-RAPTOR;S792
50-
AMPKα1/α2
250-
ACC
150-
RAPTOR
150-
100-
HOIP
38-
Actin

Figure 2

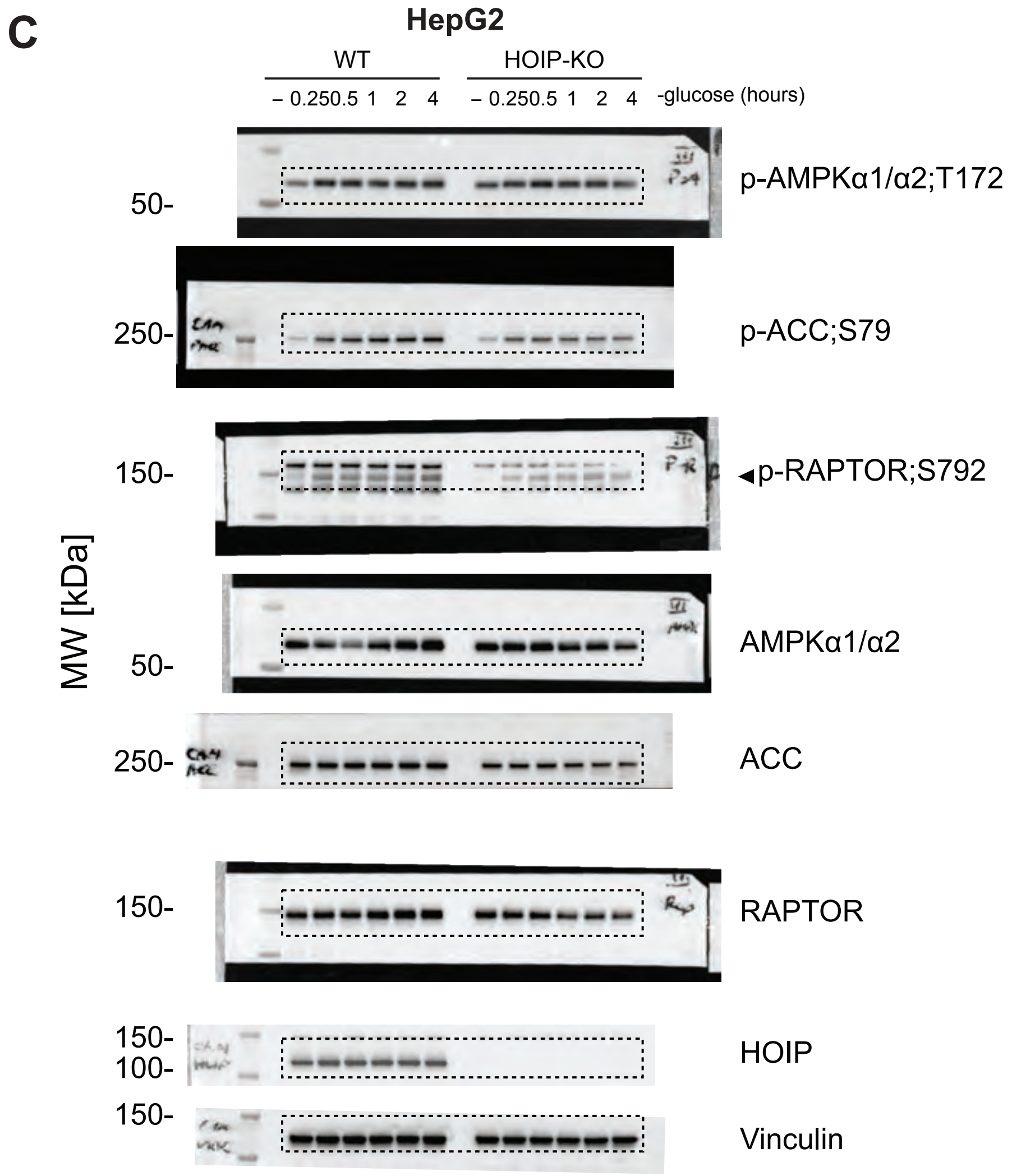
C
HepG2
WT
HOIP-KO
– 0.25 0.5 1 2 4
– 0.25 0.5 1 2 4
-glucose (hours)
MW [kDa]
50-
p-AMPKα1/α2;T172
250-
p-ACC;S79
150-
p-RAPTOR;S792
50-
AMPKα1/α2
250-
ACC
150-
RAPTOR
150-
100-
HOIP
150-
Vinculin

Figure 2

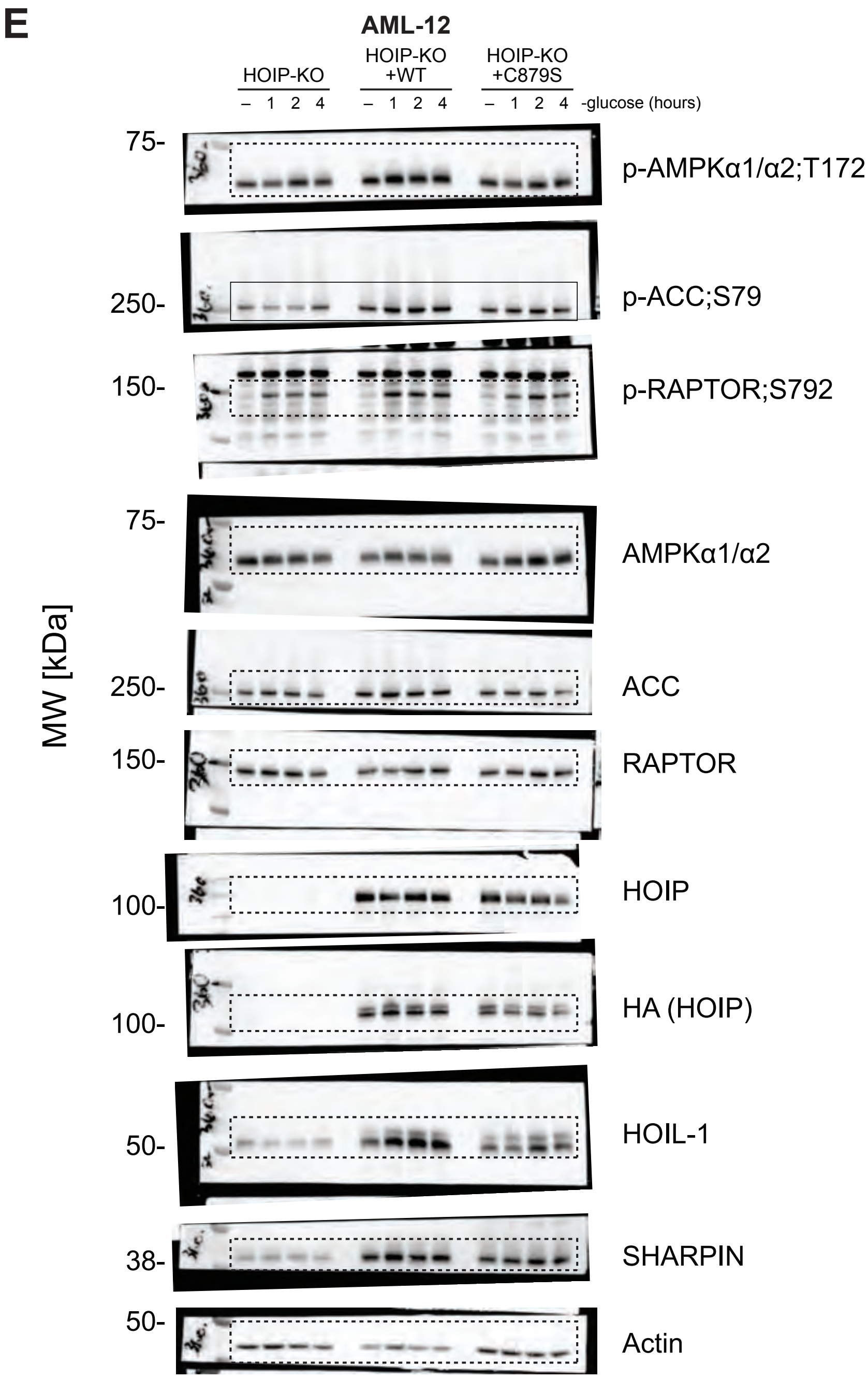
E
AML-12
HOIP-KO
HOIP-KO +WT
HOIP-KO +C879S
– 1 2 4
– 1 2 4
– 1 2 4
-glucose (hours)
MW [kDa]
75-
p-AMPKα1/α2;T172
250-
p-ACC;S79
150-
p-RAPTOR;S792
75-
AMPKα1/α2
250-
ACC
150-
RAPTOR
100-
HOIP
100-
HA (HOIP)
50-
HOIL-1
38-
SHARPIN
50-
Actin

Figure 2

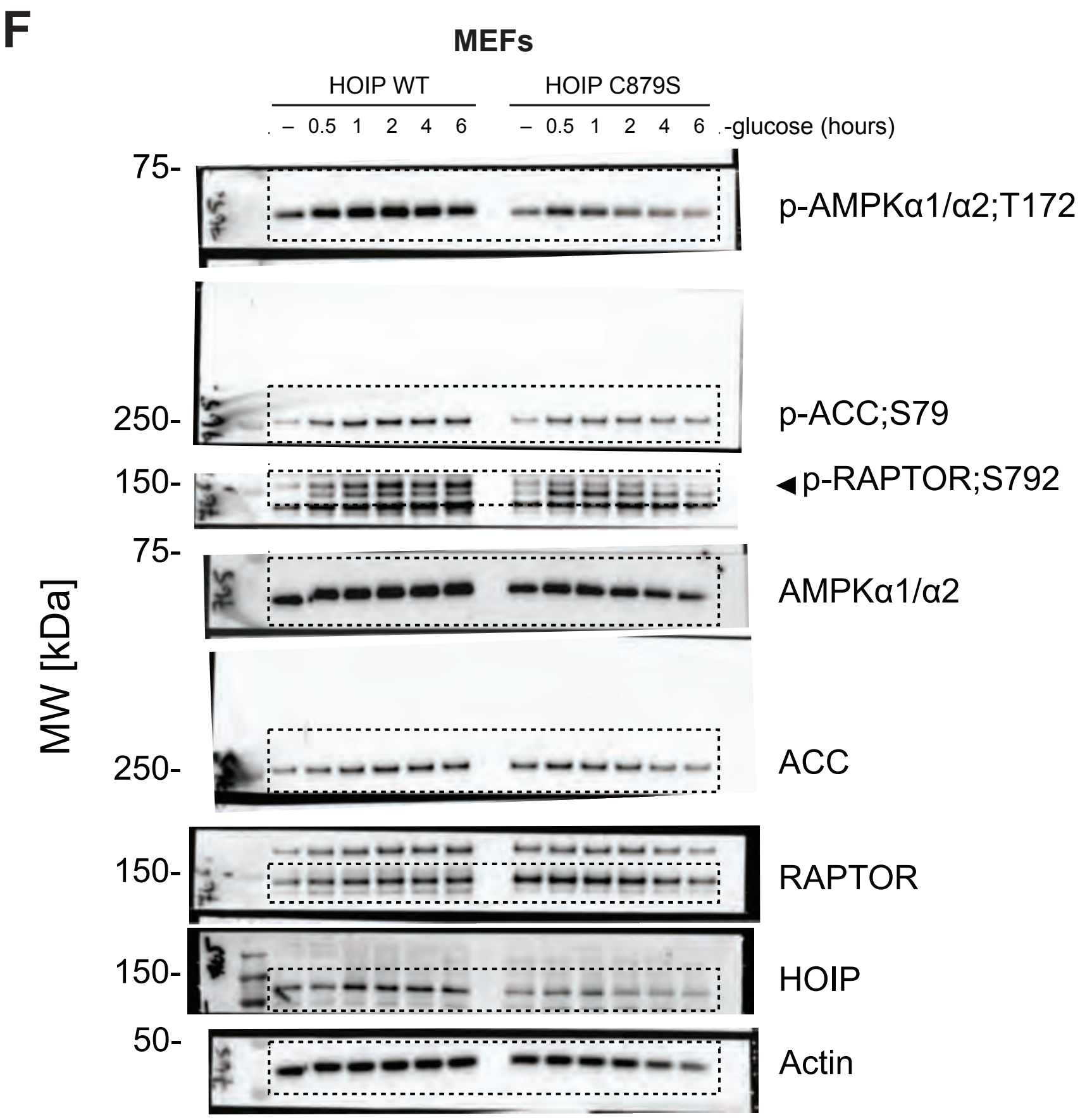

F
MEFs
HOIP WT
HOIP C879S
– 0.5 1 2 4 6
– 0.5 1 2 4 6
-glucose (hours)
75-
p-AMPKα1/α2;T172
250-
p-ACC;S79
150-
p-RAPTOR;S792
75-
AMPKα1/α2
250-
ACC
150-
RAPTOR
150-
HOIP
50-
Actin
MW [kDa]

Figure 2

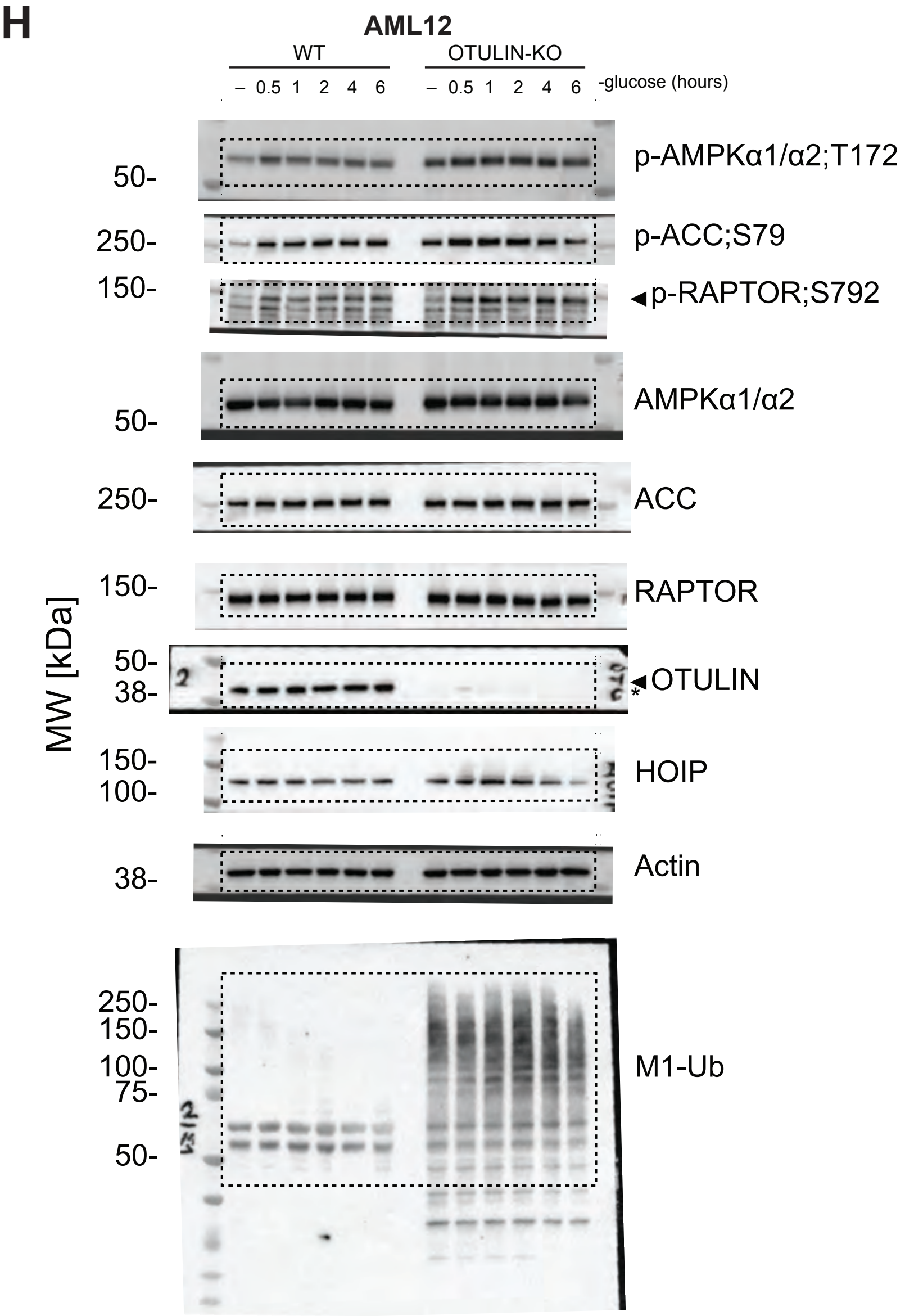
H
AML12
WT
OTULIN-KO
– 0.5 1 2 4 6
– 0.5 1 2 4 6
-glucose (hours)
50-
p-AMPKα1/α2;T172
250-
p-ACC;S79
150-
p-RAPTOR;S792
50-
AMPKα1/α2
250-
ACC
150-
RAPTOR
MW [kDa]
50-
38-
OTULIN
*
150-
100-
HOIP
38-
Actin
250-
150-
100-
75-
50-
M1-Ub

Figure 2

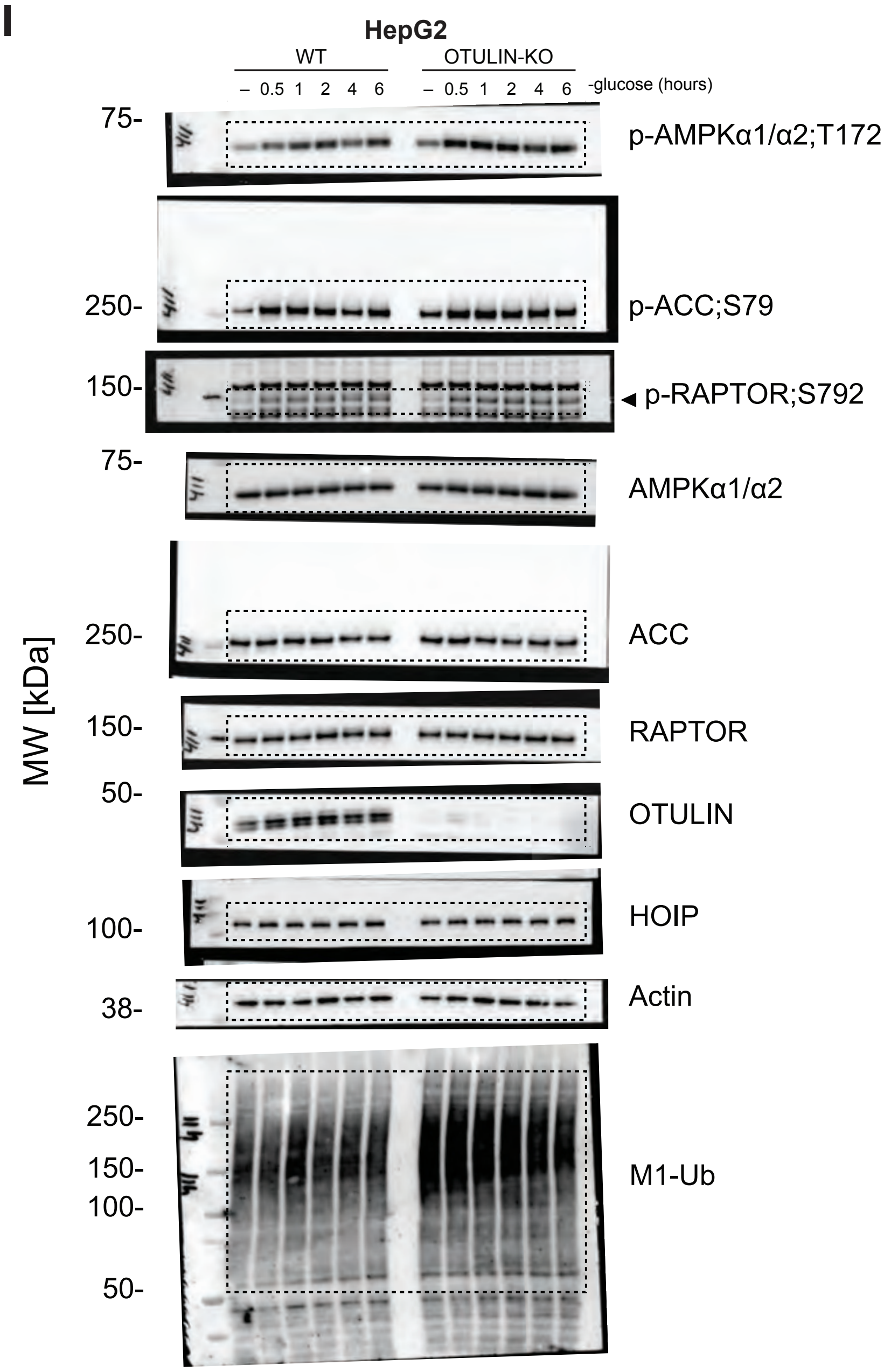
I
HepG2
WT
OTULIN-KO
– 0.5 1 2 4 6
– 0.5 1 2 4 6
-glucose (hours)
MW [kDa]
75-
p-AMPKα1/α2;T172
250-
p-ACC;S79
150-
p-RAPTOR;S792
75-
AMPKα1/α2
250-
ACC
150-
RAPTOR
50-
OTULIN
100-
HOIP
38-
Actin
250-
150-
100-
50-
M1-Ub

Figure 3

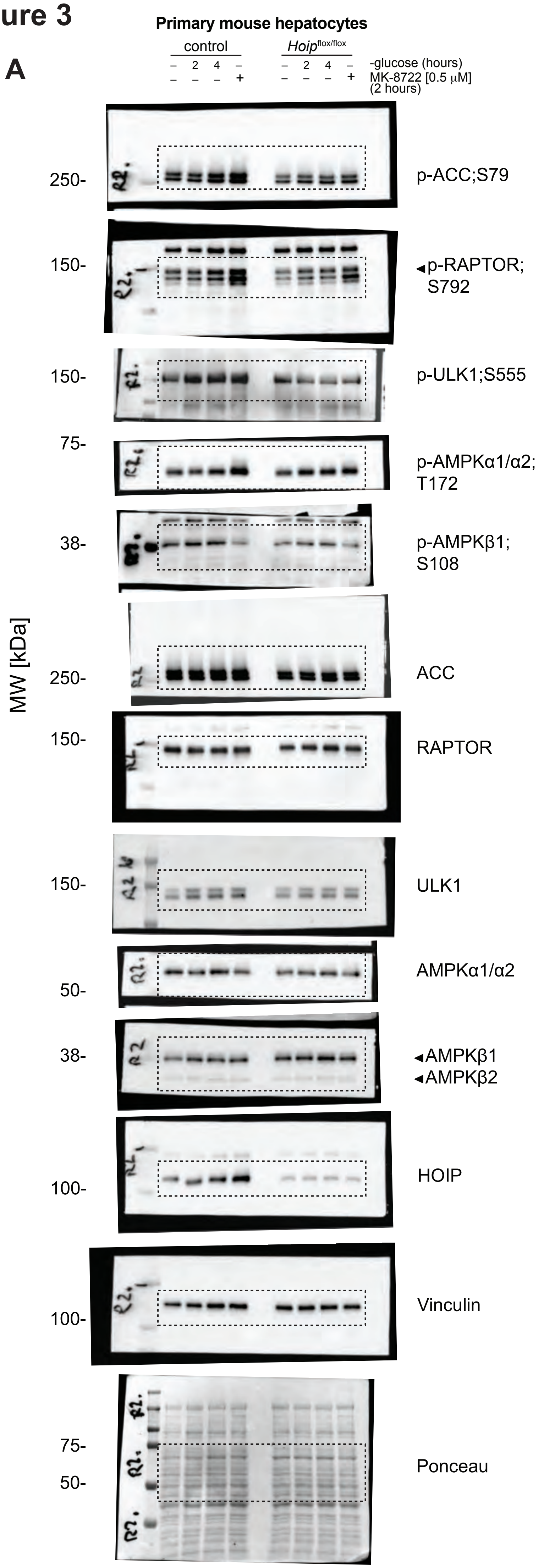
A
Primary mouse hepatocytes
control
Hoip flox/flox
– 2 4 – – 2 4 –
-glucose (hours)
– – – + – – – +
MK-8722 [0.5 µM]
(2 hours)
250-
p-ACC;S79
150-
p-RAPTOR;
S792
150-
p-ULK1;S555
75-
p-AMPKα1/α2;
T172
38-
p-AMPKβ1;
S108
MW [kDa]
250-
ACC
150-
RAPTOR
150-
ULK1
50-
AMPKα1/α2
38-
AMPKβ1
AMPKβ2
100-
HOIP
100-
Vinculin
75-
50-
Ponceau

Figure 3

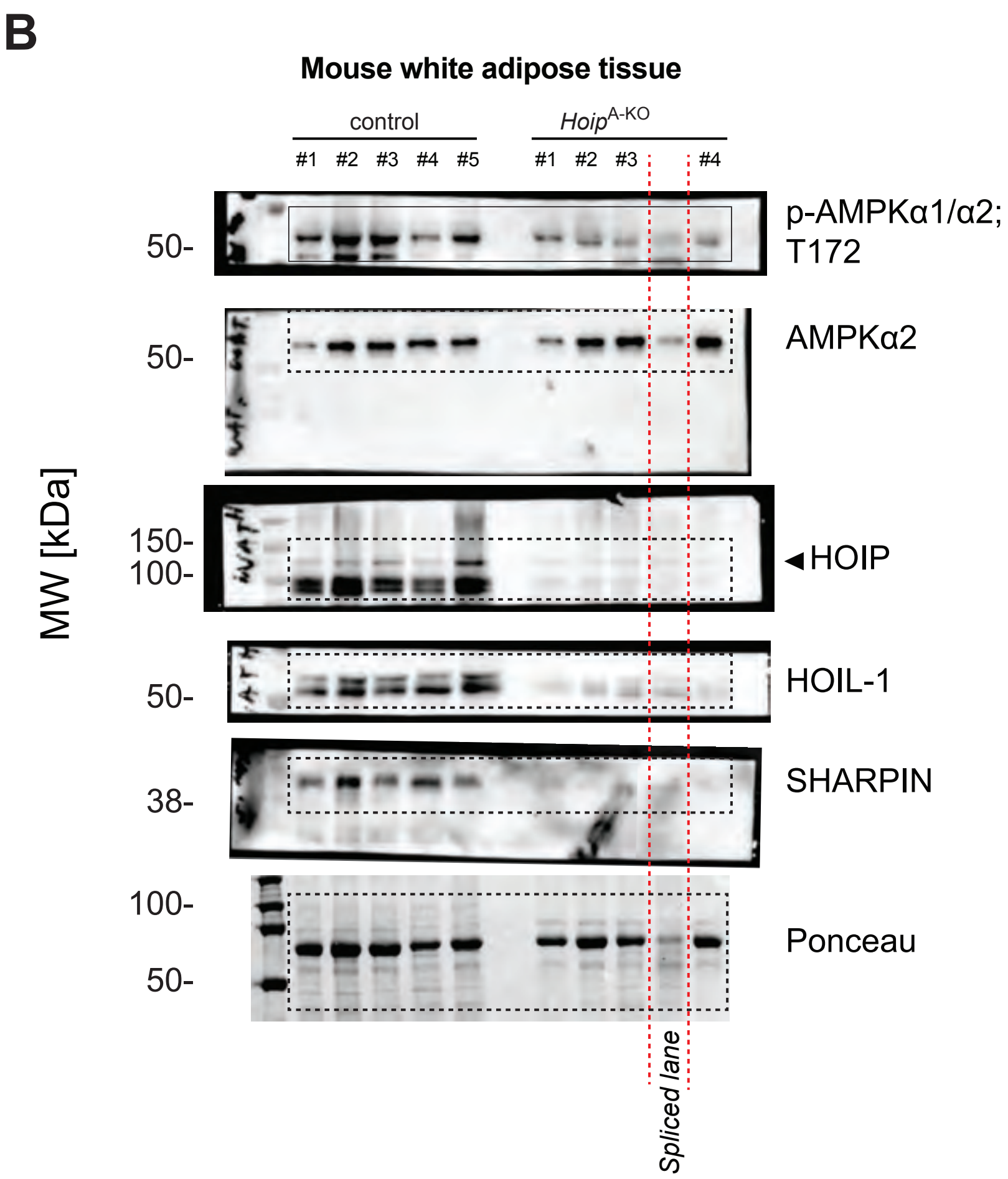
B
Mouse white adipose tissue
control
Hoip A-KO
#1 #2 #3 #4 #5
#1 #2 #3 #4
MW [kDa]
50-
p-AMPKα1/α2; T172
50-
AMPKα2
150-
100-
◄HOIP
50-
HOIL-1
38-
SHARPIN
100-
50-
Ponceau
Spliced lane

Figure 3

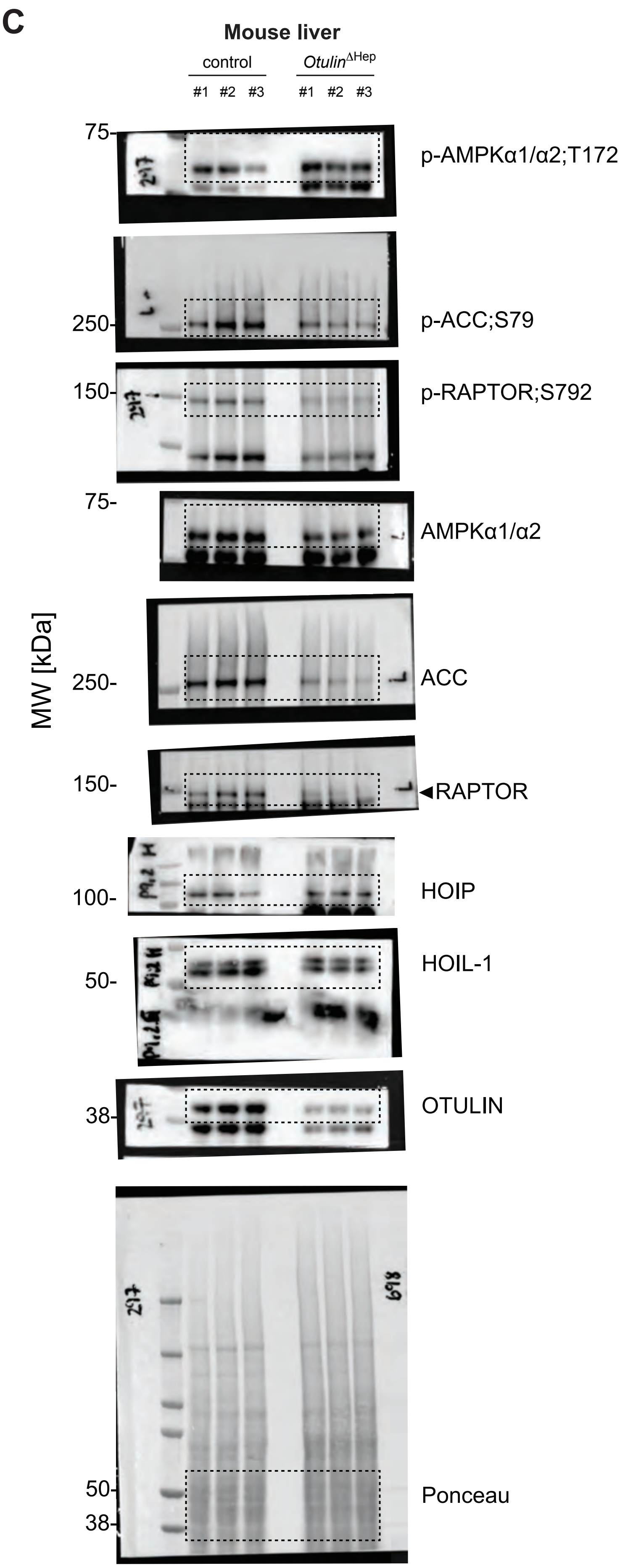
C
Mouse liver
control
Otulin^ΔHep
#1 #2 #3
#1 #2 #3
75-
p-AMPKα1/α2;T172
250-
p-ACC;S79
150-
p-RAPTOR;S792
75-
AMPKα1/α2
MW [kDa]
250-
ACC
150-
◄RAPTOR
100-
HOIP
50-
HOIL-1
38-
OTULIN
50-
38-
Ponceau

Figure 3

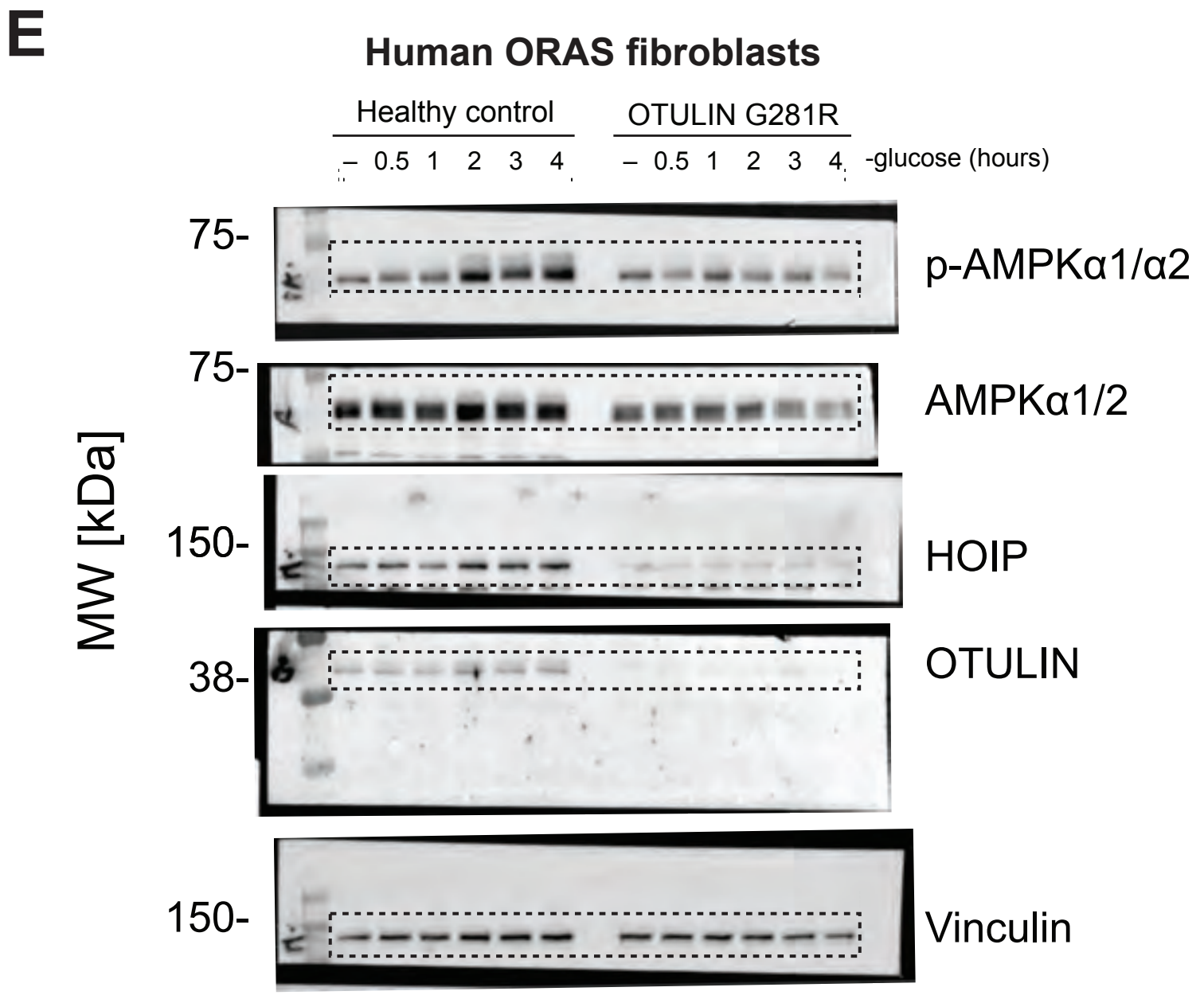
E
Human ORAS fibroblasts
Healthy control
OTULIN G281R
– 0.5 1 2 3 4
– 0.5 1 2 3 4
-glucose (hours)
MW [kDa]
75-
p-AMPKα1/α2
75-
AMPKα1/2
150-
HOIP
38-
OTULIN
150-
Vinculin

Figure 4

A

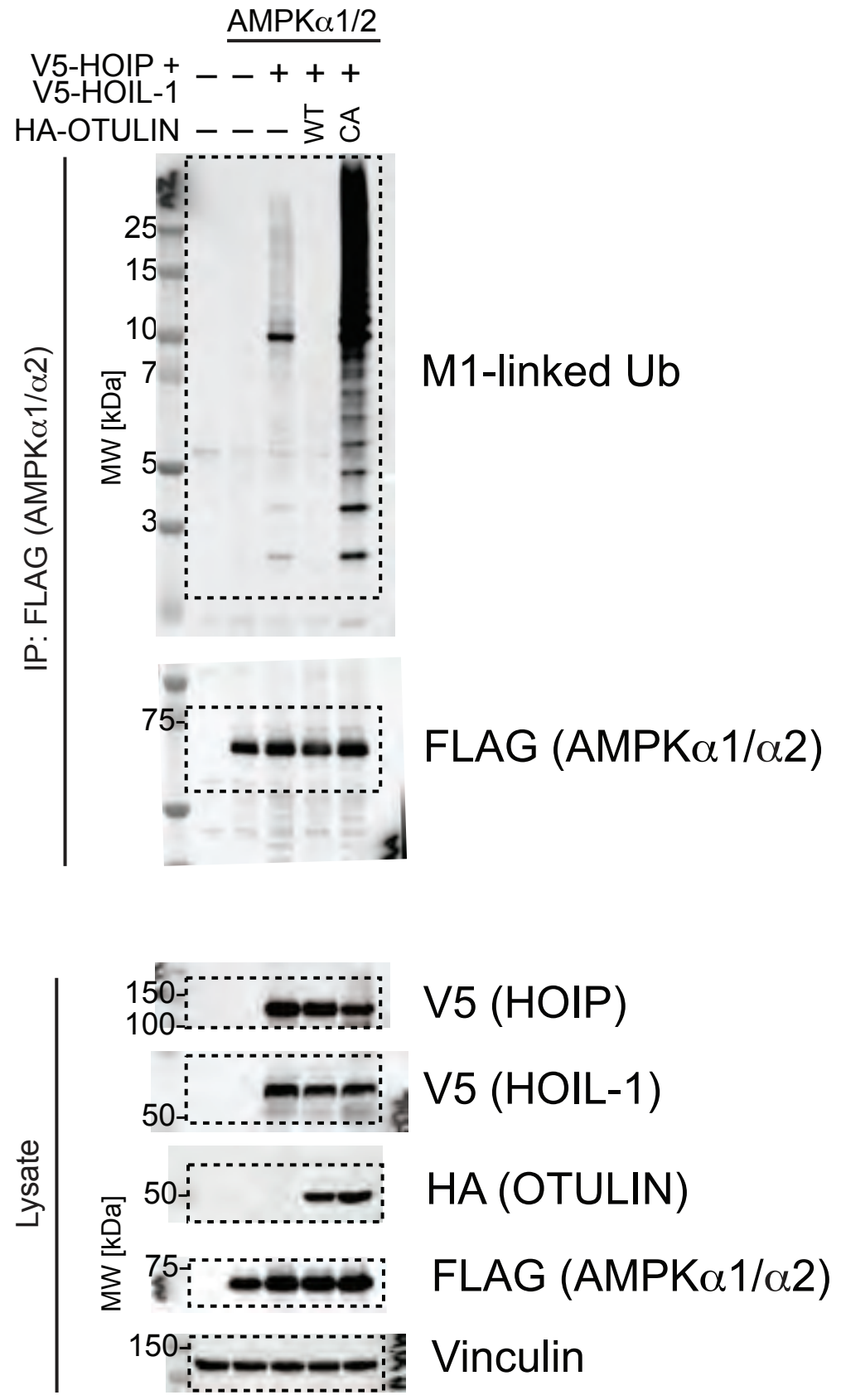
AMPKα1/2
V5-HOIP + V5-HOIL-1
HA-OTULIN
WT
CA
IP: FLAG (AMPKα1/α2)
MW [kDa]
25
15
10
7
5
3
M1-linked Ub
75
FLAG (AMPKα1/α2)
Lysate
150
100
V5 (HOIP)
50
V5 (HOIL-1)
50
HA (OTULIN)
75
FLAG (AMPKα1/α2)
150
Vinculin

Figure 4

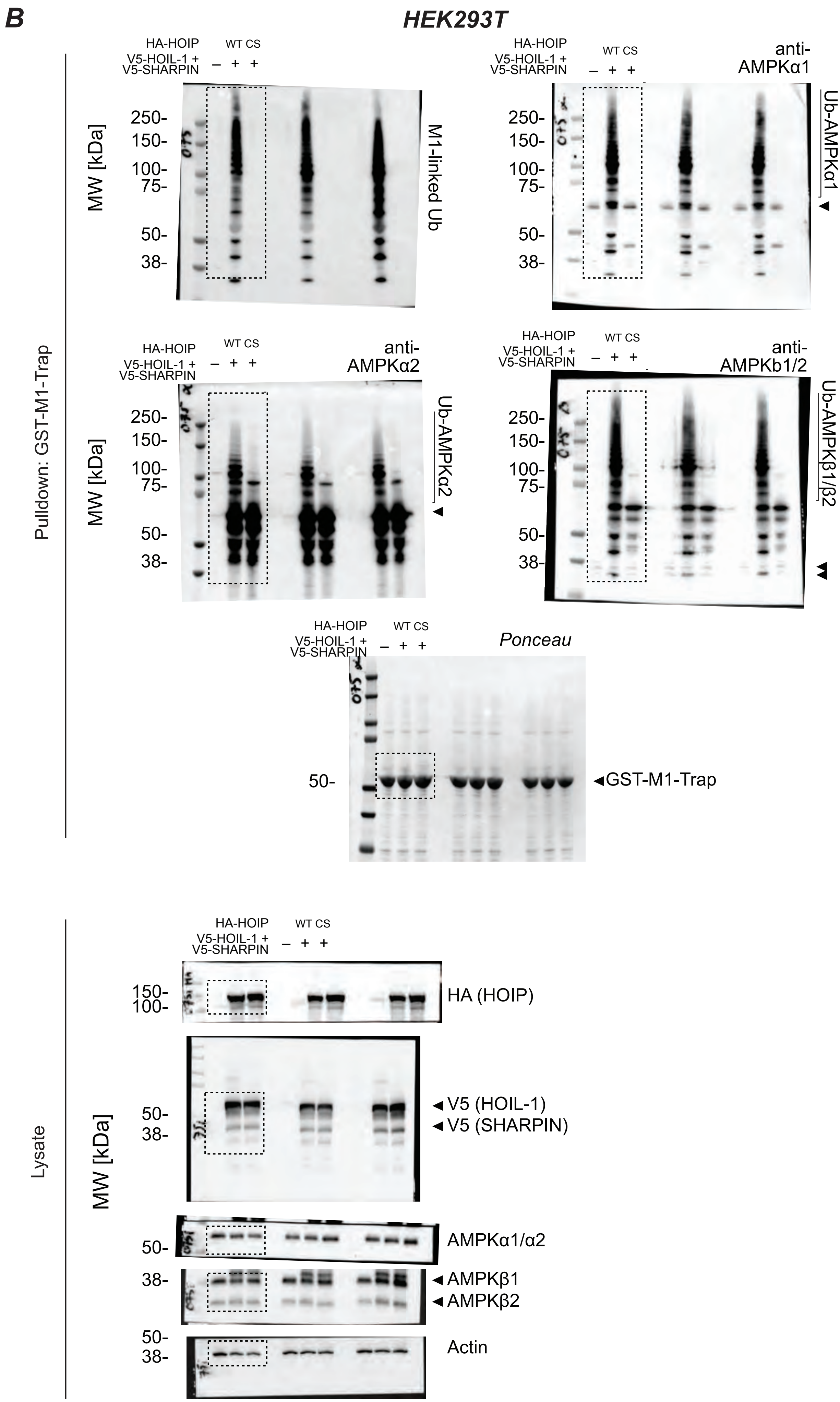
B
HEK293T
HA-HOIP
WT CS
V5-HOIL-1 +
V5-SHARPIN
MW [kDa]
250-
150-
100-
75-
50-
38-
M1-linked Ub
anti-
AMPKα1
Ub-AMPKα1
anti-
AMPKα2
Ub-AMPKα2
anti-
AMPKb1/2
Ub-AMPKβ1/β2
Pulldown: GST-M1-Trap
Ponceau
GST-M1-Trap
Lysate
HA (HOIP)
V5 (HOIL-1)
V5 (SHARPIN)
AMPKα1/α2
AMPKβ1
AMPKβ2
Actin

Figure 4

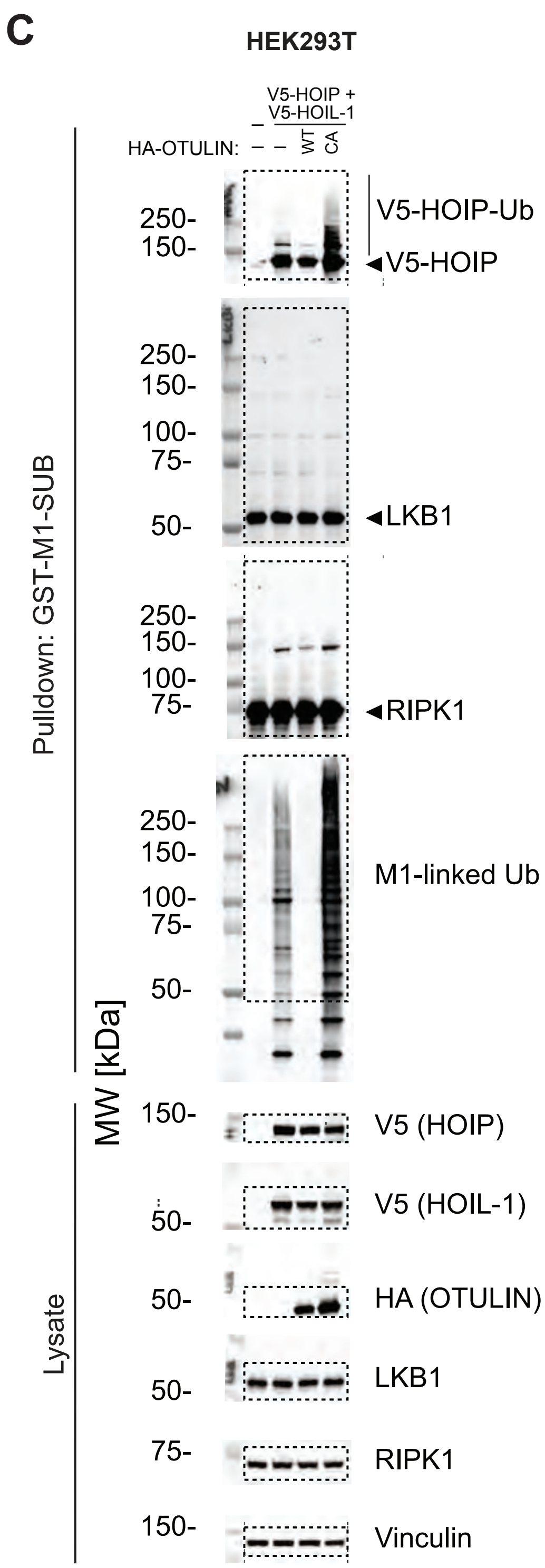
C
HEK293T
V5-HOIP +
V5-HOIL-1
HA-OTULIN:
WT
CA
Pulldown: GST-M1-SUB
250-
150-
V5-HOIP-Ub
V5-HOIP
100-
75-
50-
LKB1
RIPK1
M1-linked Ub
MW [kDa]
Lysate
V5 (HOIP)
V5 (HOIL-1)
HA (OTULIN)
LKB1
RIPK1
Vinculin

Figure 4

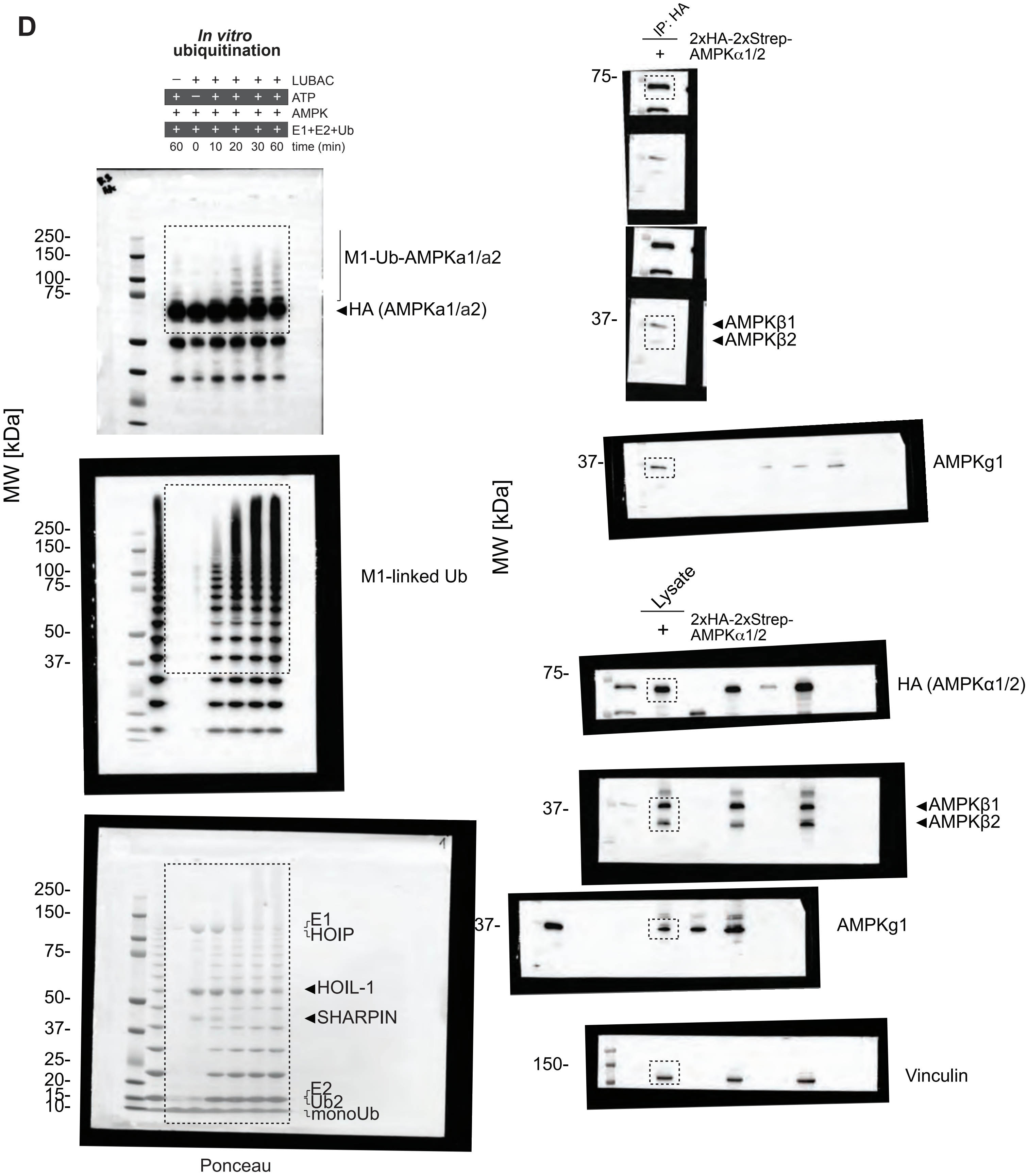

D
In vitro ubiquitination
− + + + + + LUBAC
+ − + + + + ATP
+ + + + + + AMPK
+ + + + + + E1+E2+Ub
60 0 10 20 30 60 time (min)
MW [kDa]
250-
150-
100-
75-
M1-Ub-AMPKa1/a2
◂HA (AMPKa1/a2)
250-
150-
100-
75-
50-
37-
M1-linked Ub
250-
150-
75-
50-
37-
25-
20-
15-
10-
E1
HOIP
◂HOIL-1
◂SHARPIN
E2
Ub2
monoUb
Ponceau
IP: HA
+
2xHA-2xStrep-AMPKα1/2
75-
37-
◂AMPKβ1
◂AMPKβ2
37-
AMPKg1
MW [kDa]
Lysate
+
2xHA-2xStrep-AMPKα1/2
75-
HA (AMPKα1/2)
37-
◂AMPKβ1
◂AMPKβ2
37-
AMPKg1
150-
Vinculin

Figure 5

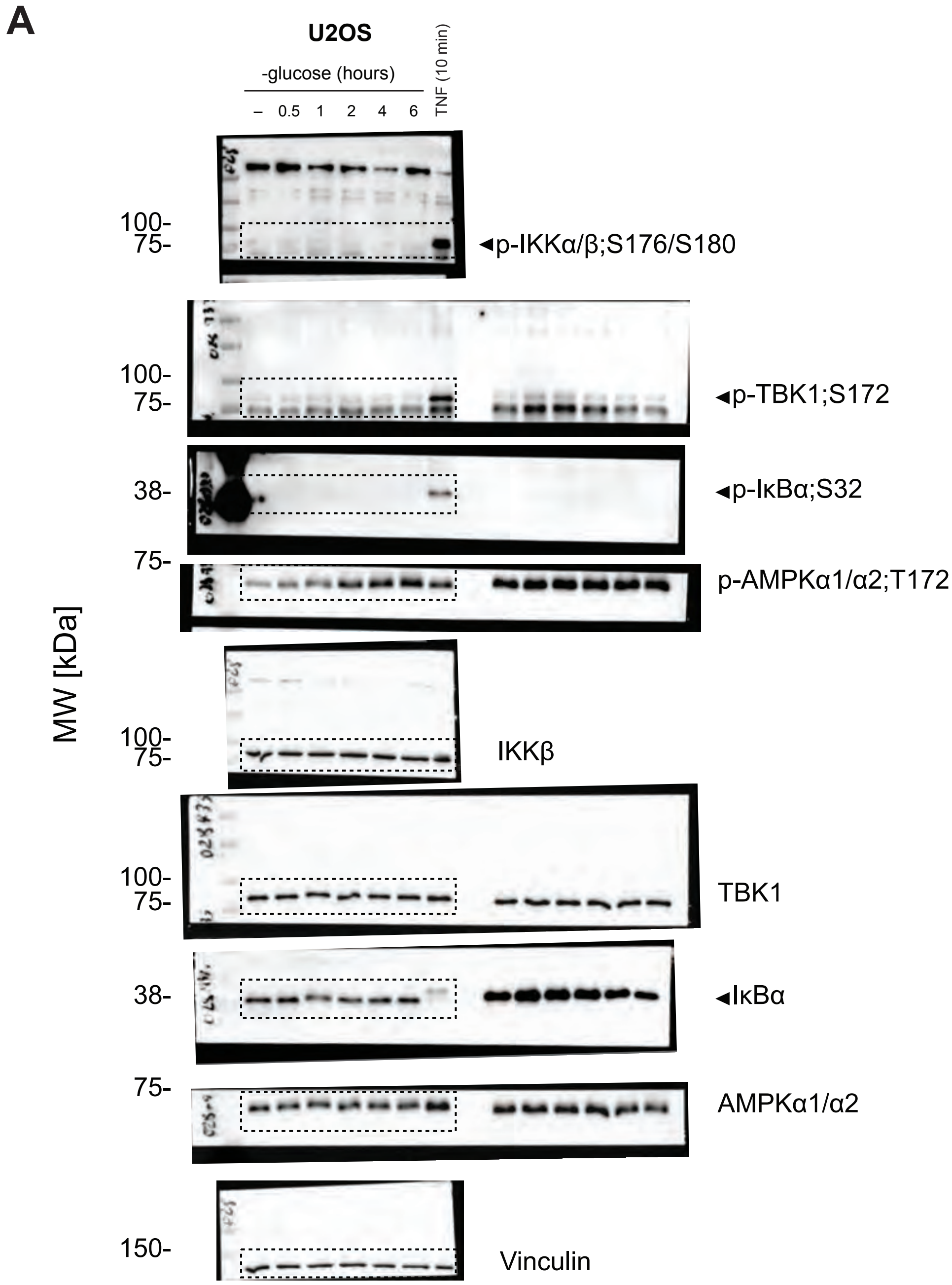
A
U2OS
-glucose (hours)
– 0.5 1 2 4 6
TNF (10 min)
100-
75-
◂p-IKKα/β;S176/S180
100-
75-
◂p-TBK1;S172
38-
◂p-IκBα;S32
75-
p-AMPKα1/α2;T172
MW [kDa]
100-
75-
IKKβ
100-
75-
TBK1
38-
◂IκBα
75-
AMPKα1/α2
150-
Vinculin

Figure 5

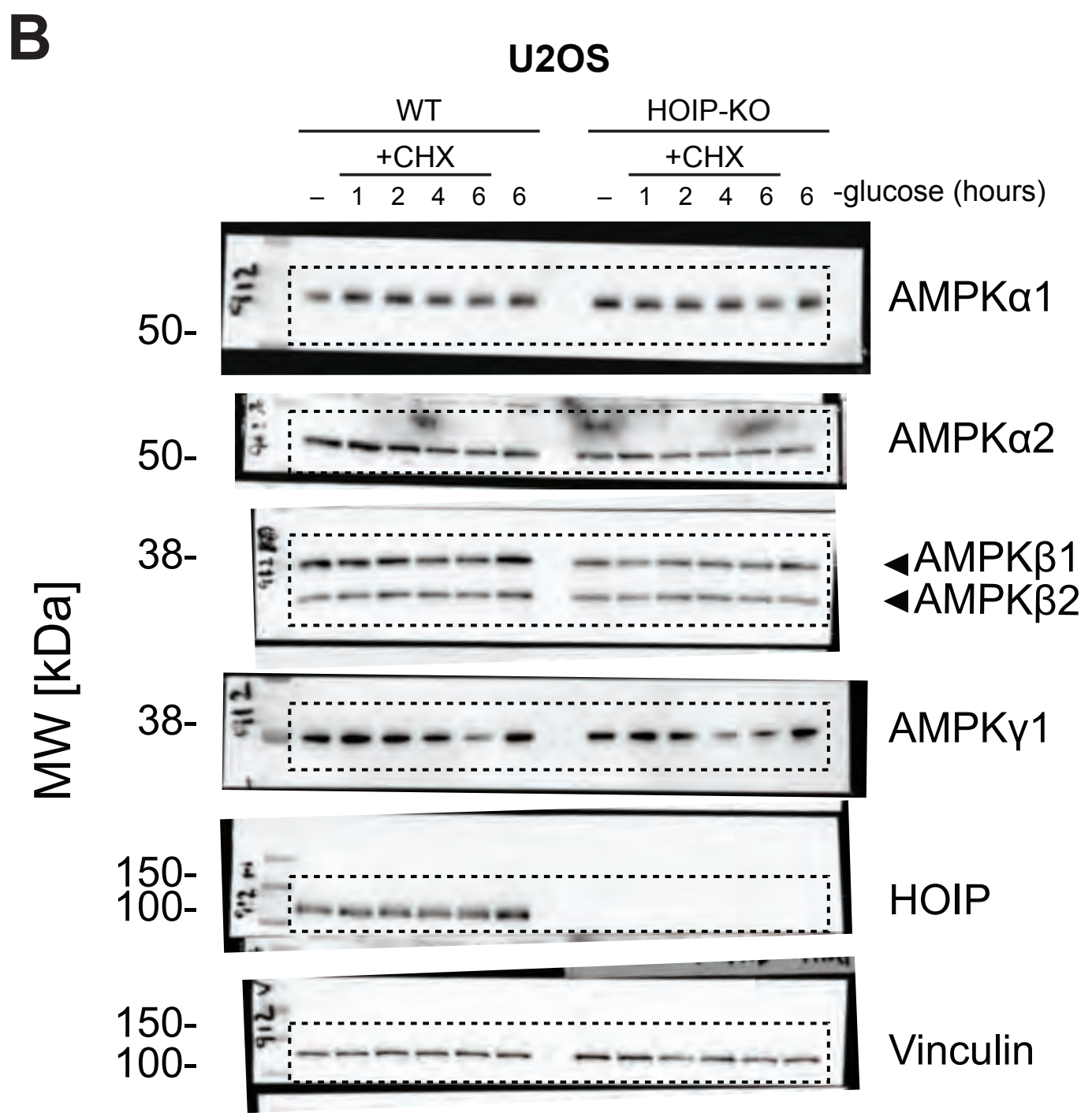
B
U2OS
WT
HOIP-KO
+CHX
+CHX
– 1 2 4 6 6
– 1 2 4 6 6 -glucose (hours)
AMPKα1
AMPKα2
AMPKβ1
AMPKβ2
AMPKγ1
HOIP
Vinculin
MW [kDa]
50-
50-
38-
38-
150-
100-
150-
100-

Figure 5

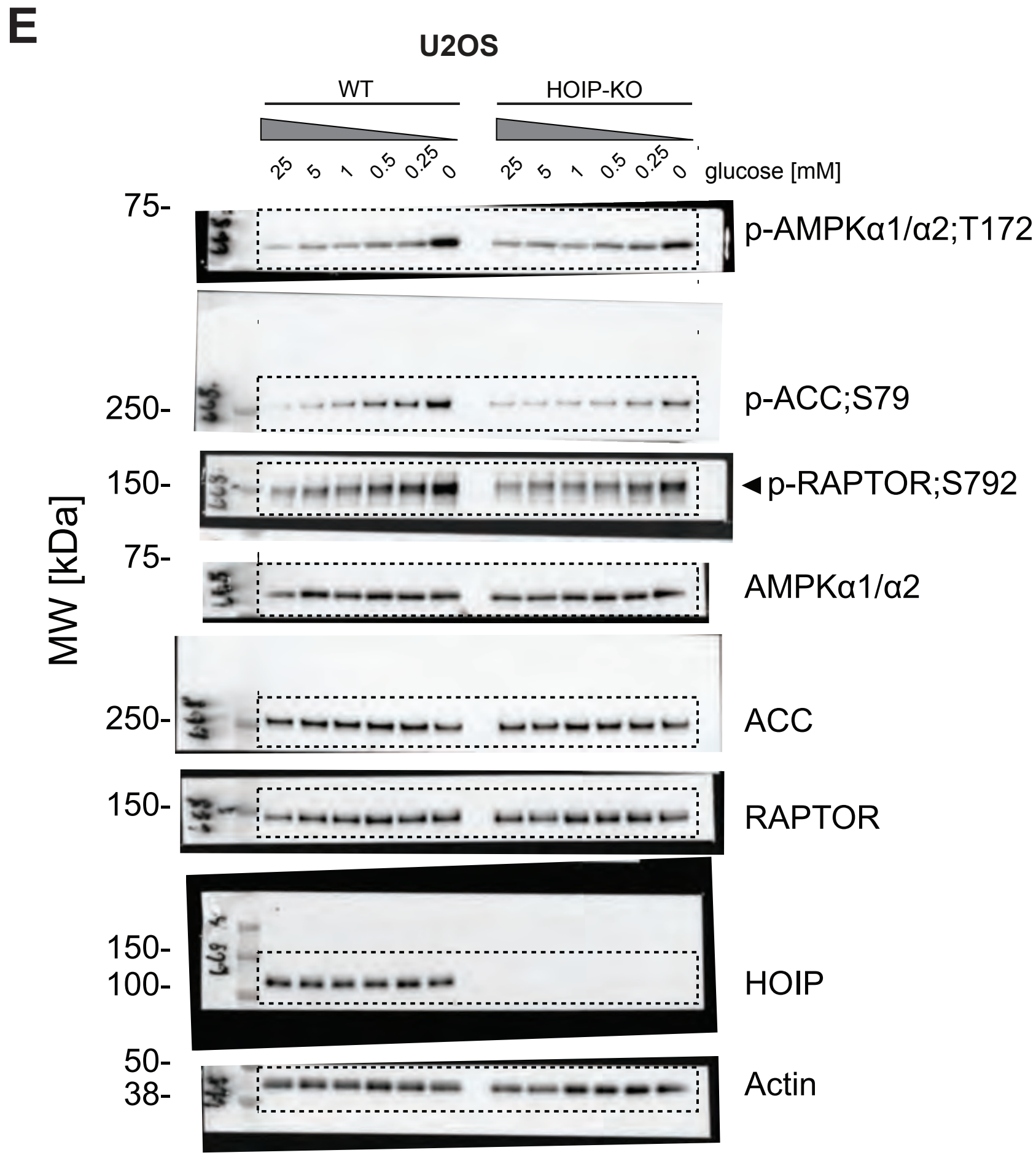
E
U2OS
WT
HOIP-KO
25 5 1 0.5 0.25 0
25 5 1 0.5 0.25 0
glucose [mM]
MW [kDa]
75-
250-
150-
75-
250-
150-
150-
100-
50-
38-
p-AMPKα1/α2;T172
p-ACC;S79
◄p-RAPTOR;S792
AMPKα1/α2
ACC
RAPTOR
HOIP
Actin

Figure 5

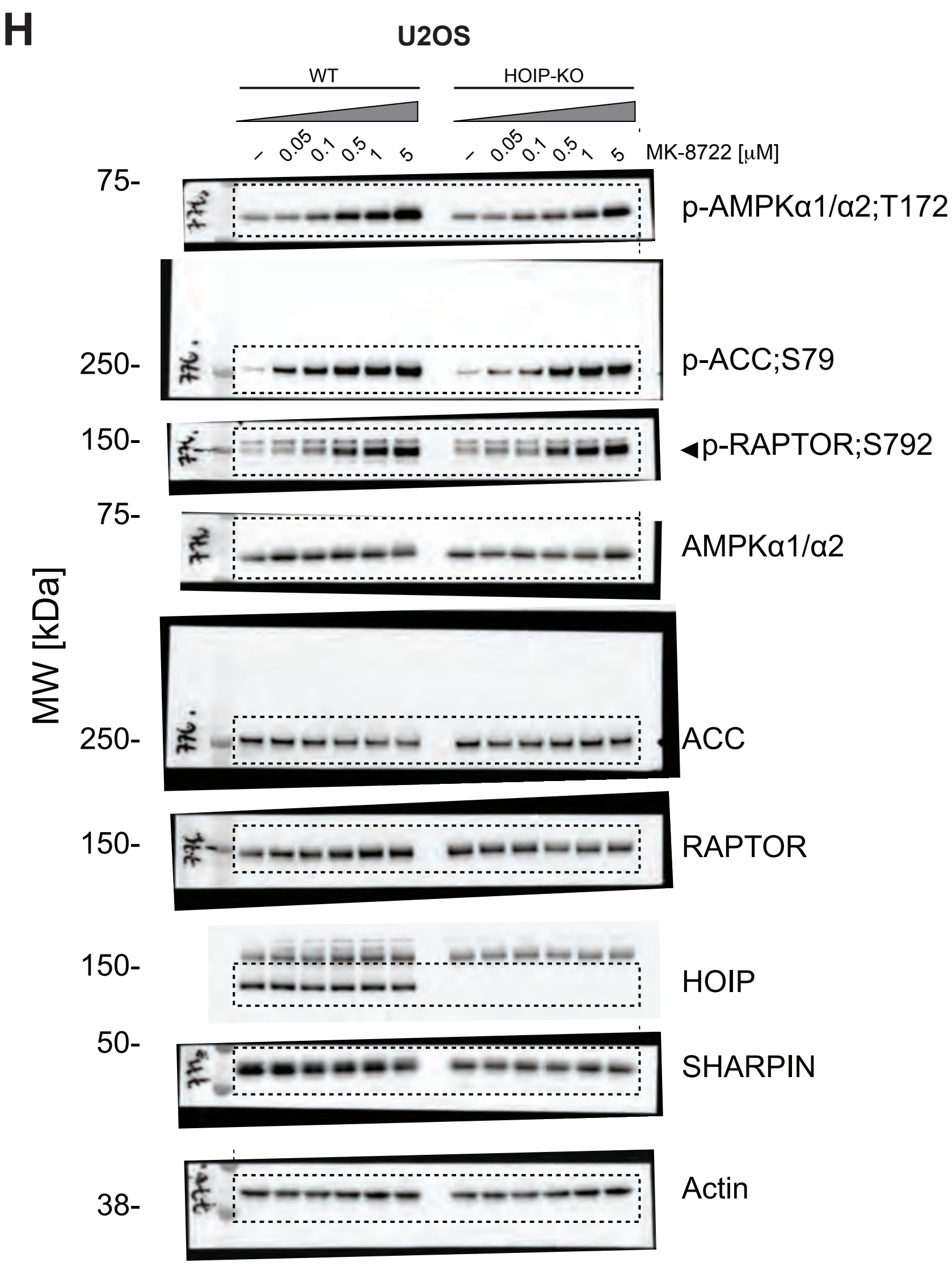
H
U2OS
WT
HOIP-KO
- 0.05 0.1 0.5 1 5
- 0.05 0.1 0.5 1 5
MK-8722 [µM]
MW [kDa]
75-
p-AMPKα1/α2;T172
250-
p-ACC;S79
150-
p-RAPTOR;S792
75-
AMPKα1/α2
250-
ACC
150-
RAPTOR
150-
HOIP
50-
SHARPIN
38-
Actin

Figure 6

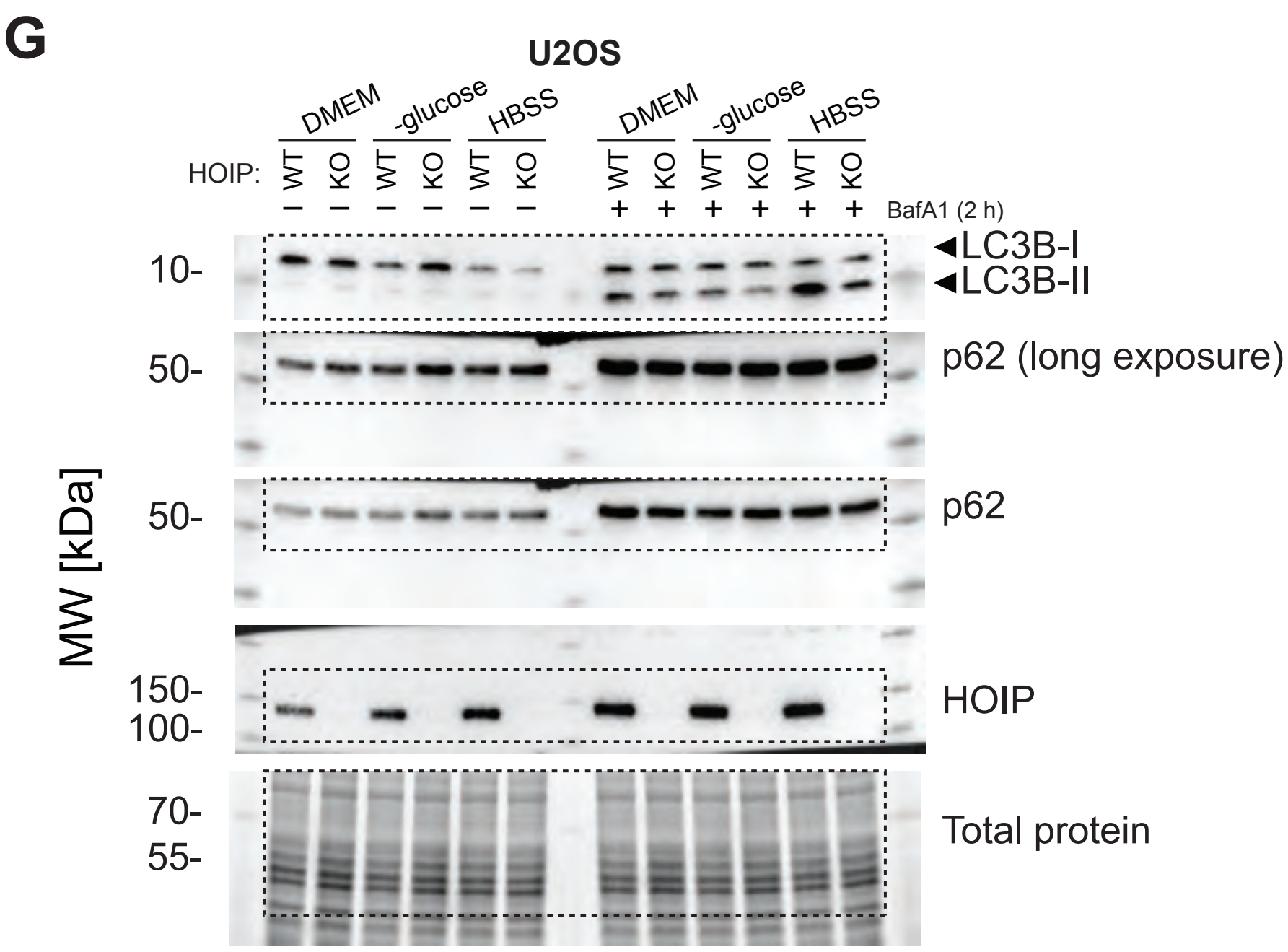

G
U2OS
DMEM
-glucose
HBSS
DMEM
-glucose
HBSS
HOIP:
WT
KO
WT
KO
WT
KO
WT
KO
WT
KO
WT
KO
BafA1 (2 h)
10-
50-
50-
150-
100-
70-
55-
MW [kDa]
◀LC3B-I
◀LC3B-II
p62 (long exposure)
p62
HOIP
Total protein

Figure 7

G

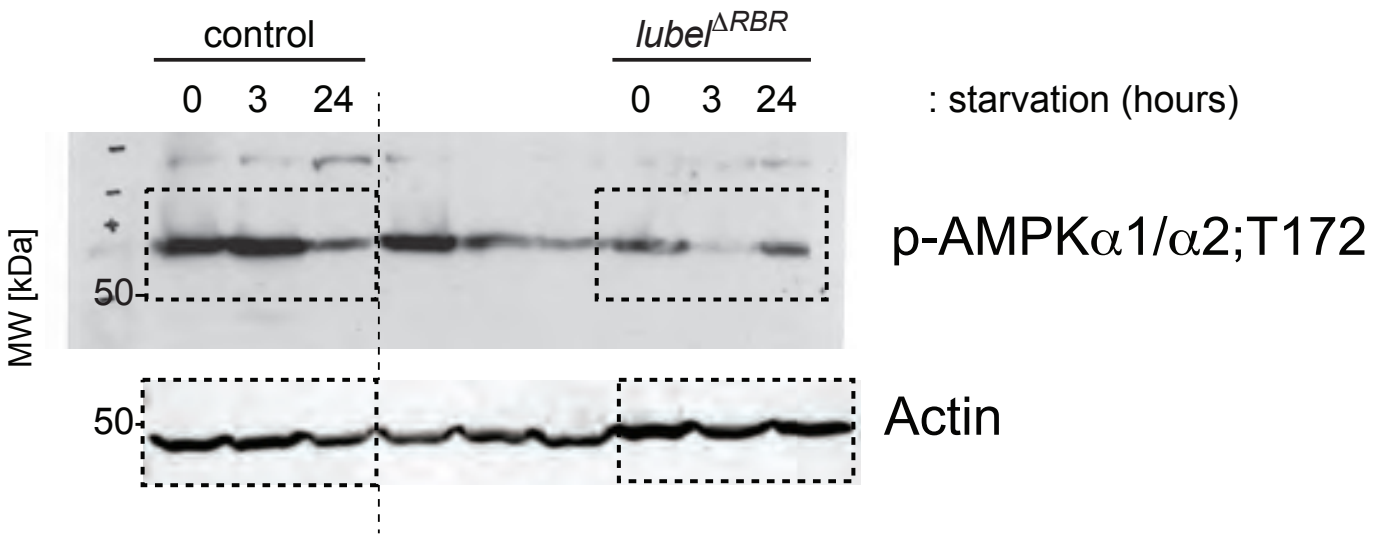
control
lubel^ΔRBR
0 3 24 0 3 24 : starvation (hours)
MW [kDa]
50
p-AMPKα1/α2;T172
50
Actin

Figure 7

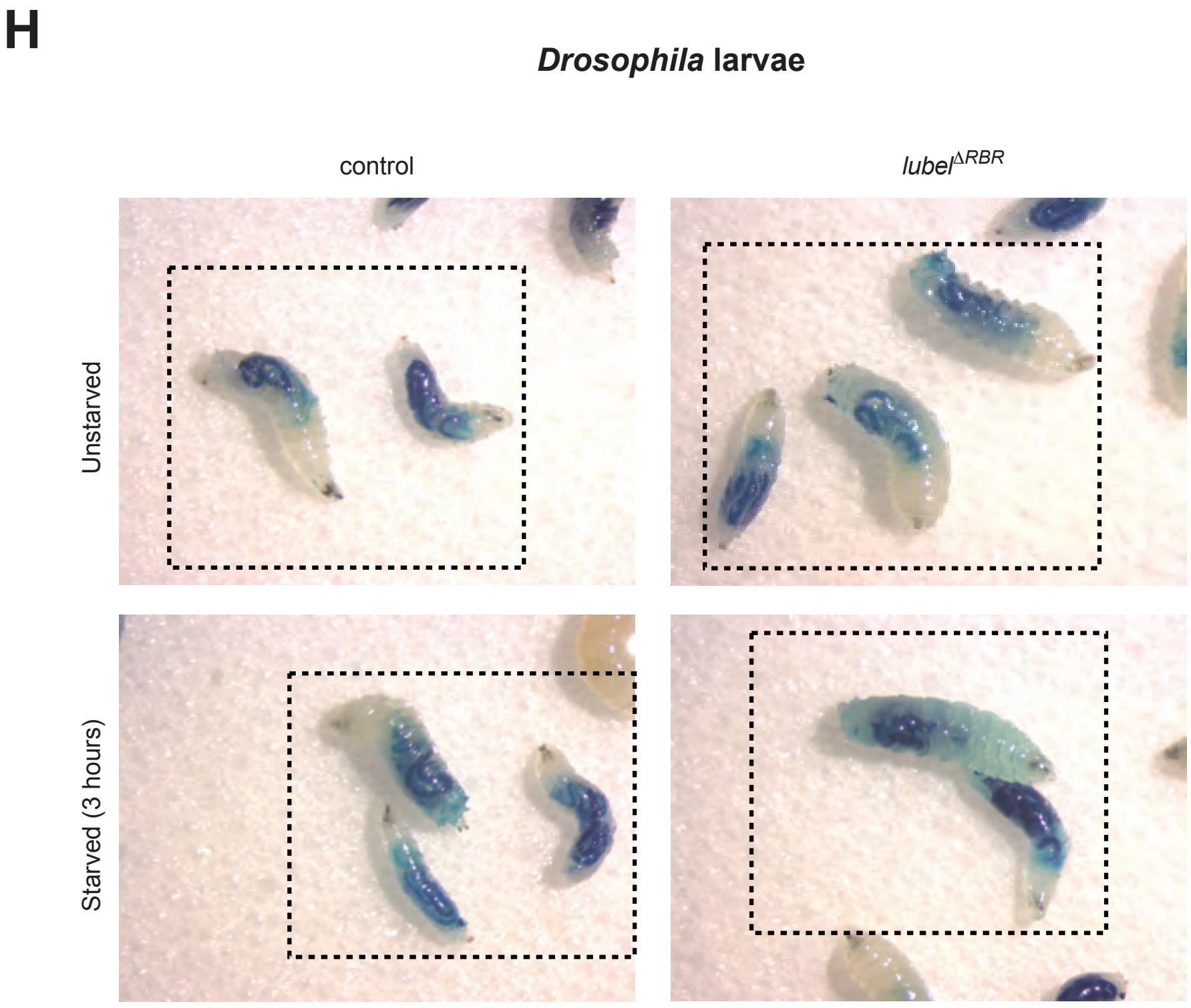
H
Drosophila larvae
control
lubel$^{\Delta RBR}$
Unstarved
Starved (3 hours)

Supplementary Figure 2

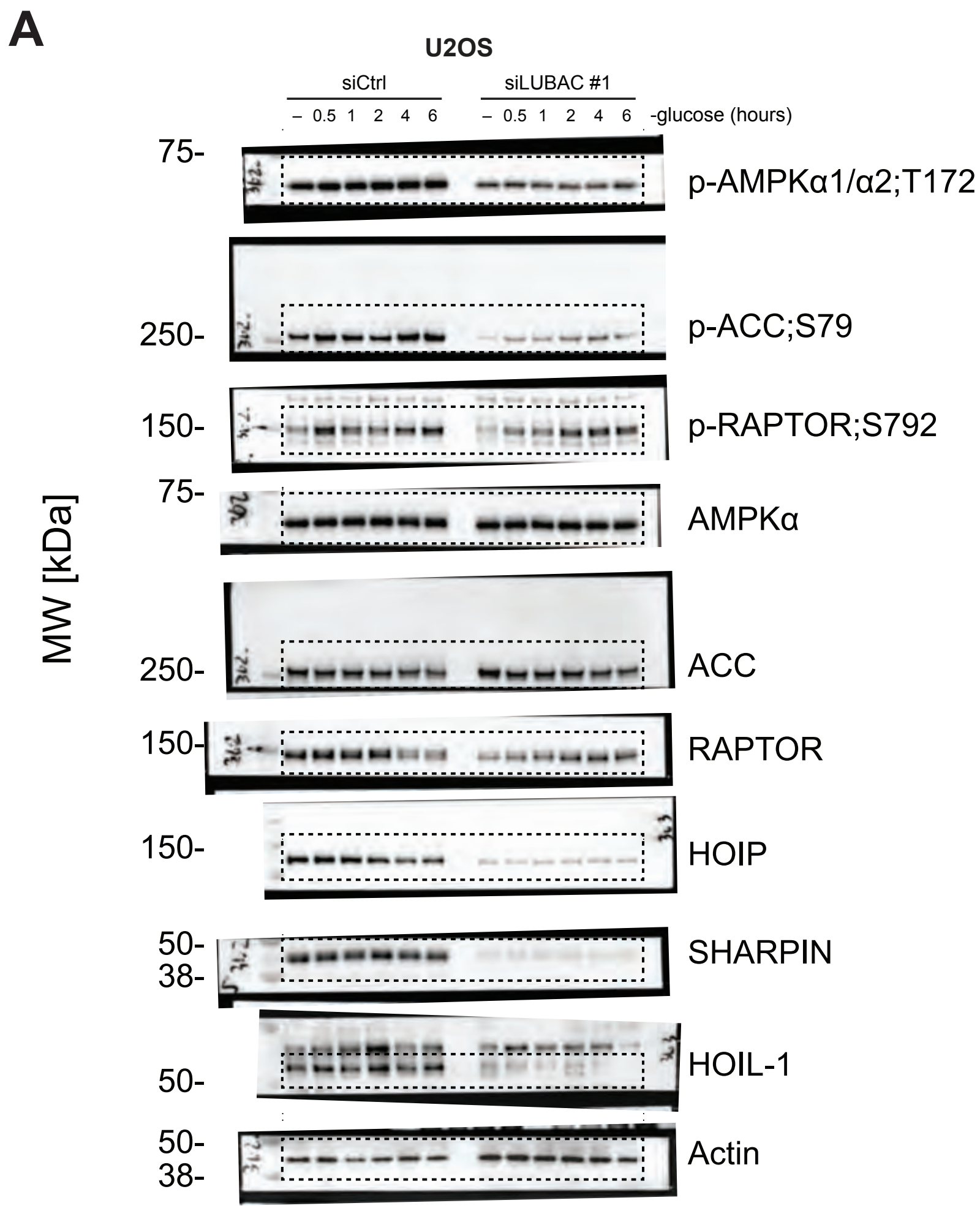

Supplementary Figure 2

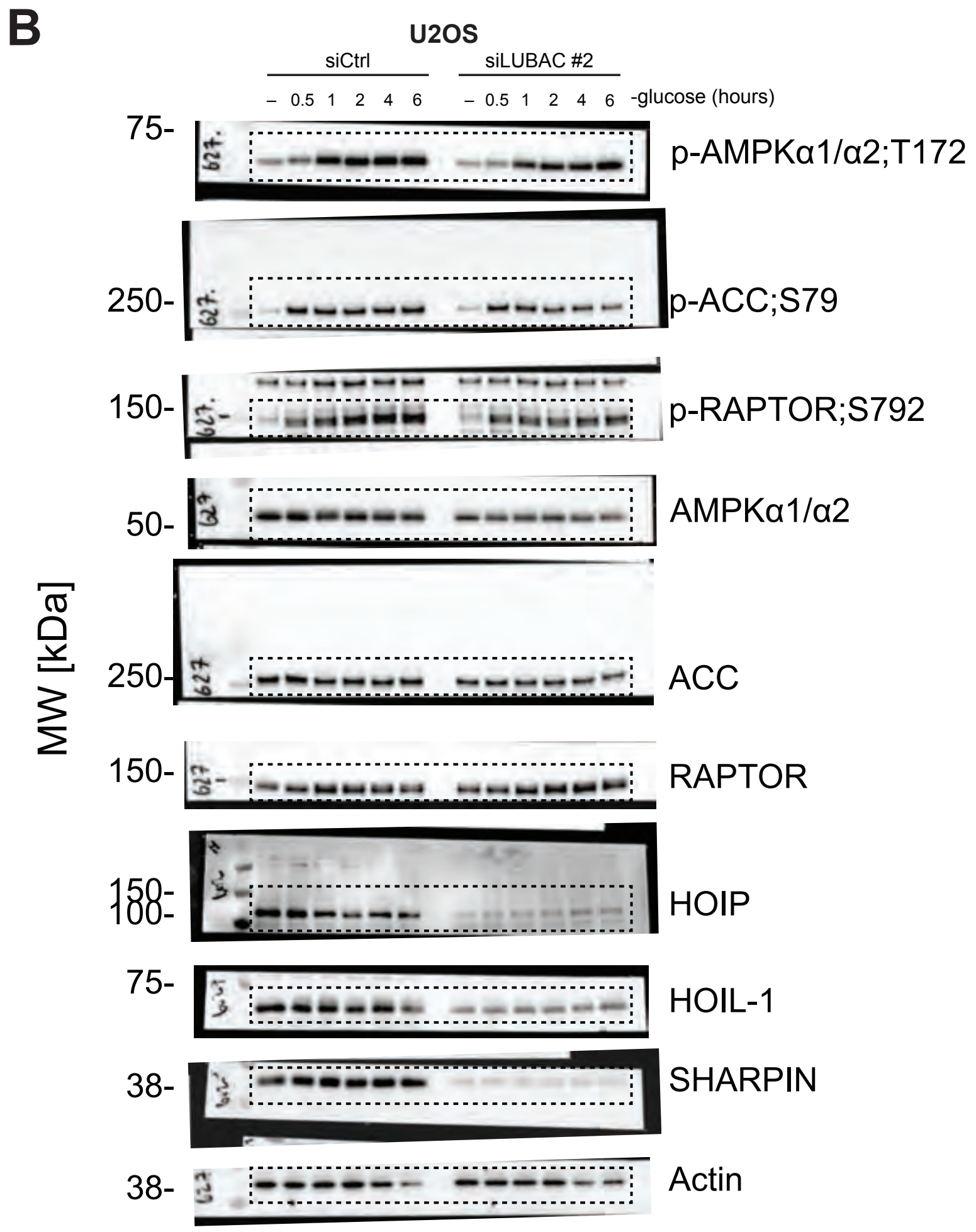

Supplementary Figure 2

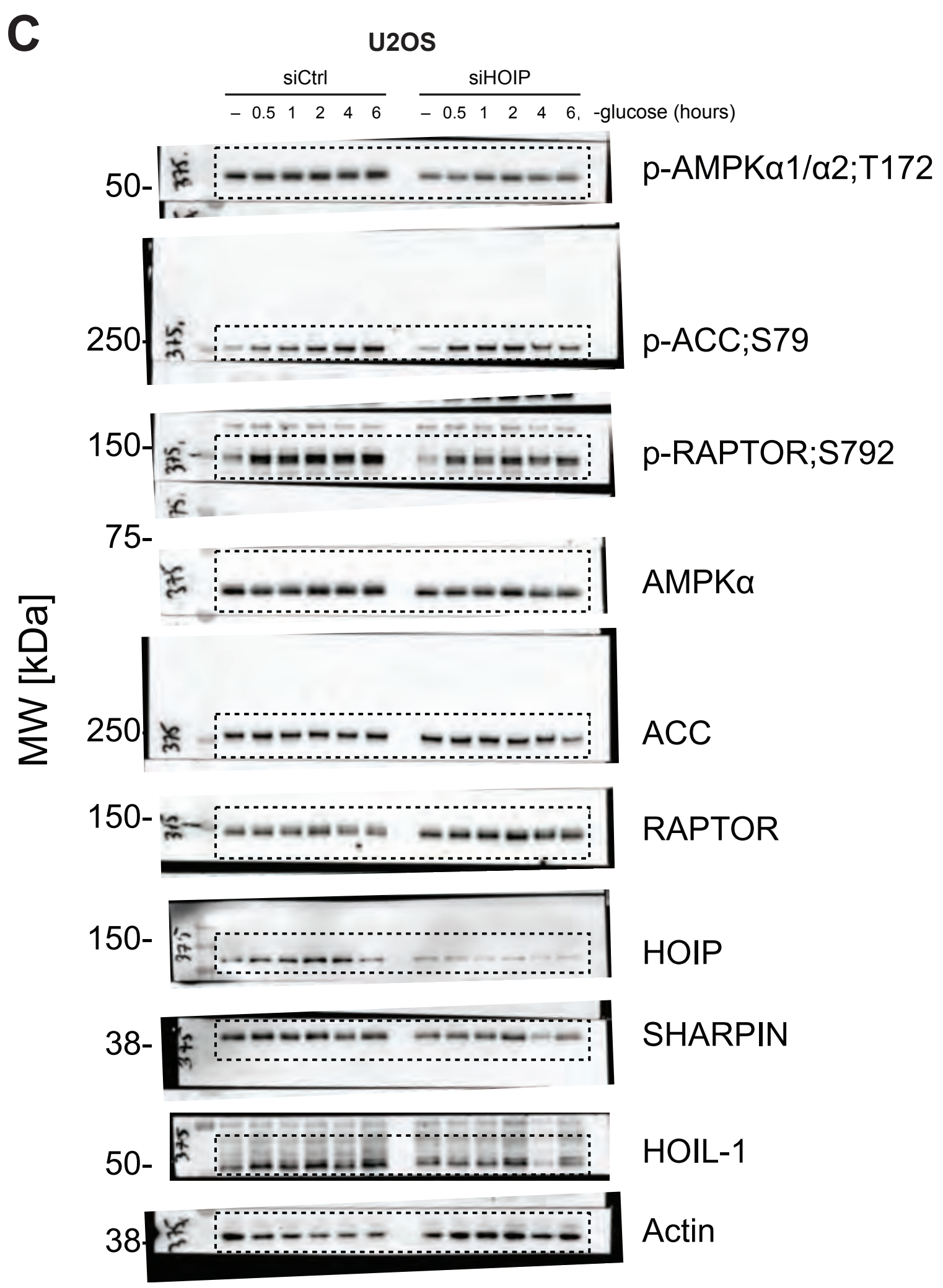

Supplementary Figure 2

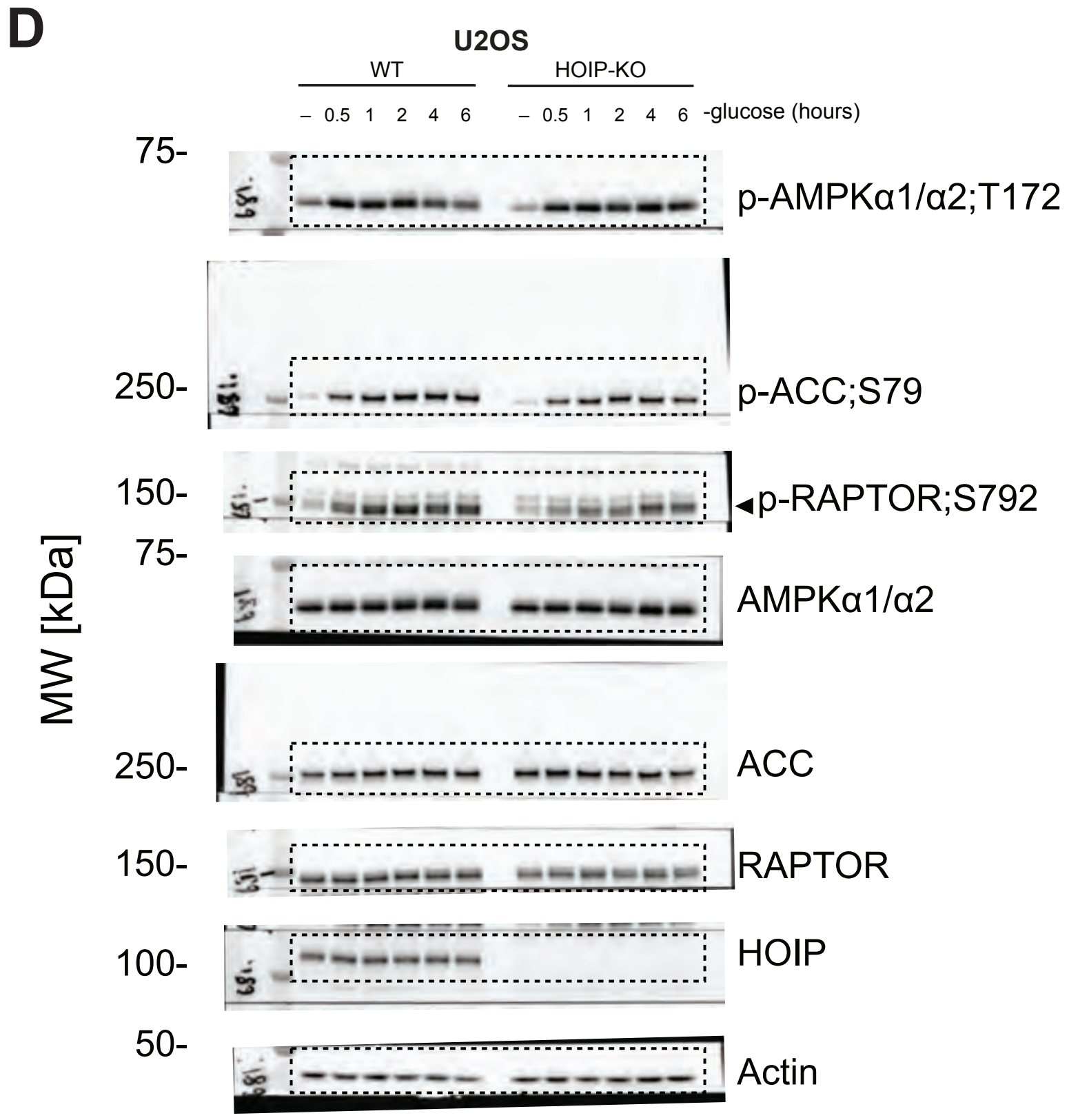

Supplementary Figure 2

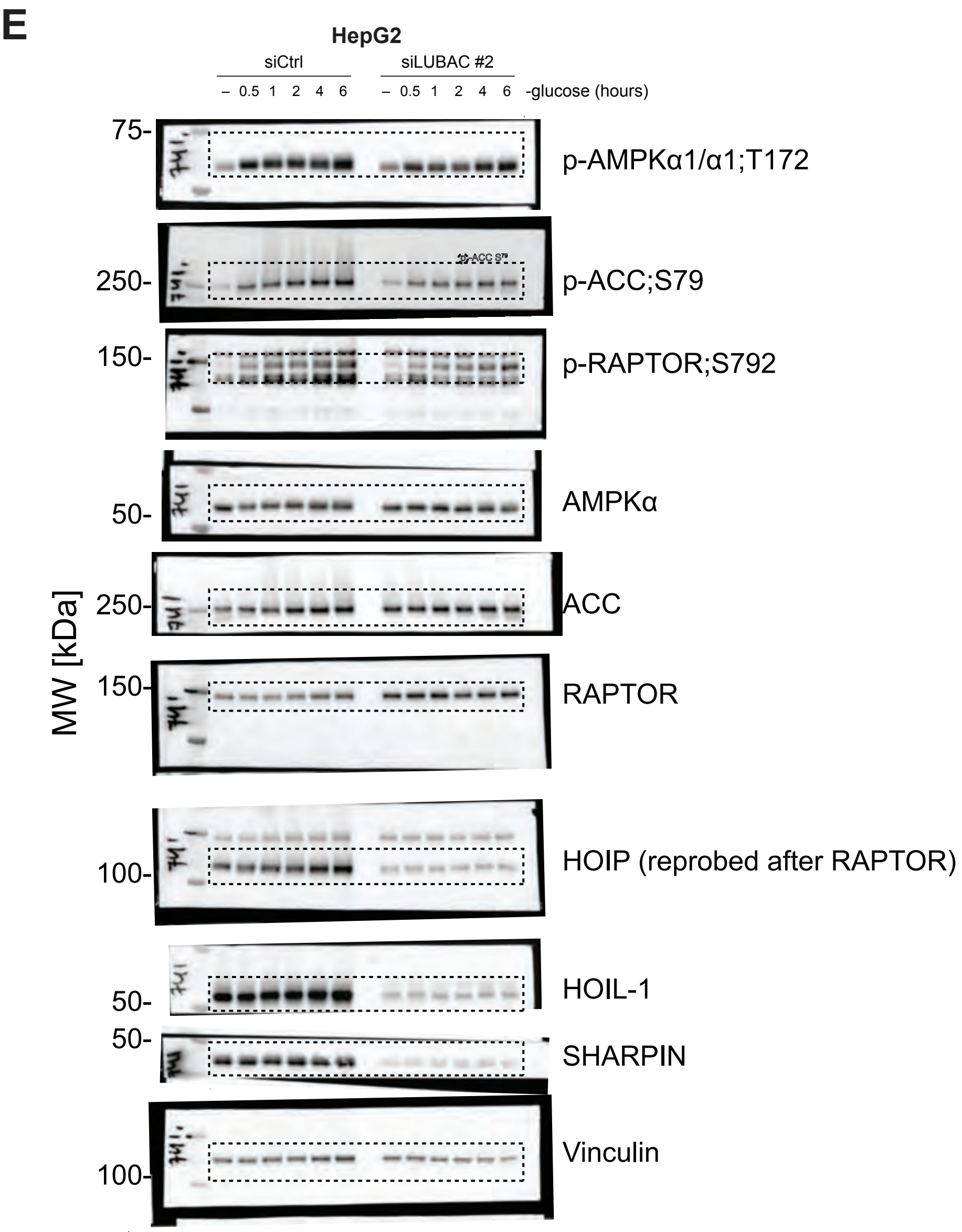

Supplementary Figure 2

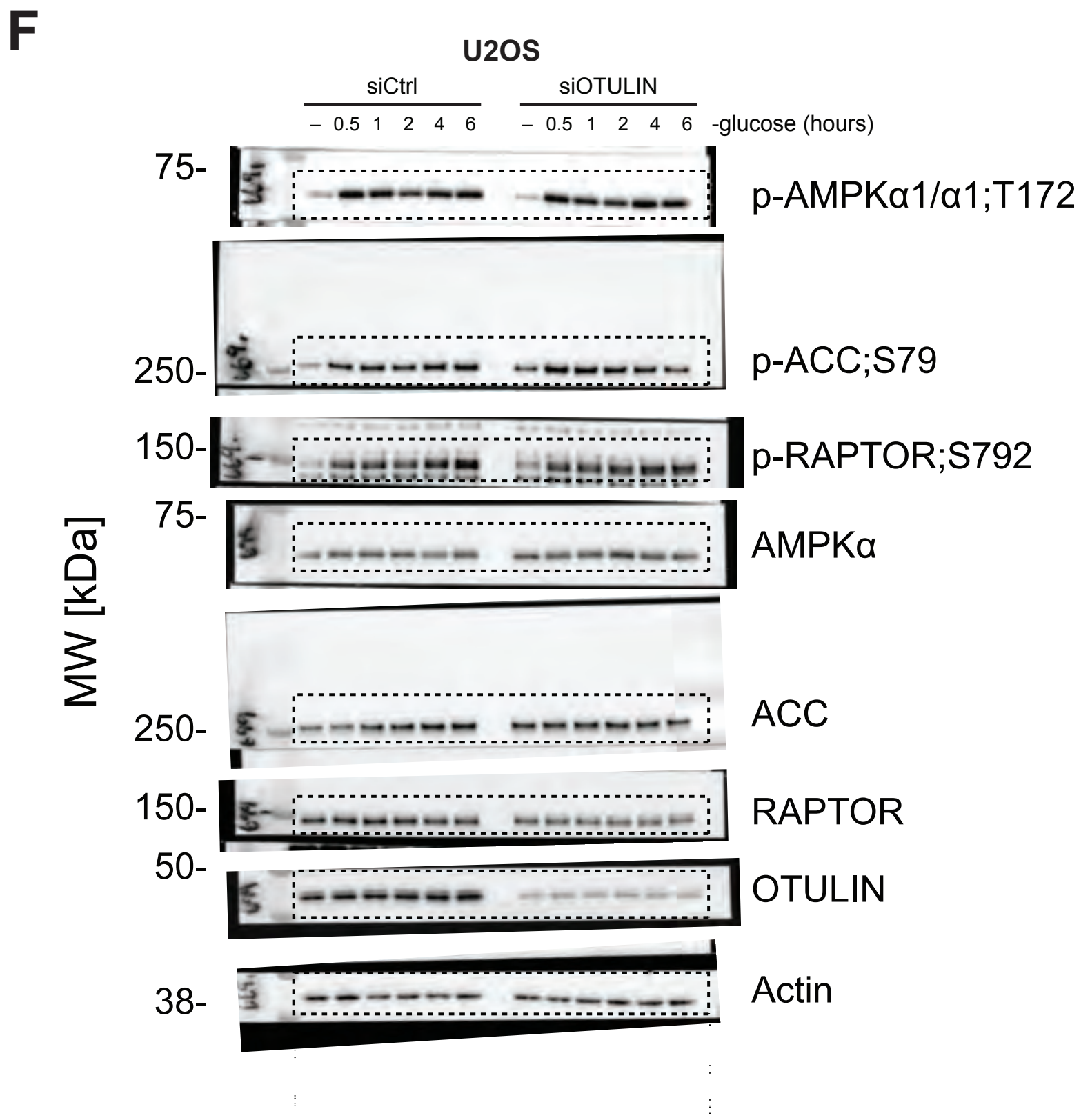

Supplementary Figure 3

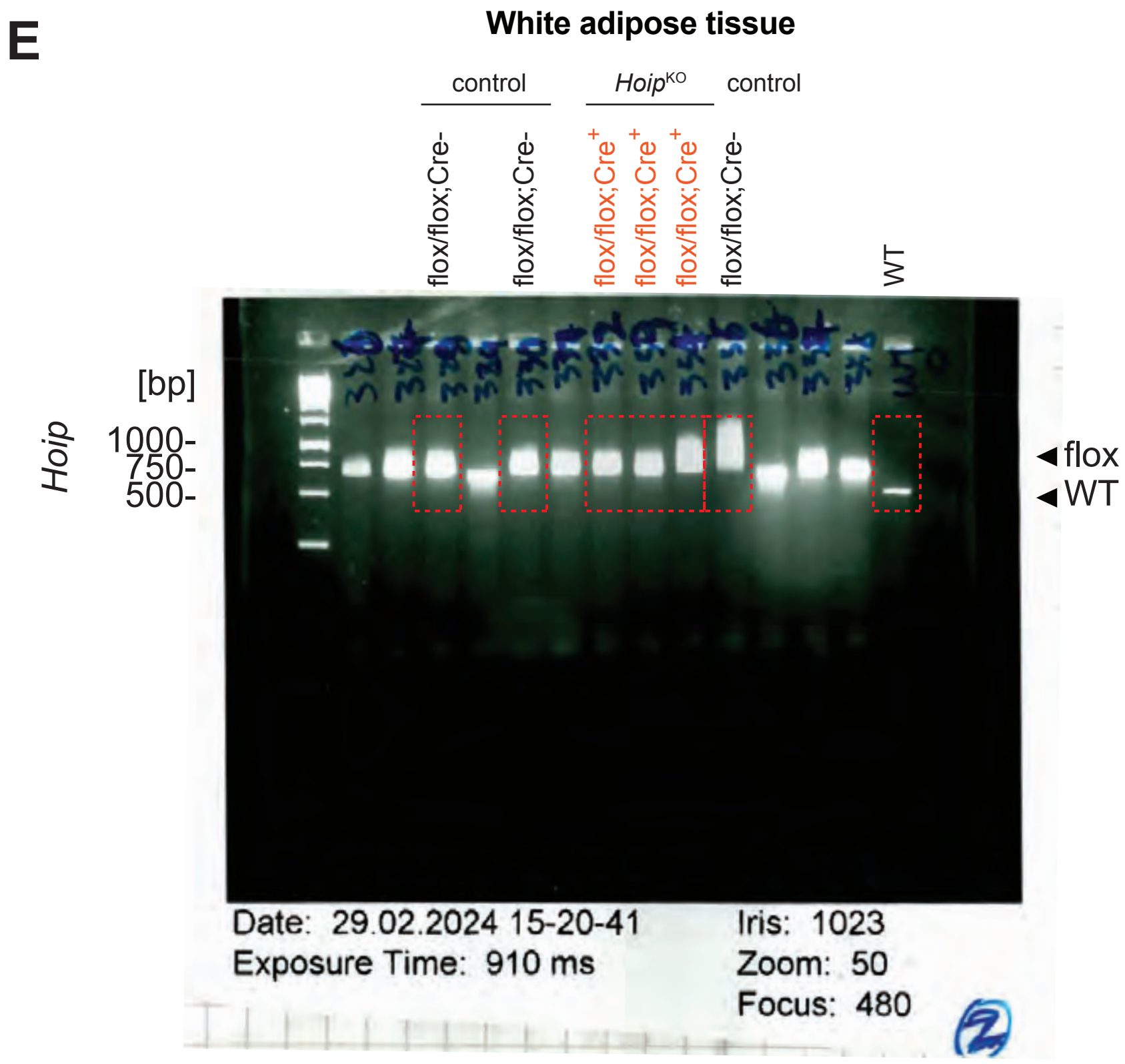

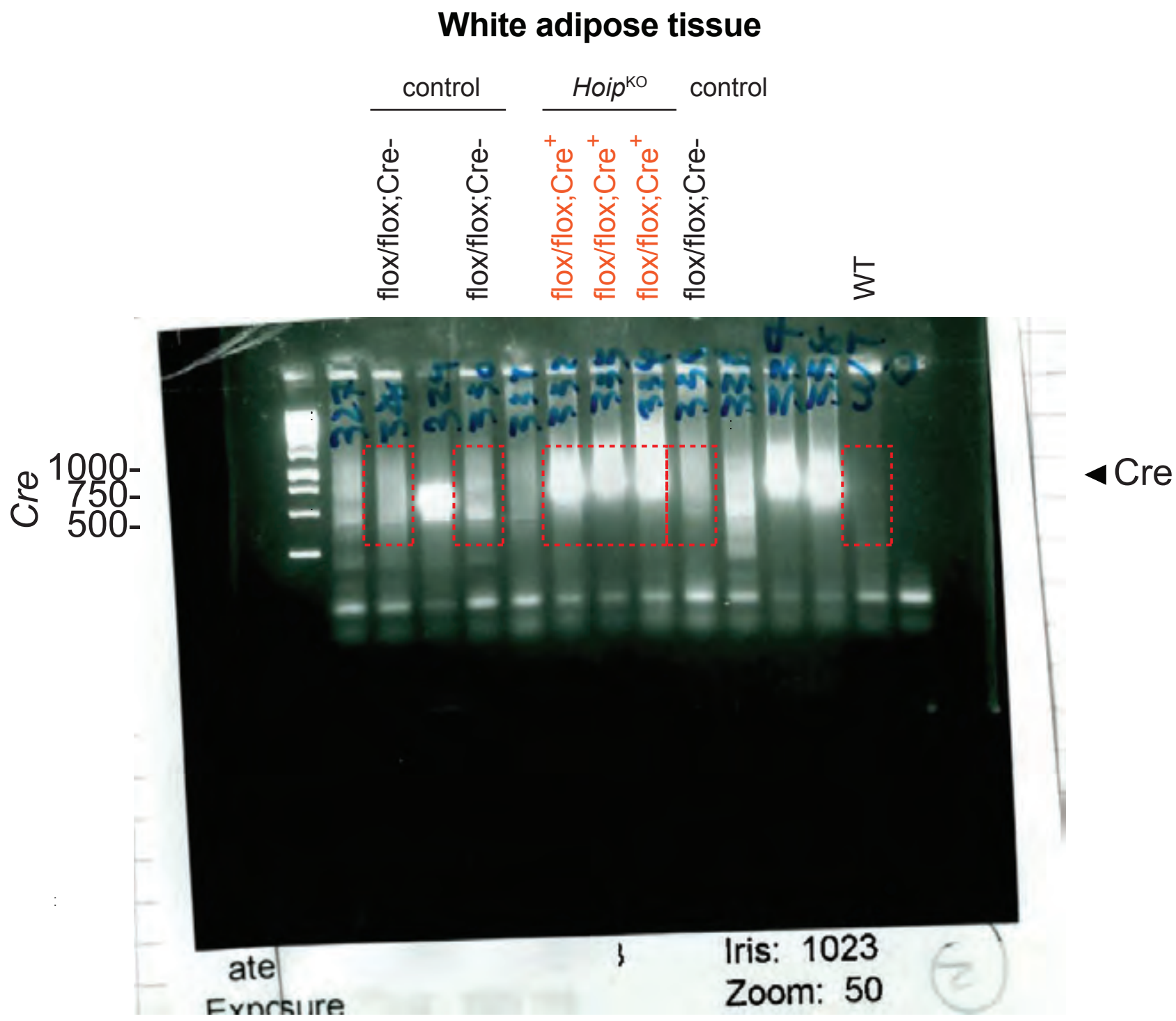

Supplementary Figure 3

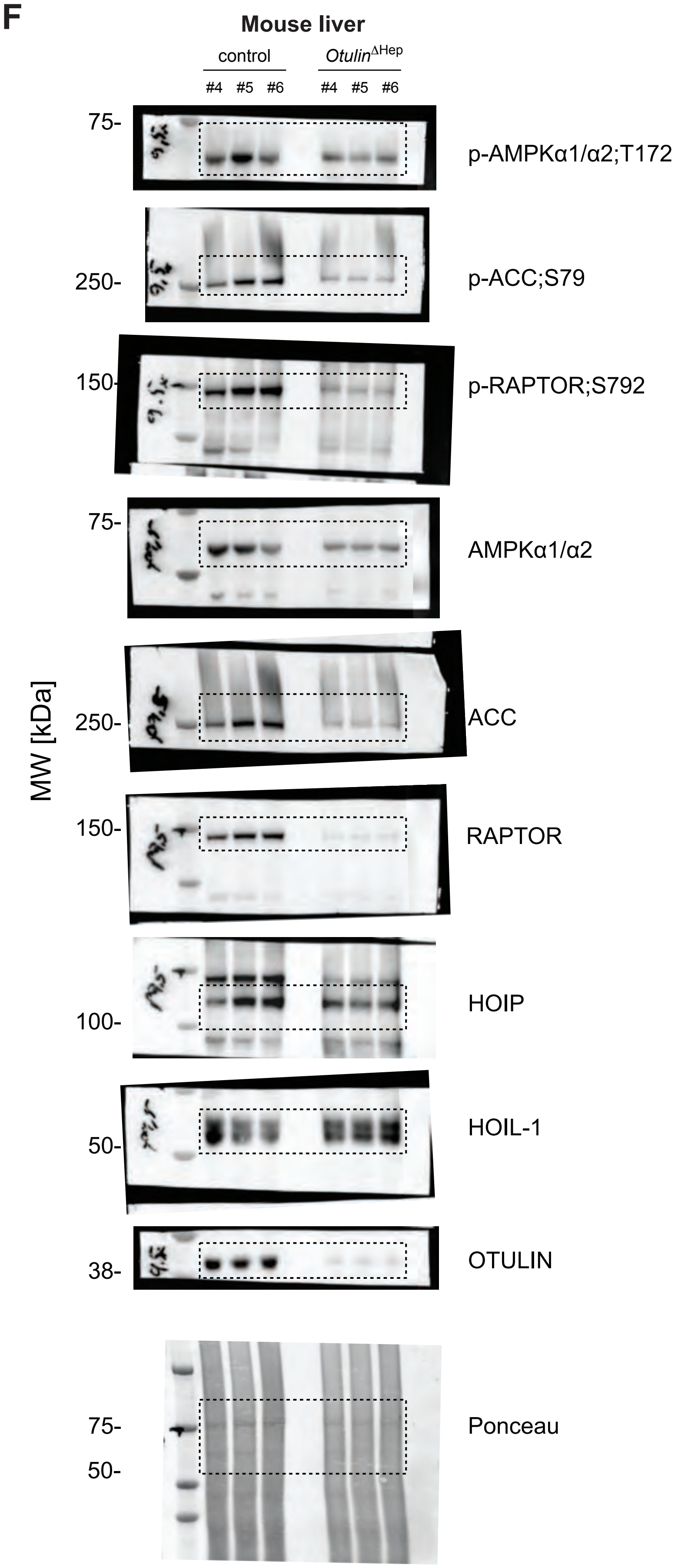

Supplementary Figure 4

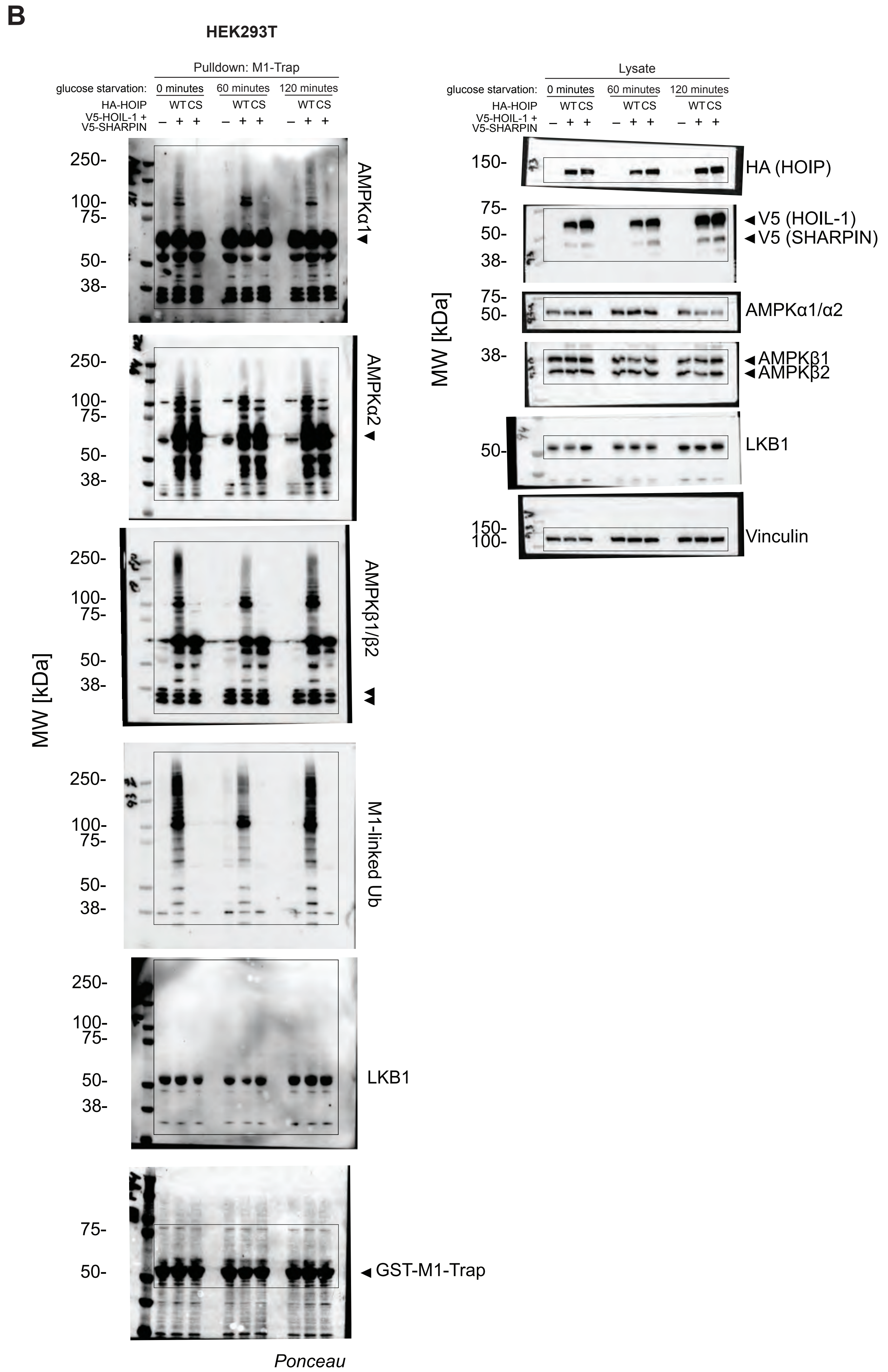

Supplementary Figure 5

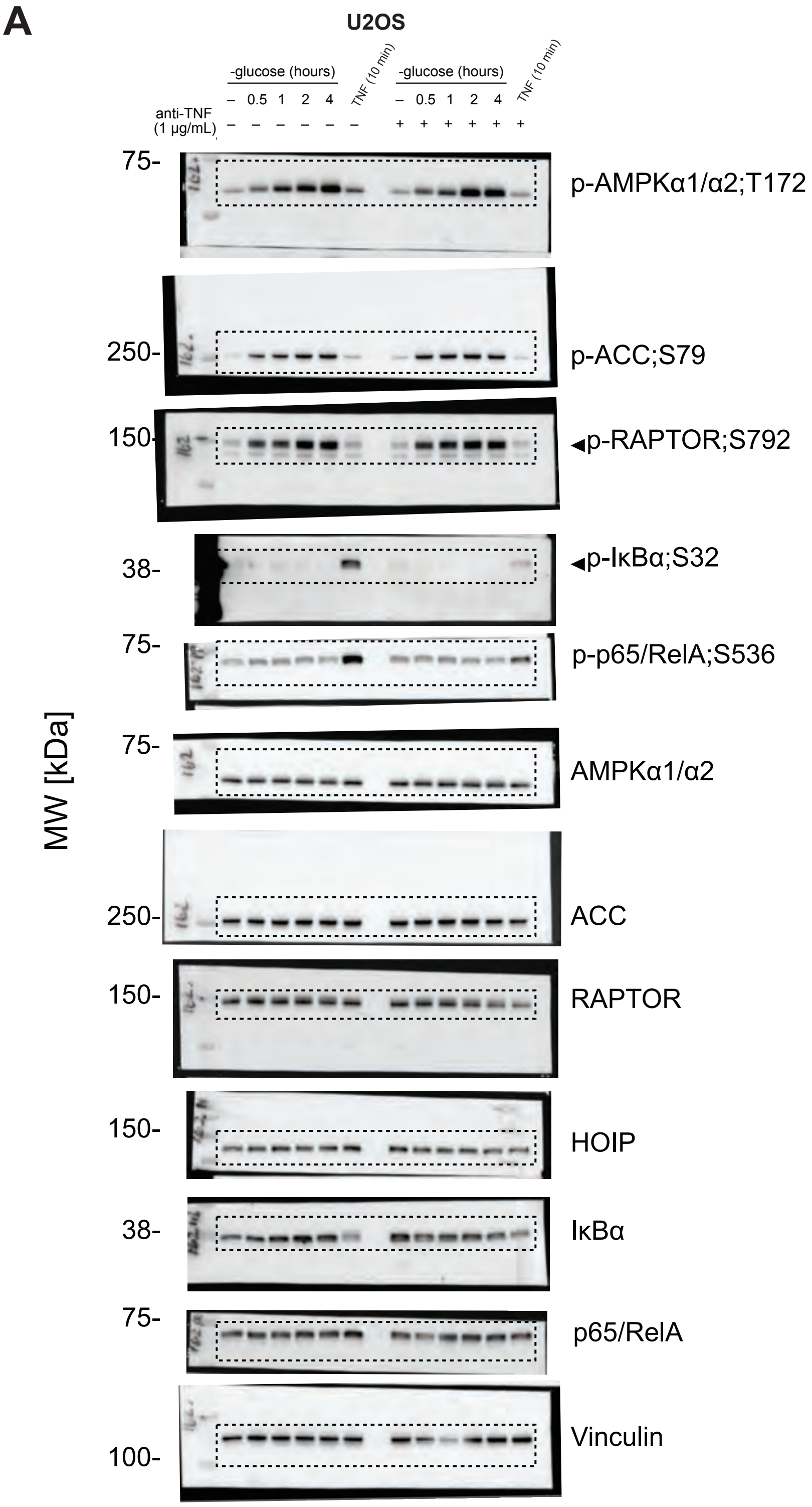

Supplementary Figure 5

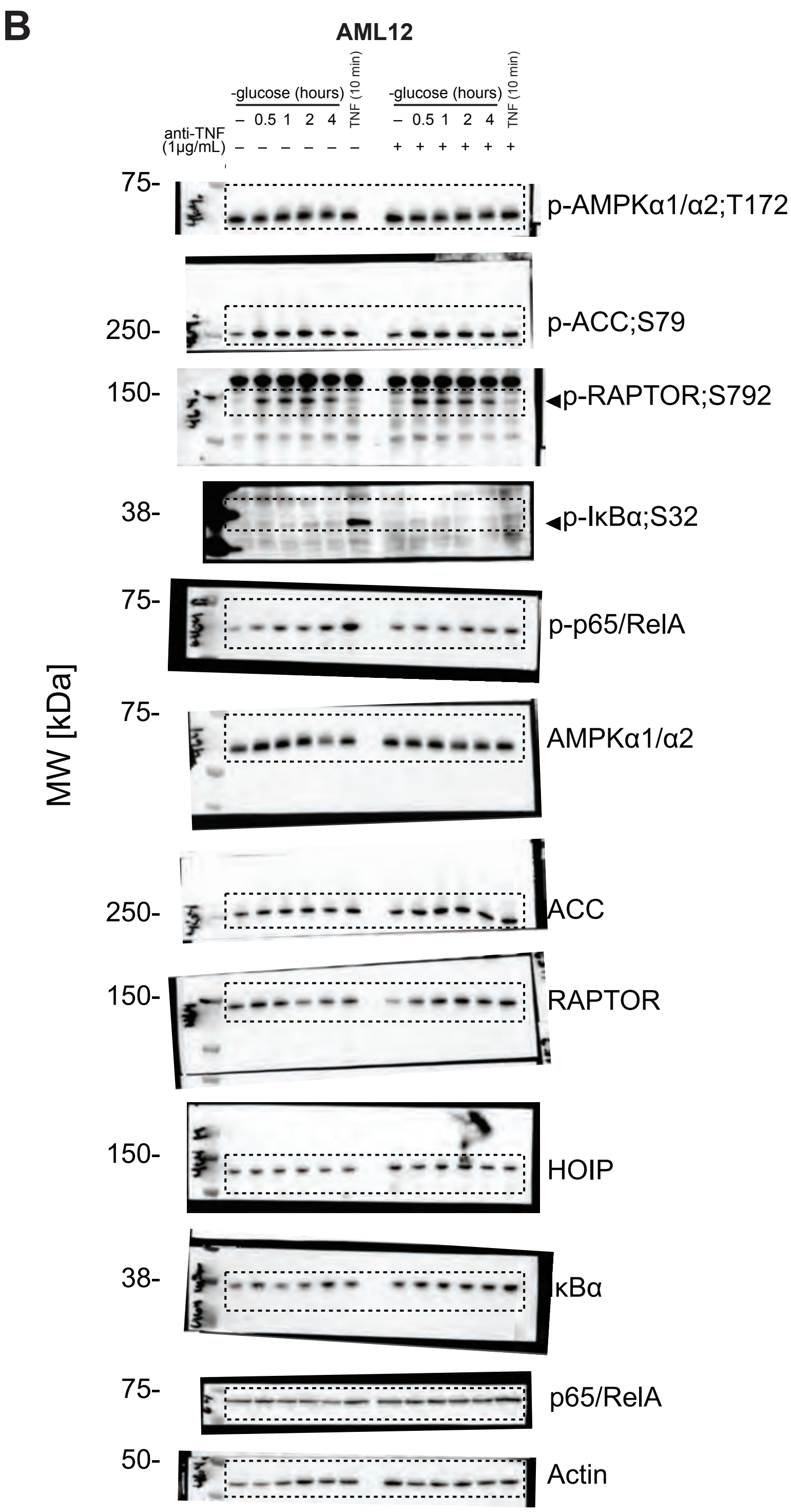

Supplementary Figure 5

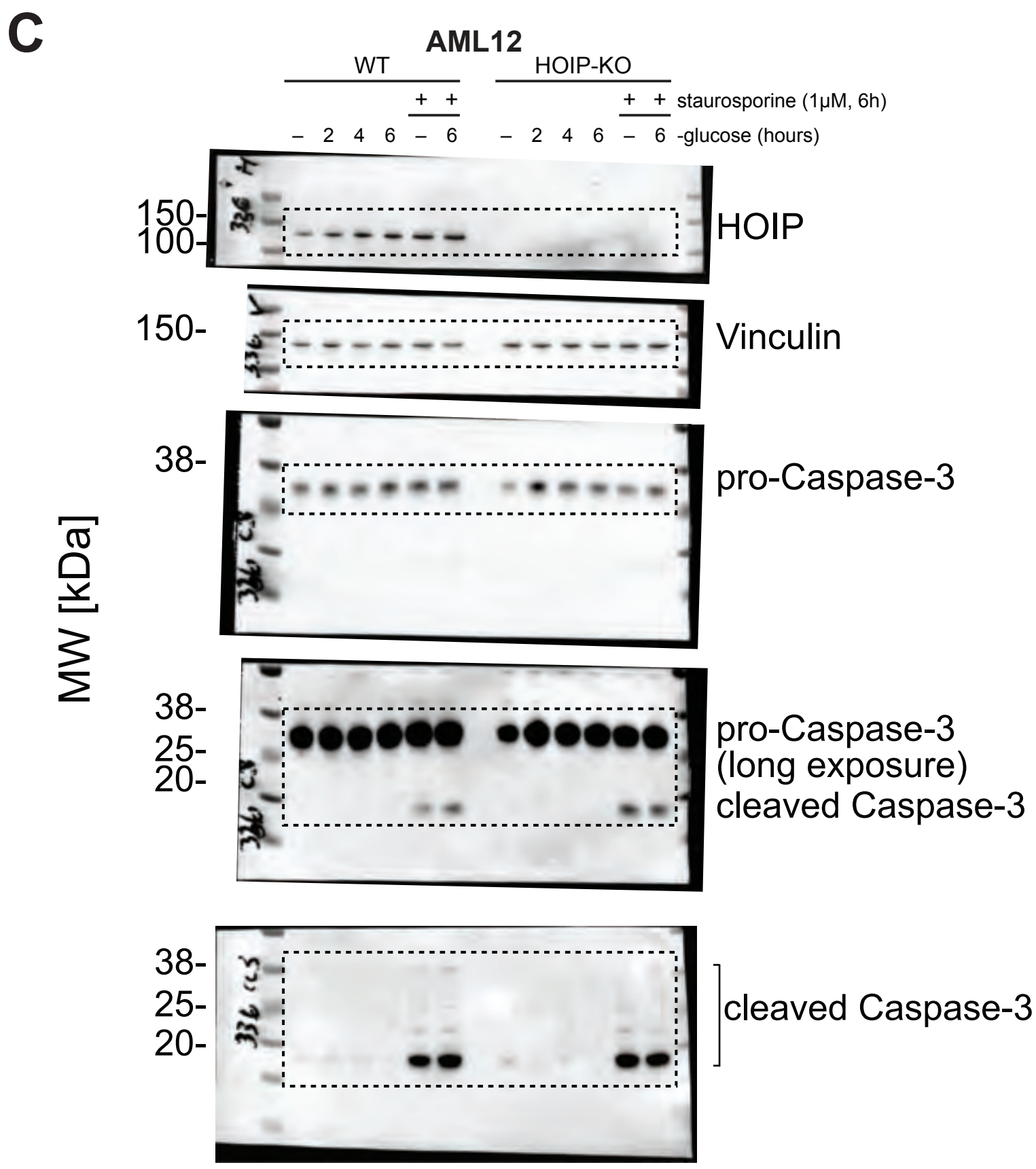

Supplementary Figure 5

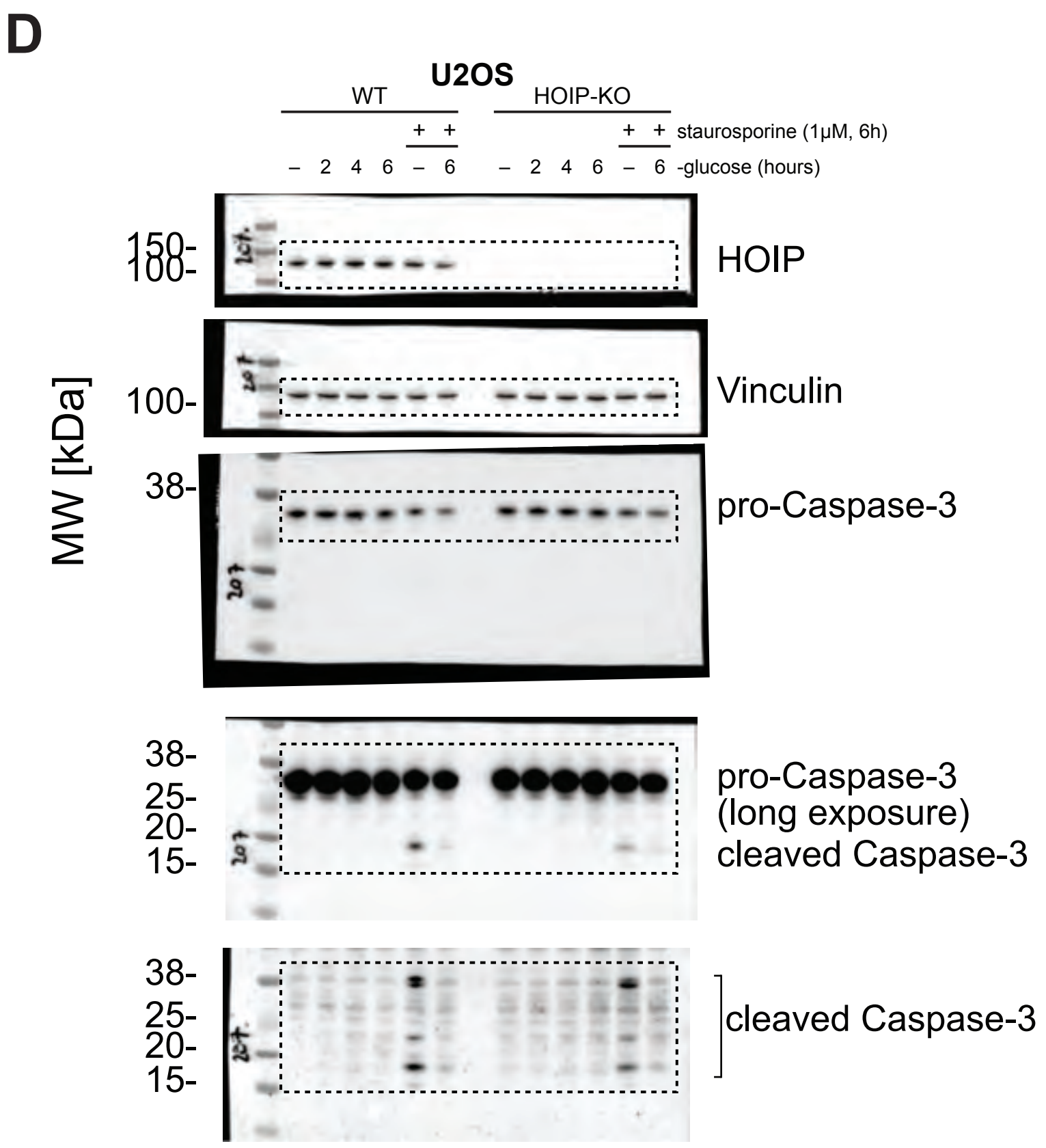

Supplementary Figure 5

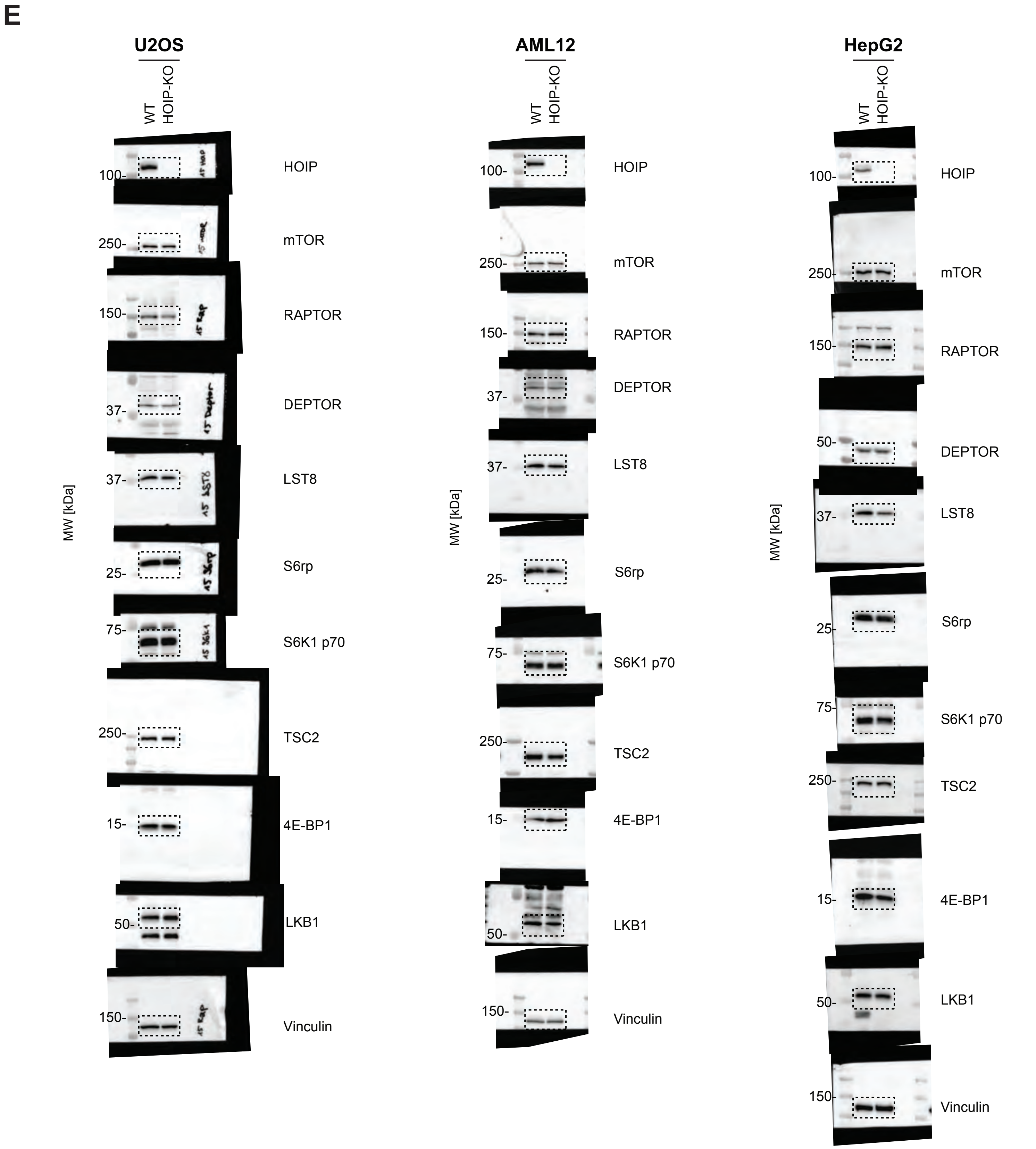

Supplementary Figure 5

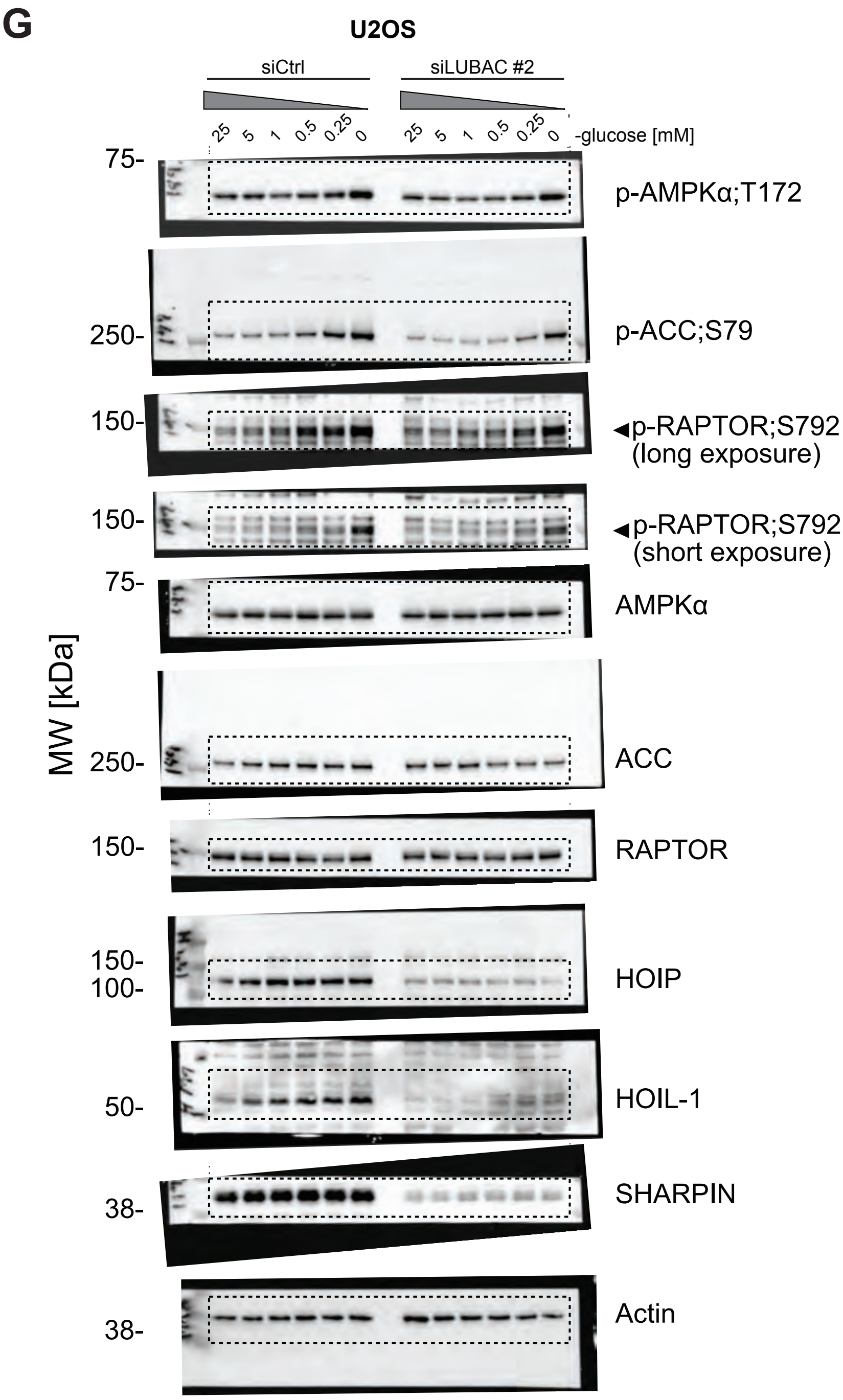

Supplement: Supplementary file 3 [file 41418_2026_1675_MOESM3_ESM.pdf]
